# Rational Design, Synthesis and In Vitro Activity of Diastereomeric *Cis*-/*Trans*-3-Substituted-3,4-Dihydroisocoumarin-4-Carboxylic Acids as Potential Carnitine Acetyltransferase Inhibitors

**DOI:** 10.3390/molecules30153159

**Published:** 2025-07-28

**Authors:** Savina Stoyanova, Milen G. Bogdanov

**Affiliations:** Faculty of Chemistry and Pharmacy, Sofia University St. Kl. Ohridski, 1 J. Bourchier Blvd, 1164 Sofia, Bulgaria; savinais@uni-sofia.bg

**Keywords:** carnitine acyltransferase, fatty acid oxidation, isocoumarins, metabolism, enzyme inhibition

## Abstract

This study explores a series of 3,4-dihydroisocoumarins as potential inhibitors of fatty acid oxidation through rational design, synthesis and in vitro evaluation. The compounds studied were designed as structural analogs of the natural substrates of carnitine acetyltransferase (CAT) and other enzymes in the carnitine transferase family, which play a crucial role in fatty acid metabolism. Comparative in vitro analyses revealed that the presence of an alkyl substituent at position 3 of the heterocyclic core, along with its chain length, significantly influences inhibitory activity, yielding IC_50_ values in the micromolar range. Kinetic studies of one of the most potent compounds—*cis*- and *trans*-3-decyl-6,7-dimethoxy-3,4-dihydroisocoumarin-4-carboxylic acids—demonstrated mixed inhibition of CAT, with *K_i_* values of 130 μM and 380 μM, respectively. These findings underscore the therapeutic potential of the compounds under investigation in modulating fatty acid catabolism, with possible applications in treating metabolic disorders.

## 1. Introduction

Carnitine acyltransferases (CTs) are a family of enzymes essential for energy production in human and animal cells. They regulate fatty acid oxidation by catalyzing the reversible transfer of acyl groups between L-carnitine and coenzyme A (CoA). This enzyme family comprises three members—carnitine acetyltransferase (CAT), carnitine octanoyltransferase (COT) and carnitine palmitoyltransferase (CPT)—each responsible for transporting fatty acids of varying chain lengths across cellular compartments, including the cytosol, mitochondria and peroxisomes [1,2,3,4,5]. Given their critical role in fatty acid metabolism, inhibiting CTs activity has emerged as a promising therapeutic strategy for various chronic diseases linked to excessive fatty acid breakdown. These conditions include cardiovascular diseases [6,7], diabetes [8], kidney and liver diseases [9,10], psychiatric disorders [11], neurodegenerative diseases [12] and certain cancers [13,14,15,16,17,18,19,20].

**Figure 1:**
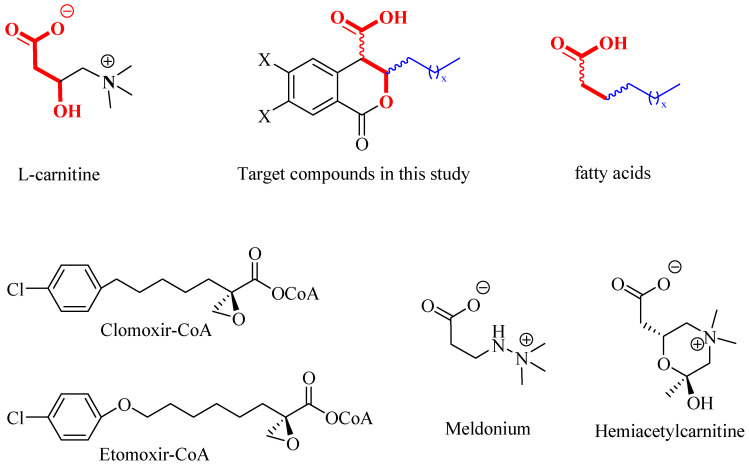
Structure of Clomoxir-CoA and Extomoxir-CoA, Meldonium and Hemiacetylcarnitine.

As part of an ongoing project focused on synthesizing potential inhibitors of CTs [21], we directed our attention to a class of natural compounds—3,4-dihydroisocoumarins, which are prominent due to their broad spectrum of biological activities [22,23,24,25,26,27,28,29,30,31,32,33,34]. When appropriately substituted, these compounds can be regarded as molecular hybrids that integrate structural fragments of the natural substrates of carnitine acyltransferases—the L-carnitine fragment and the fatty acid residue (see Figure 1). Moreover, they resemble the structure of well-known CT inhibitors, such as Clomoxir [35,36], Etomoxir [37,38], Meldonium [39] and Hemiacetylcarnitine [40], which additionally suggests their potential.

In the present study, we synthesized a series of 3,4-dihydroisocoumarin derivatives with a carboxylic group at position 4 and varied substituents (alkyl or aryl) at position 3 of the heterocyclic moiety. We further assessed their inhibitory potential towards CAT as a model enzyme. To the best of our knowledge, this investigation is the first to explore the possibility of such compounds serving as metabolic modulators by inhibiting CAT.

## 2. Results

### 2.1. Rational Design

As can be seen from Figure 1, the target 3-substituted 3,4-dihydroisocoumarin-4-carboxylic acids exhibit structural similarity with the native substrates of CTs. The free carboxyl group and the oxygen atom in the β-position relative to it (given in red) resemble the L-carnitine molecule, while the alkyl substituent at C3 (given in blue) resembles those of the fatty acid’s hydrocarbon chain. Furthermore, the presence of a lactone ring would lead to reduced reactivity and increased selectivity. The latter suggests their lower toxicity compared to that demonstrated by the well-known CT inhibitors Etomoxir and Clomoxir, which contain a highly reactive oxirane ring [35,36,37,38].

**Scheme 1:**
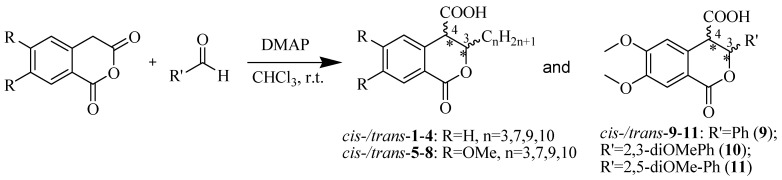
Synthesis of *cis*- and *trans*-3,4-dihydroisocoumarin-4-carboxylic acids **1**–**11**.

Based on the above analysis, we hypothesized that the target compounds may interact with both active sites of CAT—specifically for L-carnitine and fatty acids—thereby leading to more effective inhibition by simultaneously blocking two regions within its active center. To test this hypothesis, we designed hypothetical structures of 3,4-dihydroisocoumarin-4-carboxylic acids with different substituents at position 3 of the benzopyranone skeleton (see Scheme 1). We chose propyl, heptyl, nonyl and decyl as alkyl groups to evaluate the impact of the alkyl chain length as a key factor and included some aryl-substituted compounds (phenyl, 2,3-dimethoxyphenyl and 2,5-dimethoxyphenyl) to study the effect of substituent type (see Scheme 1). We also added methoxy groups at positions 6 and 7, hypothesizing that this might result in additional hydrogen bonding interactions or influence others, such as π-π stacking or π-cation interactions. Relying on this rationale, we believed that our compounds would be more effective inhibitors of CAT than established ones such as Meldonium [39], which targets only one site in the enzyme’s active center, or Etomoxir and Clomoxir [41], which are suicidal inhibitors causing hepatotoxicity.

### 2.2. Synthesis and Characterization

Scheme 1 outlines the synthesis of the target 3-substituted-3,4-dihydroisocoumarin-4-carboxylic acids as well as the substitution pattern. We employed the straightforward method previously reported by Bogdanov and Palamareva [42] for synthesizing 3-aryl-3,4-dihydroisocoumarin-4-carboxylic acids from homophthalic anhydride and aromatic aldehydes, which we had also recently adapted for synthesizing 3-alkyl-substituted derivatives, particularly *cis*- and *trans*-**5**–**8** [43].

During the reaction, two new stereogenic centers form at atoms C-3 and C-4. This results in σ-diastereomerism in these compounds, with the *cis* and *trans* arrangement of the substituents relative to the benzopyranone ring system. In the majority of cases, the resulting diastereoisomeric mixtures were successfully separated by flash chromatography, and the products were isolated in pure, crystalline form. Because of the similar behavior of the two diastereoisomers, 3-propyl derivatives **1** and **5**, as well as the 2,5-dimethoxyphenyl substituted ones (compd. **11**), were isolated, characterized and tested as isomeric mixtures. Compound 1 was isolated as two mixtures, **M1-1** and **M2-1**, with the *cis* isomer making up 60% and 35% of each mixture, respectively. Compound 5 was obtained as two mixtures, **M1-5** and **M2-5**, with the *cis* isomer comprising 90% and 30% of each mixture, respectively. The *cis* isomer of compound **11** was successfully isolated, but the *trans* isomer was obtained as a mixture (**M-11**) with a 60/40 *cis*/*trans* ratio.

The structure of the synthesized compounds was unambiguously determined using various spectral techniques, including ^1^H, ^13^C, DEPT-135 NMR and HRMS analysis. The interpretation of spectral data aligns with literature data [42,43,44,45,46]. For the 6,7-dimethoxy-substituted analogues, the spectral data include two singlets for the aromatic protons H-8 and H-5, two singlets for the methoxy groups and multiplet signals for the methyl and methylene groups in the alkyl chain at C-3. Additional signals reflecting differences in configuration and allowing the determination of substances as *cis* and *trans* diastereomers are clearly distinguishable multiplets for H-3 and doublets for H-4. The ratios between *cis* and *trans* isomers in the case of racemic mixtures were determined from the integrals of signals for the protons H-3 and H-4. All compounds demonstrate conformational flexibility; however, a comprehensive discussion of this subject exceeds the scope of this study (see Refs. [43,44] for additional details). Spectral data are included in the Supplementary Materials.

### 2.3. Biological Assessment

**Figure 2:**
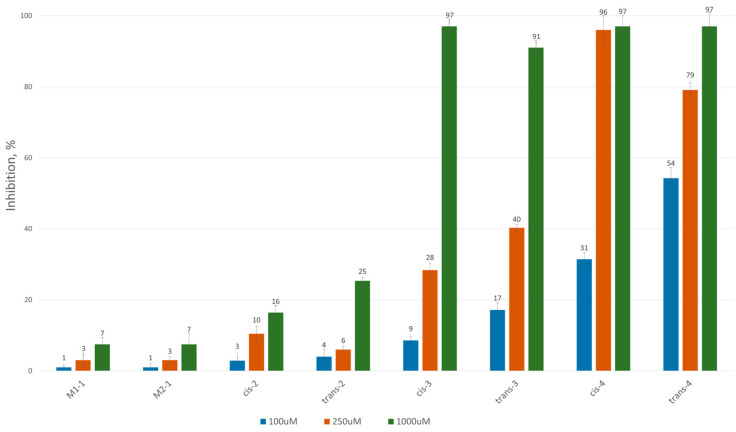
CAT inhibition (%) in the presence of (±)-3-alkyl-3,4-dihydro-3,4-dihydroisocoumarin-4-carboxylic acids.

**Figure 3:**
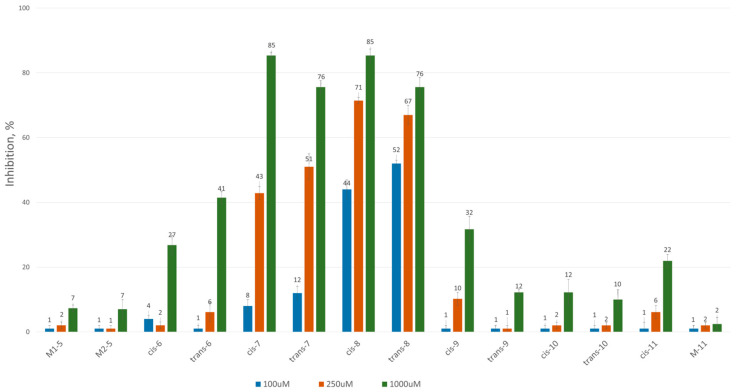
CAT inhibition (%) in the presence of (±)-3-alkyl/aryl-3,4-dihydro-6,7-dimethoxy-3,4-dihydroisocoumarin-4-carboxylic acids.

To assess the inhibitory potential of the synthesized compounds against CAT, we conducted an initial screening at three concentrations: 100 μM, 250 μM and 1000 μM. Readings were taken within the first minute after the reaction started, and inhibition was measured using a kinetic method, as described in the Experimental section. The results are summarized and presented as a percentage of inhibition in Figure 2 and Figure 3 for the 3-alkyl-3,4-dihydroisocoumarin-4-carboxylic acids and the 3-alkyl/3-aryl-6,7-dimethoxy-3,4-dihydroisocoumarin-4-carboxylic acids, respectively.

As shown in Figure 2 and Figure 3, all tested compounds exhibit promising inhibitory properties, with some demonstrating significant effects at micromolar concentrations. These findings confirm that activity depends on both the substituent type and alkyl chain length, supporting our initial hypothesis. At the highest tested concentration (1 mM), the nonyl and decyl derivatives induce near-complete inhibition, whereas propyl, heptyl and their aromatic analogues show lower activity. Notably, the inhibitory potency appears independent of stereochemistry and substitution pattern, as evidenced by the similar effects demonstrated by the *cis*- and *trans*-diastereomeric pairs and methoxy-substituted and unsubstituted derivatives at positions 6 and 7.

The most potent Inhibitors in this series were the *cis*- and *trans*-3-decyl-3,4-dihydroisocoumarin-4-carboxylic acids, with IC_50_ values of approximately 250 μM—an order of magnitude lower than the positive control, Meldonium (IC_50_ = 11.4 mM, Ref. [21]).

Given carnitine acetyltransferase’s high affinity for short-chain fatty acids (C_2_–C_4_), the observed correlation between increasing hydrocarbon chain length and enhanced inhibitory activity suggests an uncompetitive or mixed-type inhibition mechanism. To validate this hypothesis, we conducted additional kinetic studies as detailed in the Experimental section on the most active diastereomeric pair, *cis*-**8** and *trans*-**8**. These experiments allowed us to identify the inhibition mechanism and calculate *K_m_*, *V_max_* and *K_i_*. We used SigmaPlot version 12.5 (Systat Software Inc., San Jose, CA, USA), which includes modules for regression analysis and various inhibition models. This software also lets us choose the mechanism of inhibition with the highest correlation factors (coefficient of determination (R^2^) and Akaike information coefficient (AIC)) and the lowest S_y,x_ value. The numerical results are summarized in Table 1, while the corresponding Lineweaver–Burk and Michaelis–Menten kinetic plots are available in the Supplementary Materials. Our findings indicate that *cis*-**8** and *trans*-**8** are mixed-type inhibitors with *K_i_* values of 130 μM and 377 μM, respectively. The trans isomer demonstrated a cooperative effect (α = 0.21, where α < 1), indicating an increased inhibitory activity in the presence of the substrate. Conversely, the *cis* isomer functions as an allosteric inhibitor, attaching to a different site on the enzyme and inducing a conformational change that reduces the enzyme’s affinity for its substrate.

The results obtained demonstrate the possibility of the studied compounds being applied as metabolic modulators and their potential for the treatment of systemic inflammatory processes, ischemic disease, diabetes and some types of cancer. It would be of interest to study the individual enantiomers, which is also the subject of upcoming research.

## 3. Materials and Methods

### 3.1. General

All chemicals and CAT (isolated from pigeon breast muscle, ammonium sulfate suspension, CAS Number: 9029-90-7) were obtained from Sigma-Aldrich (FOT, Sofia, Bulgaria). Analytical-grade organic solvents were used without further purification. Thin-layer chromatography (TLC) was performed on 0.2 mm pre-coated aluminum plates with silica gel 60 and a fluorescence indicator (Alugram^®^ SIL G/UV254, Macherey-Nagel, Merck, Darmstadt, Germany). Column chromatography was performed on Horizon High Performance FLASH chromatography system – HPFC, (Biotage, Uppsala, Sweden) with cartridges filled with Silica gel 60 (particle size—0.06–0.2 mm (70–230 mesh), MACHEREY-NAGEL, Düren, Germany). NMR spectra were recorded on a Bruker Avance III HD (Bruker BioSpin GmbH, Rheinstetten, Germany) at 500 MHz for ^1^H and 126 MHz for ^13^C, using DMSO-_d6_ as the solvent. Chemical shifts (δ) are reported in ppm, and *J* values are given in Hz. Biological assessment was performed on ELISA Reader Biotek 800TS (Biotek Instruments, Inc., ELTA90, Sofia, Bulgaria). High-Resolution Mass Spectra (HRMS) were obtained on a Shimadzu LCMS-9050 (Shimadzu Handels GmbH., Korneuburg, Austria).

### 3.2. Synthesis

The aldehyde (1 equiv.) was added to a solution of 1.1 equiv. of the corresponding homophthalic anhydride in 10 mL dry chloroform, along with 1 equiv. DMAP. The mixture was stirred at room temperature (22–23 °C) for one hour and monitored by TLC. The resultant carboxylic acids were extracted at the end of the reaction with 10% NaHCO_3_, acidified to pH 3 with 18% HCl and extracted with EtOAc. The organic layer was dried with Na_2_SO_4_, and the solvent was evaporated. Diastereoisomers were isolated via column chromatography (petroleum ether/EtOAc = 1/1 + formic acid).

#### 3.2.1. *Cis*- and *Trans*-(±)-3-Propyl-3,4-Dihydro-1-Oxo-1H-Isochromene-4-Carboxylic Acids (**1**)

Homophtalic anhydride (2.00 g, 12.0 mmol) reacted with butanal (0.80 g, 11.0 mmol) in the presence of 1.30 g (11.0 mmol) of DMAP to give white crystals of **1** (1.62 g, 63% yield). After purification, two mixtures—**M1-1** and **M2-1**—were acquired with percentages of the *cis* isomer of 60% and 35%, respectively:

^1^H-NMR (500 MHz, DMSO-_d6_):
*cis* diastereomer: *δ* = 4.73–4.65 (1H, m, 3-CH), 3.98 (1H, d, ^3^*J*_3,4_ = 3.1 Hz, 4-CH)
*trans* diastereomer: *δ* = 4.90 (1H, dt, *J* = 8.8, 4.4 Hz, 3-CH), 4.07 (1H, d, ^3^*J*_3,4_ = 4.0 Hz, 4-CH)
other signals for both diastereomers: *δ* = 12.99 (1H, s, COOH), 7.98–7.91 (1H, m, 8-CH), 7.72–7.61 (1H, m, 6-CH), 7.51 (1H, t, *J* = 7.6 Hz, 7-CH), 7.44 (1H, t, *J* = 7.1 Hz, 5-CH), 1.83–1.64 (1H, m, 1′-CH_2_), 1,64–1,33 (3H, m, 1′-CH_2_, 2′-CH_2_), 0.94 (3H, t, ^3^*J*_2′,3′_ = 7.4 Hz, 3′-CH_3_).

HRMS (ESI) *m*/*z*, calculated for [M-H]^−^ C_13_H_13_O_4_^−^: 233.08193, found [M-H]^−^: 233.08096.

#### 3.2.2. *Cis*- and *Trans*-(±)-3-Heptyl-3,4-Dihydro-1-Oxo-1H-Isochromene-4-Carboxylic Acids (**2**)

Homophtalic anhydride (2.00 g, 12.0 mmol) reacted with octanal (1.40 g, 11.0 mmol) in the presence of 1.30 g (11.0 mmol) of DMAP to give white crystals of **2** (2.70 g, 85% yield). After purification and separation, *cis* and *trans* isomers were acquired:

*cis*-**2**, m.p. = 105–109 °C (from CH_2_Cl_2_: petroleum ether, b.p. = 35–60 °C); R*_f_* = 0.41 (EtOAc: CH_2_Cl_2_ = 2:3); ^1^H-NMR (500 MHz, DMSO-_d6_): *δ* = 12.95 (1H, s, COOH), 7.95 (1H, d, *J* = 7.7 Hz, 8-CH), 7.64 (1H, t, *J* = 7.5 Hz, 5-CH), 7.51 (1H, t, *J* = 7.6 Hz, 7-CH), 7.45 (1H, d, *J* = 7.6 Hz, 5-CH), 4.67 (1H, td, ^3^*J*_3,1′_ = 6.8, ^3^*J*_3,4_ = 3.1 Hz, 3-CH), 3.99 (1H, d, ^3^*J*_3,4_ = 3.0 Hz, 4-CH), 1.81–1.70 (2H, m, 1′-CH_2_), 1.56–1.39 (2H, m, 2′-CH_2_), 1.38–1.19 (8H, m, 3′–6′-CH_2_), 0.87 (3H, t, ^3^*J*_6′,7′_ = 6.6 Hz, 7′-CH_3_). ^13^C NMR (126 MHz, DMSO-_d6_): *δ* = 170.57 (C, C=O, COOH), 164.34 (C, 1C), 138.25 (C, 8aC), 133.63 (CH, 8C), 129.49 (CH, 6C), 128.49 (CH, 7C), 127.71 (CH, 5C), 125.25 (C, 4aC), 78.41 (CH, 3C), 46.68 (CH, 4C), 32.23 (CH_2_), 31.17 (CH_2_), 28.68 (CH_2_), 28.57 (CH_2_), 24.69 (CH_2_), 22.08 (CH_2_), 13.96 (CH_3_, 7′-CH_3_).

HRMS (ESI) *m*/*z*, calculated for [M-H]^−^ C_17_H_21_O_4_^−^: 289.14453, found [M-H]^−^: 289.14390.

*trans*-**2**, m.p = 126–127 °C (from CH_2_Cl_2_: petroleum ether, b.p. = 35–60 °C); R*_f_* = 0.38 (EtOAc: CH_2_Cl_2_ = 2:3); ^1^H-NMR (500 MHz, DMSO-_d6_): *δ* = 13.20 (1H, s, COOH), 7.95 (1H, d, *J* = 7.7 Hz, 8-CH), 7.68 (1H, t, *J* = 7.5 Hz, 6-CH), 7.50 (1H, t, *J* = 7.6 Hz, 7-CH), 7.43 (1H, d, *J* = 7.6 Hz, 5-CH), 4.95–4.82 (1H, m, 3-CH), 4.07 (1H, d, ^3^*J*_3,4_ = 4.1 Hz, 4-CH), 1.63–1.47 (2H, m, 1′-CH_2_), 1.46–1.30 (2H, m, 2′-CH_2_), 1.29–1.03 (8H, m, 3′–6′-CH_2_), 0.83 (3H, t, ^3^*J*_6′,7′_ = 6.8 Hz, 7′-CH_3_). ^13^C-NMR (126 MHz, DMSO-_d6_): *δ* = 171.79 (C, C=O, COOH), 163.09 (C, 1C), 136.47 (C, 8aC), 134.09 (CH, 8C), 129.15 (CH, 6C), 128.63 (CH, 7C), 128.37 (CH, 5C), 124.45 (C, 4aC), 79.06 (CH, 3C), 47.04 (CH, 4C), 33.06 (CH_2_), 31.10 (CH_2_), 28.44 (CH_2_), 24.69 (CH_2_), 22.04 (CH_2_), 13.92 (CH_3_, 7′-CH_3_).

HRMS (ESI) *m*/*z*, calculated for [M-H]^−^ C_19_H_25_O_6_^−^: 289.14453, found [M-H]^−^: 289.14493.

#### 3.2.3. *Cis*- and *Trans*-(±)-3,4-Dihydro-3-Nonyl-1-Oxo-1H-Isochromene-4-Carboxylic Acids (**3**)

Homophtalic anhydride (2.00 g, 12.0 mmol) reacted with decanal (1.72 g, 11.0 mmol) in the presence of 1.30 g (11.0 mmol) DMAP to give white crystals of **3** (2.00 g, 56% yield). After purification and separation, *cis* and *trans* isomers were acquired:

*cis*-**3**, m.p. = 133–135 °C (from CH_2_Cl_2_: petroleum ether, b.p. = 35–60 °C); R*_f_* = 0.42 (EtOAc: CH_2_Cl_2_ = 2:3); ^1^H-NMR (500 MHz, DMSO-_d6_): *δ* = 12.95 (1H, s, COOH), 7.95 (1H, d, *J* = 7.7 Hz, 8-CH), 7.64 (1H, t, *J* = 7.5 Hz, 6-CH), 7.51 (1H, t, *J* = 7.6 Hz, 7-CH), 7.44 (1H, d, *J* = 7.6 Hz, 5-CH), 4.67 (1H, td, ^3^*J*_3,1′_ = 6.8, ^3^*J*_3,4_ = 3.1 Hz, 3-CH), 3.99 (1H, d, ^3^*J*_3,4_ = 3.1 Hz, 4-CH), 1.82–1.71 (2H, m, 1′-CH_2_), 1.61–1.41 (2H, m, 2′-CH_2_), 1.41–1.11 (12H, m, 3′–8′-CH_2_), 0.86 (3H, t, ^3^*J*_8′,9′_ = 6.8 Hz, 9′-CH_3_). ^13^C-NMR (126 MHz, DMSO-_d6_): *δ* = 170.57 (C, C=O, COOH), 164.33 (C, 1C), 138.25 (C, 8aC), 133.63 (CH, 8C), 129.49 (CH, 6C), 128.49 (CH, 7C), 127.71 (CH, 5C), 125.25 (C, 4aC), 78.41 (CH, 3C), 46.69 (CH, 4C), 32.23 (CH_2_), 31.31 (CH_2_), 28.91 (CH_2_), 28.72 (CH_2_), 28.70 (CH_2_), 24.69 (CH_2_), 22.11 (CH_2_), 13.96 (CH_3_, 9′-CH_3_).

HRMS (ESI) *m*/*z*, calculated for [M-H]^−^ C_19_H_25_O_4_^−^: 317.17583, found [M-H]^−^: 317.17582.

*trans*-**3**, m.p = 110–111 °C (from CH_2_Cl_2_: petroleum ether, b.p. = 35–60 °C); R*_f_* = 0.39 (EtOAc: CH_2_Cl_2_ = 2:3); ^1^H-NMR (500 MHz, DMSO-_d6_): *δ* = 13.20 (1H, s, COOH), 7.95 (1H, d, *J* = 7.7 Hz, 8-CH), 7.68 (1H, t, *J* = 7.5 Hz, 6-CH), 7.50 (1H, t, *J* = 7.6 Hz, 7-CH), 7.43 (1H, d, *J* = 7.6 Hz, 5-CH), 4.92–4.83 (1H, m, 3-CH), 4.07 (1H, d, ^3^*J*_3,4_ = 4.1 Hz, 4-CH), 1.63–1.46 (2H, m, 1′-CH_2_), 1.47–1.30 (2H, m, 2′-CH_2_), 1.30–1.12 (12H, m, 3′–8′-CH_2_), 0.84 (3H, t, ^3^*J*_8′,9′_ = 6.8 Hz, 9′-CH_3_). ^13^C-NMR (126 MHz, DMSO-_d6_): *δ* = 172.24 (C, C=O, COOH), 163.54 (C, 1C), 136.94 (C, 8aC), 134.54 (CH, 8C), 129.60 (CH, 6C), 129.08 (CH, 7C), 128.81 (CH, 5C), 124.92 (C, 4aC), 79.53 (CH, 3C), 47.53 (CH, 4C), 33.54 (CH_2_), 31.72 (CH_2_), 29.29 (CH_2_), 29.26 (CH_2_) 29.12 (CH_2_), 28.94 (CH_2_), 25.15 (CH_2_), 22.54 (CH_2_), 14.39 (CH_3_, 9′-CH_3_).

HRMS (ESI) *m*/*z*, calculated for [M-H]^−^ C_19_H_25_O_4_^−^: 317.17583, found [M-H]^−^: 317.17582.

#### 3.2.4. *Cis*- and *Trans*-(±)-3-Decyl-3,4-Dihydro-1-Oxo-1H-Isochromene-4-Carboxylic Acids (**4**)

Homophtalic anhydride (2.00 g, 12.0 mmol) reacted with undecanal (1.90 g, 11.0 mmol) in the presence of 1.30 g (11.0 mmol) DMAP to give white crystals of **4** (2.20 g, 60% yield). After purification and separation, *cis* and *trans* isomers were acquired:

*cis*-**4**, m.p. = 129–131 °C (from CH_2_Cl_2_: petroleum ether, b.p. = 35–60 °C); R*_f_* = 0.43 (EtOAc: CH_2_Cl_2_ = 2:3); ^1^H-NMR (500 MHz, DMSO-_d6_): *δ* = 7.93 (1H, d, *J* = 7.9 Hz, 8-CH), 7.65 (1H, t, *J* = 7.5 Hz, 6-CH), 7.50 (1H, t, *J* = 7.6 Hz, 7-CH), 7.43 (1H, d, *J* = 7.5 Hz, 5-CH), 4.70–4.60 (1H, m, 3-CH), 3.97 (1H, d, ^3^*J*_3,4_ = 2.9 Hz, 4-CH), 1.81–1.69 (2H, m, 1′-CH_2_), 1.57–1.37 (2H, m, 2′-CH_2_), 1.35–1.18 (14H, m, 3′–9′-CH_2_), 0.86 (3H, t, ^3^*J*_9′,10′_ = 6.7 Hz, 10′-CH_3_). ^13^C-NMR (126 MHz, DMSO-_d6_): *δ* = 170.53 (C, C=O, COOH), 164.38 (C, 1C), 136.80 (C, 8aC), 133.58 (CH, 8C), 129.45 (CH, 6C), 128.39 (CH, 7C), 127.68 (CH, 5C), 125.26 (C, 4aC), 78.47 (CH, 3C), 46.87 (CH, 4C), 32.24 (CH_2_), 31.29 (CH_2_), 28.99 (CH_2_), 28.95 (CH_2_), 28.91 (CH_2_), 28.71 (CH_2_), 24.70 (CH_2_), 22.09 (CH_2_), 13.96 (CH_3_, 10′-CH_3_).

HRMS (ESI) *m*/*z*, calculated for [M-H]^−^ C_20_H_21_O_4_^−^: 289.14453, found [M-H]^−^: 289.14390.

*trans*-**3**, m.p = 110–111 °C (from CH_2_Cl_2_: petroleum ether, b.p. = 35–60 °C); R*_f_* = 0.39 (EtOAc: CH_2_Cl_2_ = 2:3); ^1^H-NMR (500 MHz, DMSO-_d6_): *δ* = 13.20 (1H, s, COOH), 7.94 (1H, d, *J* = 7.7 Hz, 8-CH), 7.68 (1H, t, *J* = 7.5 Hz, 6-CH), 7.50 (1H, t, *J* = 7.6 Hz, 7-CH), 7.43 (1H, d, *J* = 7.6 Hz, 5-CH), 4.92–4.83 (1H, m, 3-CH), 4.07 (1H, d, ^3^*J*_3,4_ = 4.1 Hz, 4-CH), 1.63–1.46 (2H, m, 1′-CH_2_), 1.47–1.30 (2H, m, 2′-CH_2_), 1.30–1.12 (12H, m, 3′–9′-CH_2_), 0.84 (3H, t, ^3^*J*_8′,9′_ = 6.8 Hz, 10′-CH_3_). ^013^C-NMR (126 MHz, DMSO-_d6_): *δ* = 172.25 (C, C=O, COOH), 163.54 (C, 1C), 136.94 (C, 8aC), 134.55 (CH, 8C), 129.60 (CH, 6C), 129.09 (CH, 7C), 128.82 (CH, 5C), 124.92 (C, 4aC), 79.53 (CH, 3C), 47.52 (CH, 4C), 33.54 (CH_2_), 31.74 (CH_2_), 29.42 (CH_2_), 29.34 (CH_2_), 29.25 (CH_2_), 29.14 (CH_2_), 28.94 (CH_2_), 25.15 (CH_2_), 22.55 (CH_2_) 14.40 (CH_3_, 10′-CH_3_).

HRMS (ESI) *m*/*z*, calculated for [M-H]^−^ C_19_H_25_O_6_^−^: 317.17583, found [M-H]^−^: 317.17582.

#### 3.2.5. *Cis*- and *Trans*-(±)-3-Propyl-3,4-Dihydro-6,7-Dimethoxy-1-Oxo-1H-Isochromene-4-Caboxylic Acids (**5**)

6,7-Dimethoxyhomophtalic anhydride (2.00 g, 9.00 mmol) reacted with butanal (0.59 g, 8.18 mmol) in the presence of 1.00 g (8.18 mmol) DMAP to give white crystals of **5** (1.32 g, 56% yield). After purification, two mixtures—**M1-5** and **M2-5**—were acquired with percentages of the *cis* isomer of 90% and 30%, respectively:

^1^H-NMR (500 MHz, DMSO-_d6_):
*cis* diastereomer: *δ* = 4.68–4.58 (1H, m, 3-CH), 3.87–3.78 (1H, m, 4-CH—the signal overlaps with the signals of 6-OCH_3_ and 7-OCH_3_).
*trans* diastereomer: *δ* = 4.91–4.85 (1H, m, 3-CH), 3.93 (1H, d, ^3^*J*_3,4_ = 3.1 Hz, 4-CH)
other signals for both diastereomers: *δ* = 12.88 (1H, s, COOH), 7.40 (1H, s, 8-CH), 7.02 (1H, s, 5-CH), 3.87–3.78 (6H, m, 6-OCH_3_, 7-OCH_3_), 1.81–1.34 (4H, m, 1′-CH_2_, 2′-CH_2_), 0.95 (3H, t, ^3^*J*_2′,3′_ = 7.4 Hz, 3′-CH_3_).

HRMS (ESI) *m*/*z*, calculated for [M-H]^−^ C_15_H_17_O_6_^−^: 293.10306, found [M-H]^−^: 293.10158.

#### 3.2.6. *Cis*- and *Trans*-(±)-3-Heptyl-3,4-Dihydro-6,7-Dimethoxy-1-Oxo-1H-Isochromene-4-Carboxylic Acids (**6**)

6,7-Dimethoxyhomophtalic anhydride (2.00 g, 9.00 mmol) reacted with octanal (1.05 g, 8.18 mmol) in the presence of 1.00 g (8.18 mmol) DMAP to give white crystals of **6** (2.63 g, 92% yield). After purification and separation, *cis* and *trans* isomers were acquired:

*cis*-**6**, m.p. = 132–134 °C (from CH_2_Cl_2_: petroleum ether, b.p. = 35–60 °C); R*_f_* = 0.37 (EtOAc: CH_2_Cl_2_ = 2:3); ^1^H-NMR (500 MHz, DMSO-_d6_): *δ* = 12.86 (1H, s, COOH), 7.39 (1H, s, 8-CH), 7.02 (1H, s, 5-CH), 4.65–4.58 (1H, td, ^3^*J*_3,1′_ = 7.0, ^3^*J*_3,4_ = 3.3 Hz, 3-CH), 3.86 (1H, d, ^3^*J*_3,4_ = 3.2 Hz, 4-CH), 3.84 (3H, s, 7-OCH_3_), 3.81 (3H, s, 6-OCH_3_), 1.81–1.68 (2H, m, 1′-CH_2_), 1.55–1.37 (2H, m, 2′-CH_2_), 1.37–1.20 (8H, m, 3′–6′-CH_2_), 0.87 (3H, t, ^3^*J*_6′,7′_ = 6.8 Hz, 7′-CH_3_). ^13^C-NMR (126 MHz, DMSO-_d6_): *δ* = 170.69 (C, C=O, COOH), 164.26 (C, 1C), 153.10 (C, 6C), 148.59 (C, 7C), 132.52 (C, 4aC), 117.19 (C, 8aC), 111.24 (CH, 8C), 110.14 (CH, 5C), 78.41 (CH, 3C), 55.97 (CH_3_, 6-OCH_3_), 55.68 (CH_3_, 7-OCH_3_), 46.41 (CH, 4C), 32.27 (CH_2_), 31.16 (CH_2_), 28.69 (CH_2_), 28.57 (CH_2_), 24.69 (CH_2_), 22.08 (CH_2_), 13.96 (7′-CH_3_).

HRMS (ESI) *m*/*z*, calculated for [M-H]^−^ C_19_H_25_O_6_^−^: 349.16566, found [M-H]^−^: 349.16430.

*trans*-**6**, m.p = 134–136 °C (from CH_2_Cl_2_: petroleum ether, b.p. = 35–60 °C); R*_f_* = 0.33 (EtOAc: CH_2_Cl_2_ = 2:3); ^1^H-NMR (500 MHz, DMSO-_d6_): *δ* = 13.10 (1H, s, COOH), 7.37 (1H, s, 8-CH), 6.99 (1H, s, 5-CH), 4.88–4.81 (1H, ddd, ^3^*J*_3,1′_ = 8.5, ^3^*J*_3,1′_ = 5.2, ^3^*J*_3,4_ = 3.3 Hz, 3-CH), 3.93 (1H, d, ^3^*J*_3,4_ = 3.3 Hz, 4-CH), 3.84 (3H, s, 7-OCH_3_), 3.81 (3H, s, 6-OCH_3_), 1.64–1.46 (2H, m, 1′-CH_2_), 1.45–1.30 (2H, m, 2′-CH_2_), 1.30–1.15 (8H, m, 3′–6′-CH_2_), 0.84 (3H, t, ^3^*J*_6′,7′_ = 6.9 Hz, 7′-CH_3_). ^13^C-NMR (126 MHz, DMSO-_d6_): *δ* = 172.49 (C, C=O, COOH), 163.30 (C, 1C), 153.88 (C, 6C), 148.94 (C, 7C), 130.92 (C, 4aC), 116.98 (C, 8aC), 111.66 (CH, 8C), 111.33 (CH, 5C), 79.56 (CH, 3C), 56.34 (CH_3_, 6-OCH_3_), 56.10 (CH_3_, 7-OCH_3_), 47.01 (CH, 4C), 33.60 (CH_2_), 31.58 (CH_2_), 28.94 (CH_2_), 28.91 (CH_2_), 25.32 (CH_2_), 22.50 (CH_2_), 14.39 (CH_3_, 7′-CH_3_).

HRMS (ESI) *m*/*z*, calculated for [M-H]^−^ C_19_H_25_O_6_^−^: 349.16566, found [M-H]^−^: 349.16366.

#### 3.2.7. *Cis*- and *Trans*-(±)-3,4-Dihydro-6,7-Dimethoxy-3-Nonyl-1-Oxo-1H-Isochromene-4-Carboxylic Acids (**7**)

6,7-dimethoxyhomophtalic anhydride (1.29 g, 5.8 mmol) reacted with decanal (0.83 g, 5.3 mmol) in the presence of 0.646 g (5.31 mmol) DMAP to give white crystals of **7** (1.71 g, 85% yield). After purification and separation, *cis* and *trans* isomers were acquired:

*cis*-**7**, m.p. = 137–139 °C (from CH_2_Cl_2_: petroleum ether, b.p. = 35–60 °C); R*_f_* = 0.39 (EtOAc: CH_2_Cl_2_ = 2:3); ^1^H-NMR (500 MHz, DMSO-_d6_): *δ* = 12.85 (1H, s, COOH), 7.39 (1H, s, 8-CH), 7.02 (1H, s, 5-CH), 4.66–4.56 (1H, td, ^3^*J*_3,1′_ = 6.9, ^3^*J*_3,4_ = 3.3 Hz, 3-CH), 3.85 (1H, d, ^3^*J*_3,4_ = 3.2 Hz, 4-CH), 3.84 (3H, s, 7-OCH_3_), 3.81 (3H, s, 6-OCH_3_), 1.81–1.67 (2H, m, 1′-CH_2_), 1.56–1.38 (2H, m, 2′-CH_2_), 1.38–1.19 (12H, m, 3′–8′-CH_2_), 0.86 (3H, t, ^3^*J*_8′,9′_ = 6.9 Hz, 9′-CH_3_). ^13^C-NMR (126 MHz, DMSO-_d6_): 171.15 (C, C=O, COOH), 164.72 (C, 1C), 153.57 (C, 6C), 149.06 (C, 7C), 132.98 (C, 4aC), 117.65 (C, 8aC), 111.70 (CH, 8C), 110.60 (CH, 5C), 78.87 (CH, 3C), 56.42 (CH_3_, 6-OCH_3_), 56.13 (CH_3_, 7-OCH_3_), 46.88 (CH, 4C), 32.74 (CH_2_), 31.77 (CH_2_), 29.40 (CH_2_), 29.37 (CH_2_), 29.20 (CH_2_), 29.16 (CH_2_), 25.16 (CH_2_), 22.57 (CH_2_), 14.43 (CH_3_, 9′-CH_3_).

HRMS (ESI) *m*/*z*, calculated for [M-H]^−^ C_21_H_29_O_6_^−^: 377.19696, found [M-H]^−^: 377.19537;

*trans*-**7**, m.p. = 140–143 °C (from CH_2_Cl_2_: petroleum ether, 35–60 °C); R*_f_* = 0.35 (EtOAc: CH_2_Cl_2_ = 2:3); ^1^H-NMR (500 MHz, DMSO-_d6_): *δ* = 13.05 (1H, s, COOH), 7.38 (1H, s, 8-CH), 6.99 (1H, s, 5-CH), 4.89–4.80 (1H, ddd, ^3^*J*_3,1′_ = 8.5, ^3^*J*_3,1′_ = 5.2, ^3^*J*_3,4_ = 3.3 Hz, 3-CH), 3.92 (1H, d, ^3^*J*_3,4_ = 3.3 Hz, 4-CH), 3.84 (3H, s, 7-OCH_3_), 3.81 (3H, s, 6-OCH_3_), 1.64–1.46 (2H, m, 1′-CH_2_), 1.44–1.30 (2H, m, 2′-CH_2_), 1.28–1.17 (12H, m, 3′–8′-CH_2_), 0.84 (3H, t, ^3^*J*_8′,9′_ = 6.9 Hz, 9′-CH_3_). ^13^C-NMR (126 MHz, DMSO-_d6_): 172.48 (C, C=O, COOH), 163.28 (C, 1C), 153.88 (C, 6C), 148.95 (C, 7C), 130.90 (C, 4aC), 116.98 (C, 8aC), 111.66 (CH, 8C), 111.32 (CH, 5C), 79.55 (CH, 3C), 56.33 (CH_3_, 6-OCH_3_), 56.09 (CH_3_, 7-OCH_3_), 47.00 (CH, 4C), 33.60 (CH_2_), 31.72 (CH_2_), 29.30 (CH_2_), 29.28 (CH_2_), 29.12 (CH_2_), 28.95 (CH_2_), 25.30 (CH_2_), 22.54 (CH_2_), 14.39 (CH_3_, 9′-CH_3_).

HRMS (ESI) *m*/*z*, calculated for [M-H]^−^ C_21_H_29_O_6_^−^: 377.19696, found [M-H]^−^: 377.19482.

#### 3.2.8. *Cis*- and *Trans*-(±)-3-Decyl-3,4-Dihydro-6,7-Dimethoxy-1-Oxo-1H-Isochromene-4-Carboxylic Acids (**8**)

6,7-dimethoxyhomophtalic anhydride (0.611 g, 2.80 mmol) reacted with undecanal (0.426 g, 2.50 mmol) in the presence of 0.306 g (2.50 mmol) DMAP to give white crystals of **8** (0.76 g, 77% yield). After purification and separation, *cis* and *trans* isomers were acquired:

*cis*-**8**, m.p. = 143–145 °C (from CH_2_Cl_2_: petroleum ether, b.p. = 35–60 °C); R*_f_* = 0.41 (EtOAc: CH_2_Cl_2_ = 2:3); ^1^H-NMR (500 MHz, DMSO-_d6_): *δ* = 12.86 (1H, s, COOH), 7.38 (1H, s, 8-CH), 7.02 (1H, s, 5-CH), 4.66–4.55 (1H, td, ^3^*J*_3,1′_ = 7.0, ^3^*J*_3,4_ = 3.3 Hz, 3-CH), 3.85 (1H, d, *J* = 3.2 Hz, 4-CH), 3.85 (3H, s, 7-OCH_3_), 3.82 (3H, s, 6-OCH_3_), 1.80–1.69 (2H, m, 1′-CH_2_), 1.56–1.38 (2H, m, 2′-CH_2_), 1.37–1.17 (14H, m, 3′–9′-CH_2_), 0.86 (3H, t, ^3^*J*_9′,10′_ = 6.9 Hz, 10′-CH_3_). ^13^C-NMR (126 MHz, DMSO-_d6_): 171.15 (C, C=O, COOH), 164.71 (C, 1C), 153.57 (C, 6C), 149.06 (C, 7C), 132.97 (C, 4aC), 117.65 (C, 8aC), 111.70 (CH, 8C), 110.59 (CH, 5C), 78.87 (CH, 3C), 56.42 (CH_3_, 6-OCH_3_), 56.13 (CH_3_, 7-OCH_3_), 46.88 (CH, 4C), 32.74 (CH_2_), 31.77 (CH_2_), 29.47 (CH_2_), 29.42 (CH_2_), 29.40 (CH_2_), 29.20 (CH_2_), 25.17 (CH_2_), 22.57 (CH_2_), 14.42 (CH_3_, 10′-CH_3_).

HRMS (ESI) *m*/*z*, calculated for [M-H]^−^ C_22_H_31_O_6_^−^: 391.21261, found [M-H]^−^: 391.21064.

*trans*-**8**, m.p. = 148–150 °C (from CH_2_Cl_2_: petroleum ether, b.p. = 35–60 °C); R*_f_* = 0.38 (EtOAc: CH_2_Cl_2_ = 2:3); ^1^H-NMR (500 MHz, DMSO-_d6_): *δ* = 13.06 (1H, s, COOH), 7.37 (1H, s, 8-CH), 6.99 (1H, s, 5-CH), 4.89–4.79 (1H, ddd, ^3^*J*_3,1′_ = 8.5, ^3^*J*_3,1′_ = 5.2, ^3^*J*_3,4_ = 3.3 Hz, 3-CH), 3.92 (1H, d, *J* = 3.3 Hz, 4-CH), 3.84 (3H, s, 7-OCH_3_), 3.81 (3H, s, 6-OCH_3_), 1.63–1.44 (2H, m, 1′-CH_2_), 1.44–1.30 (2H, m, 2′-CH_2_), 1.30–1.14 (14H, m, 3′–9′-CH_2_), 0.84 (3H, t, ^3^*J*_9′,10′_ = 6.9 Hz, 10′-CH_3_). ^13^C-NMR (126 MHz, DMSO-_d6_): 172.48 (C, C=O, COOH), 163.28 (C, 1C), 153.88 (C, 6C), 148.95 (C, 7C), 130.90 (C, 4aC), 116.98 (C, 8aC), 111.66 (CH, 8C), 111.32 (CH, 5C), 79.55 (CH, 3C), 56.33 (CH_3_, 6-OCH_3_), 56.09 (CH_3_, 7-OCH_3_), 47.00 (CH, 4C), 33.60 (CH_2_), 31.74 (CH_2_), 29.42 (CH_2_), 29.35 (CH_2_), 29.28 (CH_2_), 29.14 (CH_2_), 28.95 (CH_2_), 25.31 (CH_2_), 22.55 (CH_2_), 14.40 (CH_3_, 10′-CH_3_).

HRMS (ESI) *m*/*z*, calculated for [M-H]^−^ C_22_H_31_O_6_^−^: 391.21261, found [M-H]^−^: 391.21098.

#### 3.2.9. *Cis*- and *Trans*-(±)-3,4-Dihydro-6,7-Dimethoxy-1-Oxo-3-Phenyl-1H-Isochromene-4-Carboxylic Acids (**9**)

6,7-dimethoxyhomophtalic anhydride (2.00 g, 9.00 mmol) reacted with benzaldehyde (0.832 g, 7.84 mmol) in the presence of 0.958 g (7.84 mmol) DMAP to give white crystals of **9** (2.41 g, 92% yield). After purification and separation, *cis* and *trans* isomers were acquired:

*cis*-**9**, m.p. = 191–193 °C (from CH_2_Cl_2_: petroleum ether, b.p. = 35–60 °C); R*_f_* = 0.42 (EtOAc: CH_2_Cl_2_ = 2:3); ^1^H-NMR (500 MHz, DMSO-_d6_): *δ* = 12.58 (1H, s, COOH), 7.53–7.47 (3H, m, 8-CH, 2′-CH, 6′-CH), 7.43 (2H, t, *J* = 7.5, 3′-CH, 5′-CH), 7.39–7.34 (1H, m, 4′-CH), 7.06 (1H, s, 5-CH), 5.91 (1H, d, ^3^*J*_3,4_ = 3.6 Hz, 3-CH), 4.17 (1H, d, ^3^*J*_3,4_ = 3.6 Hz, 4-CH), 3.87 (3H, s, OCH_3_), 3.85 (3H, s, OCH_3_). ^13^C-NMR (126 MHz, DMSO-_d6_): 170.12 (C, C=O, COOH), 164.06 (C, 1C), 153.34 (C, 6C), 148.82 (C, 7C), 137.23 (C, 1′C), 132.05 (C, 4aC), 128.24 (CH, 2′C, 6′C), 125.79 (CH, 3′C, 5′C), 117.00 (C, 8aC), 111.44 (CH, 8C), 110.01 (CH, 5C), 78.72 (CH, 3C), 56.05 (CH_3_, OCH_3_), 55.74 (CH_3_, OCH_3_), 49.11 (CH, 4C).

HRMS (ESI) *m*/*z*, calculated for [M-H]^−^ C_18_H_15_O_6_^−^: 327.08741, found [M-H]^−^: 327.08598.

*trans*-**9**, m.p. = 208–210 °C (from CH_2_Cl_2_: petroleum ether, b.p. = 35–60 °C); R*_f_* = 0.39 (EtOAc: CH_2_Cl_2_ = 2:3); ^1^H-NMR (500 MHz, DMSO-_d6_): *δ* = 13.23 (1H, s, COOH), 7.40 (1H, s, 8-CH), 7.38–7.28 (5H, m, 2′–6′-CH), 6.93 (1H, s, 5-CH), 5.99 (1H, d, ^3^*J*_3,4_ = 5.0 Hz, 3-CH), 4.49 (1H, d, ^3^*J*_3,4_ = 5.1 Hz, 4-CH), 3.81 (3H, s, OCH_3_), 3.81 (3H, s, OCH_3_). ^13^C-NMR (126 MHz, DMSO-_d6_): 171.45 (C, C=O, COOH), 163.22 (C, 1C), 153.64 (C, 6C), 148.60 (C, 7C), 138.04 (C, 1′C), 130.55 (C, 4aC), 128.54 (CH, 2′C, 6′C), 128.34 (CH, 4′C), 126.47 (CH, 3′C, 5′C), 116.63 (C, 8aC), 110.88 (CH, 8C), 110.31 (CH, 5C), 79.62 (CH, 3C), 55.87 (CH_3_, OCH_3_), 55.65 (CH_3_, OCH_3_), 47.94 (CH, 4C).

HRMS (ESI) *m*/*z*, calculated for [M-H]^−^ C_18_H_15_O_6_^−^: 327.08741, found [M-H]^−^: 327.08581.

#### 3.2.10. *Cis*- and *Trans*-(±)-3,4-Dihydro-3-(2,3-Dimethoxyphenyl)-6,7-Dimethoxy-1-Oxo-1H-Isochromene-4-Carboxylic Acids (**10**)

6,7-dimethoxyhomophtalic anhydride (2.00 g, 9.00 mmol) reacted with 2,3-dimethoxybenzaldehyde (1.302 g, 7.84 mmol) in the presence of 0.958 g (7.84 mmol) DMAP to give white crystals of **10** (2.81 g, 91% yield). After purification and separation, *cis* and *trans* isomers were acquired:

*cis*-**10**, m.p. = 215–217 °C (from CH_2_Cl_2_: petroleum ether, b.p. = 35–60 °C); R*_f_* = 0.41 (EtOAc: CH_2_Cl_2_ = 2:3); ^1^H-NMR (500 MHz, DMSO-_d6_): *δ* = 12.57 (1H, s, COOH), 7.48 (1H, s, 8-CH), 7.16–7.05 (4H, m, 5-H, 4′-6′-CH), 6.01 (1H, d, ^3^*J*_3,4_ = 3.6, 3-CH), 4.05 (1H, d, ^3^*J*_3,4_ = 3.6 Hz, 4-CH), 3.91–3.79 (12H, m, 6-OCH_3_, 7-OCH_3_, 2′-OCH_3_, 3′-OCH_3_). ^13^C-NMR (126 MHz, DMSO-_d6_): 170.06 (C, C=O, COOH), 164.10 (C, 1C), 153.45 (C), 151.76 (C), 148.83 (C), 145.07 (C), 131.93 (C), 130.20 (C), 123.76 (CH), 118.16 (CH), 117.00 (C), 112.88 (CH), 111.44 (CH_3_, OCH_3_), 110.14 (CH), 74.80 (CH, 3C), 60.35 (CH_3_, OCH_3_), 56.08 (CH_3_, OCH_3_), 55.75 (CH_3_, OCH_3_), 55.71 (CH_3_, OCH_3_), 47.94 (CH, 4C).

HRMS (ESI) *m*/*z*, calculated for [M-H]^−^ C_20_H_19_O_8_^−^: 387.10854, found [M-H]^−^: 387.10692.

*trans*-**10**, m.p. = 208–210 °C (from CH_2_Cl_2_: petroleum ether, b.p. = 35–60 °C); R*_f_* = 0.36 (EtOAc: CH_2_Cl_2_ = 2:3); ^1^H-NMR (500 MHz, DMSO-_d6_): *δ* = 13.21 (1H, s, COOH), 7.44 (1H, s, 8-CH), 7.07–6.90 (2H, m, 4′-CH, 6′-CH), 6.94 (1H, s, 5-CH), 6.66 (1H, dd, *J* = 7.7, 1.4 Hz, 5′-CH), 6.18 (1H, d, ^3^*J*_3,4_ = 4.9 Hz, 3-CH), 4.39 (1H, d, ^3^*J*_3,4_ = 5.0 Hz, 4-CH), 3.90–3.71 (12H, m, 6-OCH_3_, 7-OCH_3_, 2′-OCH_3_, 3′-OCH_3_). ^13^C-NMR (126 MHz, DMSO-_d6_): 171.97 (C, C=O, COOH), 163.84 (C, 1C), 154.13 (C), 152.87 (C), 149.09 (C), 146.45 (C), 131.52 (C), 130.94 (C), 124.34 (CH), 118.98 (CH), 116.91 (C), 113.77 (CH), 111.31 (CH), 110.93 (CH), 75.98 (CH), 60.85 (CH_3_, OCH_3_), 56.34 (CH_3_, OCH_3_), 56.20 (CH_3_, OCH_3_), 56.14 (CH_3_, OCH_3_) 47.63 (CH, 4C).

HRMS (ESI) *m*/*z*, calculated for [M-H]^−^ C_18_H_15_O_6_^−^: 327.08741, found [M-H]^−^: 387.10689.

#### 3.2.11. *Cis*- and *Trans*-(±)-3,4-Dihydro-3-(2,5-Dimethoxyphenyl)-6,7-Dimethoxy-1-Oxo-1H-Isochromene-4-Carboxylic Acids (**11**)

6,7-dimethoxyhomophtalic anhydride (2.00 g, 9.00 mmol) reacted with 2,5-dimethoxybenzaldehyde (1.302 g, 7.84 mmol) in the presence of 0.958 g (7.84 mmol) DMAP to give white crystals of **11** (2.80 g, 90% yield). After purification and separation, *cis* isomer and a mixture of *cis* and *trans* isomers—**M-11** in a 60%/40% ratio were acquired:

*cis*-**11**, m.p. = 210–212 °C (from CH_2_Cl_2_: petroleum ether, b.p. = 35–60 °C); R*_f_* = 0.40 (EtOAc: CH_2_Cl_2_ = 2:3); ^1^H-NMR (500 MHz, DMSO-_d6_): *δ* = 12.57 (1H, s, COOH), 7.47 (1H, s, 8-CH), 7.11 (1H, s, 5-H), 7.05–6.99 (2H, m, 3′-CH, 6′-CH), 6.92 (1H, dd, ^3^*J*_3,4_ = 8.9, 3.2 Hz, 4′-CH), 5.95 (1H, d, ^3^*J*_3,4_ = 3.5 Hz, 3-CH), 4.11 (1H, d, ^3^*J*_3,4_ = 3.5 Hz, 4-CH), 3.87–3.73 (12H, m, 6-OCH_3_, 7-OCH_3_, 2′-OCH_3_, 5′-OCH_3_). ^13^C-NMR (126 MHz, DMSO-_d6_): 170.05 (C, C=O, COOH), 164.04 (C, 1C), 153.45 (C), 153.03 (C), 149.53 (C), 148.81 (C), 131.80 (C), 125.83 (C), 116.95 (C), 113.42 (CH), 112.67 (CH), 111.83 (CH), 111.45 (CH), 110.17 (CH), 74.38 (CH, 3C), 59.76 (CH_3_, OCH_3_), 56.08 (CH_3_, OCH_3_), 55.73 (CH_3_, OCH_3_), 55.43 (CH_3_, OCH_3_), 46.98 (CH, 4C).

HRMS (ESI) *m*/*z*, calculated for [M-H]^−^ C_20_H_19_O_8_^−^: 387.10854, found [M-H]^−^: 387.10685.

*trans* diastereomer: R*_f_* = 0.37 (EtOAc: CH_2_Cl_2_ = 2:3); ^1^H-NMR (500 MHz, DMSO-_d6_): *δ* = 6.17 (1H, d, ^3^*J*_3,4_ = 3.8 Hz, 3-CH), 4.36 (1H, d, ^3^*J*_3,4_ = 3.9 Hz, 4-CH).

HRMS (ESI) *m*/*z*, calculated for [M-H]^−^ C_20_H_19_O_8_^−^: 387.10854, found [M-H]^−^: 387.10677.

### 3.3. In Vitro Studies

To determine the effect of the synthesized compounds on CAT activity, a modified procedure for spectrophotometric determination of L-carnitine using Ellman’s reagent [47] was used. In all analyses, an enzyme isolated from pigeon breast muscle (Sigma Aldrich) was used in the form of an ammonium sulfate suspension with an activity of 71 U/mg protein. One unit of enzyme catalyzes the conversion of 1 μmol of L-carnitine and acetyl-CoA into acetylcarnitine and free CoA in 1 min. Phosphate buffer (0.5 M, pH = 7.6) is used to dilute the enzyme to obtain a stock solution with a concentration of 24 U/mL.

Ellman’s reagent was prepared immediately before each measurement by dissolving 25 mg DTNB in 5 mL 1 mM solution of Na_2_EDTA in phosphate buffer (0.5 M, pH = 7.6). Stock solutions of acetyl-CoA and L-carnitine were prepared in deionized water with concentrations of 348.0 μM and 303.4, respectively. An aqueous solution of tris (hydroxymethyl) aminomethane (TRIS) with pH = 7.6 and 1 M concentration was used as a buffer solution. Test compounds were dissolved and diluted in phosphate buffer (0.5 M, pH = 7.6) to the desired stock concentration. The working volume of the reaction is 300 μL and contains 50 μL of each of the six components (DTNB, TRIS, acetyl-CoA, CAT, L-carnitine and inhibitor) solutions with concentrations as described below. In the control samples, the inhibitor solution is replaced with phosphate buffer (0.5 M, pH = 7.6). The incubation time for all components without L-carnitine is 5 min at 37 °C. The reaction was started by adding L-carnitine, and its progress was monitored by reading the change in absorbance at 405 nm in kinetic mode. The concentrations of the components in the final volume were, c (DTNB) = 114 μM, c (TRIS) = 100 mM, c (acetyl-CoA) = 58 μM, c (CAT) = 4 U/mL and c (L-carnitine) = 50.56 μM. The time for reading the results was the first minute after starting the reaction.

To establish the mechanism of inhibition of the most active compounds, extensive kinetic studies were carried out, and the obtained data on the initial rate of the reaction, v_0_, were used to construct the *v*_0_ vs. [S] (Michaelis-Menten) and 1/*v*_0_ vs. [S] (Lainweaver-Burke) relationships. For this purpose, five solutions with different concentrations of the inhibitor and five solutions with different concentrations of L-carnitine were prepared. The concentrations of the remaining components in the final volume were c (DTNB) = 114 μM, c (TRIS) = 100 mM, c (acetyl-CoA) = 58 μM and c (CAT) = 4 U/mL, respectively. The time for reading the results is every six seconds for three minutes after the start of the reaction, which corresponds to the end of the linear interval. The initial velocity v_0_ was determined using the method proposed by Baici [48], by calculating the cut-off of the *v* vs. *t* (time) dependence for the linear interval of each of the reactions. Graphical dependencies are provided in the Supplementary Materials (Figures S1–S3).

## 4. Conclusions

We investigated 3-alkyl-3,4-dihydroisocoumarin-4-carboxylic acids as potential inhibitors of carnitine acetyltransferase (CAT) using a structure-based rational approach. Structure-activity relationship studies showed that these compounds bind more effectively to the enzyme’s active site than natural substrates or known inhibitors, indicating a potential impact on fatty acid β-oxidation. Eleven diastereomeric pairs were synthesized, purified and tested in vitro, demonstrating CAT inhibitory activity with IC_50_ values between 100 μM and 1 mM. Notably, some derivatives outperformed the reference inhibitor Meldonium (IC_50_ = 11.4 mM). The presence and length of a hydrophobic alkyl group at position 3 of the benzopyranone moiety were crucial for activity. Kinetic analyses identified the most potent compounds (*cis*-**8** and *trans*-**8**) as mixed inhibitors. Given their demonstrated inhibitory potential, the compounds studied herein are promising metabolic modulators for the treatment of inflammation, ischemic diseases, diabetes and certain types of cancer.

## Figures and Tables

**Table 1:** Coefficient of determination (R^2^), Akaike Information Criterion (AIC) and standard deviation of the residuals (S_y,x_) for the different types of enzyme inhibition based on kinetic measurement of CAT activity in the presence of the most active compounds, *cis*-**8** and *trans*-**8**.

Compd.	Type of Inhibition	α	R^2^	AIC	S_y,x_
*cis*-**8**	mixed	0.97	0.98027	−1562.764	2.987 × 10^−9^
	non-competitive	1	0.98027	−1565.380	2.947 × 10^−9^
	uncompetitive	-	0.97141	−1550.555	3.547 × 10^−9^
	competitive	-	0.93830	−1519.782	5.211 × 10^−9^
*trans*-**8**	mixed	0.21	0.97682	−1548.364	3.576 × 10^−9^
	uncompetitive	-	0.97551	−1548.795	3.626 × 10^−9^
	non-competitive	1	0.97122	−1542.332	3.931 × 10^−9^
	competitive	-	0.89542	−1490.723	7.493 × 10^−9^

## Data Availability

The original contributions presented in this study are included in the Supplementary Materials. Further inquiries can be directed to the corresponding author.

## Supplementary Materials

The following supporting information can be downloaded at: https://www.mdpi.com/article/10.3390/molecules30153159/s1, Figures S1–S3: Michaelis-Menten and Lineweaver-Burk plots for different types of inihibition; Figures S4–S59: ^1^H- and ^13^C-NMR spectra of all compounds described in the script.
