# Supplementary material for: Rational Design, Synthesis and In Vitro Activity of Diastereomeric Cis-/Trans-3-Substituted-3,4-Dihydroisocoumarin-4-Carboxylic Acids as Potential Carnitine Acetyltransferase Inhibitors
Source: Molecules. 2025 Jul 28;30(15):3159. doi: 10.3390/molecules30153159 (PMC12348882; doi:10.3390/molecules30153159)
Supplement: Supplementary file 1 [file molecules-30-03159-s001.zip › molecules-3772921-supplementary.pdf]

Supplementary Material

Rational Design, Synthesis and In Vitro activity of Diastereomeric *Cis-/Trans*-3-Substituted-3,4-Dihydroisocoumarin-4-Carboxylic Acids as Potential Carnitine Acetyltransferase inhibitors

Savina Stoyanova and Milen G. Bogdanov

Content:

Figure S1. The dependence of the reaction rate on time. Speed decreases linearly from 36 seconds to the end of the measurement - 180 seconds.

Figure S2. Graphs representing the dependence of the initial velocity (v_0) on the concentration of the substrate [S], according to the model of Michaelis-Menten – left, and Lineweaver-Burk – right, for reversible a) competitive, b) noncompetitive and c) uncompetitive inhibition in the presence of *cis*-8.

Figure S3. Graphs representing the dependence of the initial velocity (v_0) on the concentration of the substrate [S], according to the model of Michaelis-Menten – left, and Lineweaver-Burk – right, for reversible a) competitive, b) noncompetitive and c) uncompetitive inhibition in the presence of *trans*-8.

Figure S4. ^{1}H-NMR Spectrum of M1-1 in DMSO-d_6.

Figure S5. ^{1}H-NMR Spectrum of M2-1 in DMSO-d_6.

Figure S6. ^{1}H-NMR Spectrum of *cis*-2 in DMSO-d_6.

Figure S7. ^{13}C-NMR Spectrum of *cis*-2 in DMSO-d_6.

Figure S8. DEPT-135 NMR Spectrum of *cis*-2 in DMSO-d_6.

Figure S9. ^{1}H-NMR Spectrum *trans*-2 in DMSO-d_6.

Figure S10. ^{13}C-NMR Spectrum of *trans*-2 in DMSO-d_6.

Figure S11. DEPT-135 NMR Spectrum of *trans*-2 in DMSO-d_6.

Figure S12. ^{1}H-NMR Spectrum of *cis*-3 in DMSO-d_6.

Figure S13. ^{13}C-NMR Spectrum of *cis*-3 in DMSO-d_6.

Figure S14. DEPT-135 NMR Spectrum of *cis*-3 in DMSO-d_6.

Figure S15. ^{1}H-NMR Spectrum *trans*-3 in DMSO-d_6.

Figure S16. ^{13}C-NMR Spectrum of *trans*-3 in DMSO-d_6.

Figure S17. DEPT-135 NMR Spectrum of *trans*-3 in DMSO-d_6.

Figure S18. ^{1}H-NMR Spectrum of *cis*-4 in DMSO-d_6.

Figure S19. ^{13}C-NMR Spectrum of *cis*-4 in DMSO-d_6.

Figure S20. DEPT-135 NMR Spectrum of *cis*-4 in DMSO-d_6.

Figure S21. ^{1}H-NMR Spectrum *trans*-4 in DMSO-d$_6$.

Figure S22. ^{13}C-NMR Spectrum of *trans*-4 in DMSO-d$_6$.

Figure S23. DEPT-135 NMR Spectrum of *trans*-4 in DMSO-d$_6$.

Figure S24. ^{1}H-NMR Spectrum of M1-5 in DMSO-d$_6$.

Figure S25. ^{1}H-NMR Spectrum M2-5 in DMSO-d$_6$.

Figure S26. ^{1}H-NMR Spectrum of *cis*-6 in DMSO-d$_6$.

Figure S27. ^{13}C-NMR Spectrum of *cis*-6 in DMSO-d$_6$.

Figure S28. DEPT-135 NMR Spectrum of *cis*-6 in DMSO-d$_6$.

Figure S29. ^{1}H-NMR Spectrum *trans*-6 in DMSO-d$_6$.

Figure S30. ^{13}C-NMR Spectrum of *trans*-6 in DMSO-d$_6$.

Figure S31. DEPT-135 NMR Spectrum of *trans*-6 in DMSO-d$_6$.

Figure S32. ^{1}H-NMR Spectrum of *cis*-7 in DMSO-d$_6$.

Figure S33. ^{13}C-NMR Spectrum of *cis*-7 in DMSO-d$_6$.

Figure S34. DEPT-135 NMR Spectrum of *cis*-7 in DMSO-d$_6$.

Figure S35. ^{1}H-NMR Spectrum *trans*-7 in DMSO-d$_6$.

Figure S36. ^{13}C-NMR Spectrum of *trans*-7 in DMSO-d$_6$.

Figure S37. DEPT-135 NMR Spectrum of *trans*-7 in DMSO-d$_6$.

Figure S38. ^{1}H-NMR Spectrum of *cis*-8 in DMSO-d$_6$.

Figure S39. ^{13}C-NMR Spectrum of *cis*-8 in DMSO-d$_6$.

Figure S40. DEPT-135 NMR Spectrum of *cis*-8 in DMSO-d$_6$.

Figure S41. ^{1}H-NMR Spectrum *trans*-8 in DMSO-d$_6$.

Figure S42. ^{13}C-NMR Spectrum of *trans*-8 in DMSO-d$_6$.

Figure S43. DEPT-135 NMR Spectrum of *trans*-8 in DMSO-d$_6$.

Figure S44. ^{1}H-NMR Spectrum of *cis*-9 in DMSO-d$_6$.

Figure S45. ^{13}C-NMR Spectrum of *cis*-9 in DMSO-d$_6$.

Figure S46. DEPT-135 NMR Spectrum of *cis*-9 in DMSO-d$_6$.

Figure S47. ^{1}H-NMR Spectrum *trans*-9 in DMSO-d$_6$.

Figure S48. ^{13}C-NMR Spectrum of *trans*-9 in DMSO-d$_6$.

Figure S49. DEPT-135 NMR Spectrum of *trans*-9 in DMSO-d$_6$.

Figure S50. ^{1}H-NMR Spectrum of *cis*-10 in DMSO-d$_6$.

Figure S51. ^{13}C-NMR Spectrum of *cis*-10 in DMSO-d$_6$.

Figure S52. **DEPT-135 NMR Spectrum of *cis*-10 in DMSO-d_6.**

Figure S53. **^{1}H-NMR Spectrum *trans*-10 in DMSO-d_6.**

Figure S54. **^{13}C-NMR Spectrum of *trans*-10 in DMSO-d_6.**

Figure S55. **DEPT-135 NMR Spectrum of *trans*-10 in DMSO-d_6.**

Figure S56. **^{1}H-NMR Spectrum of *cis*-11 in DMSO-d_6.**

Figure S57. **^{13}C-NMR Spectrum of *cis*-11 in DMSO-d_6.**

Figure S58. **DEPT-135 NMR Spectrum of *cis*-11 in DMSO-d_6.**

Figure S59. **^{1}H-NMR Spectrum M-11 in DMSO-d_6.**

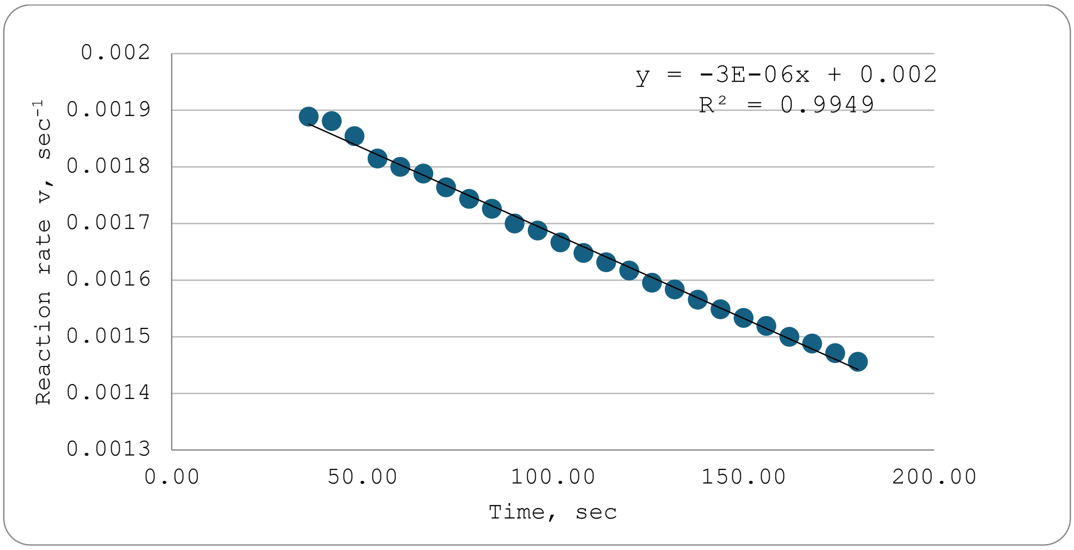

Figure S1. The dependence of the reaction rate on time (c(Inh) = 0, c(L-carnitine) = 359.53mM). Speed decreases linearly from 36 seconds to the end of the measurement - 180 seconds.

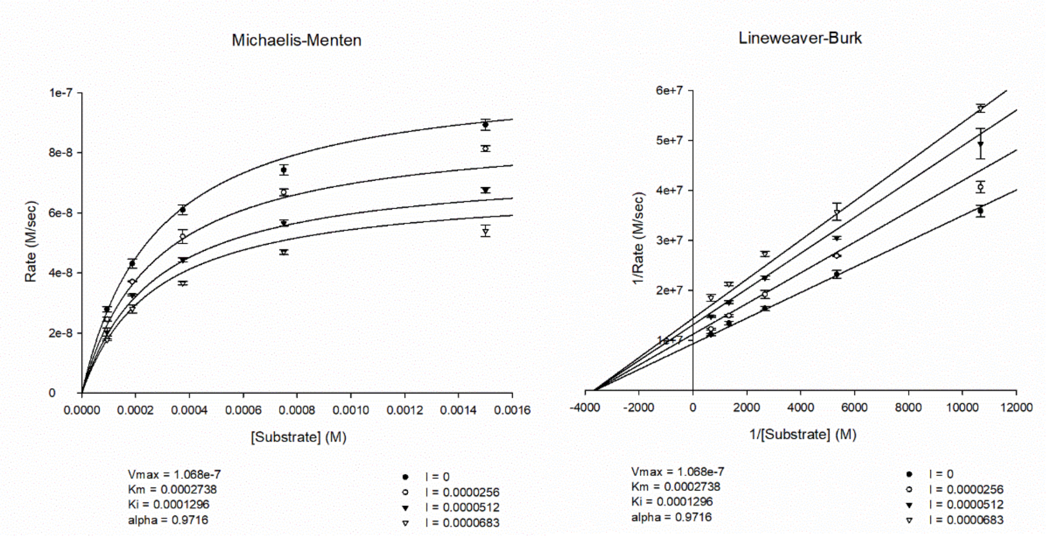
Michaelis-Menten
Rate (M/sec)
[Substrate] (M)
Vmax = 1.068e-7
Km = 0.0002738
Ki = 0.0001296
alpha = 0.9716
I = 0
I = 0.0000256
I = 0.0000512
I = 0.0000683
Lineweaver-Burk
1/Rate (M/sec)
1/[Substrate] (M)
Vmax = 1.068e-7
Km = 0.0002738
Ki = 0.0001296
alpha = 0.9716
I = 0
I = 0.0000256
I = 0.0000512
I = 0.0000683

a)

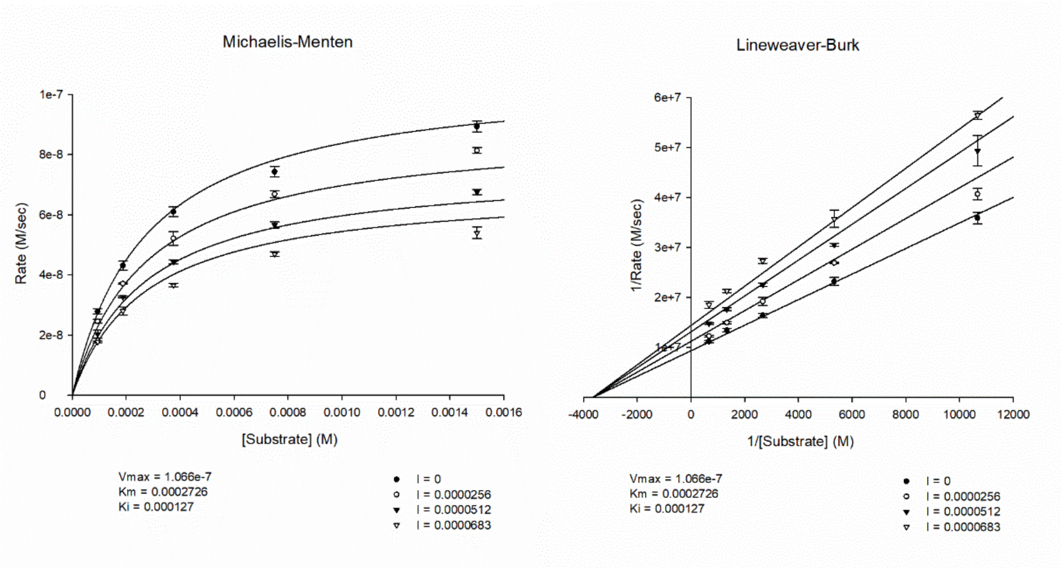
Michaelis-Menten
Rate (M/sec)
[Substrate] (M)
Vmax = 1.066e-7
Km = 0.0002726
Ki = 0.000127
I = 0
I = 0.0000256
I = 0.0000512
I = 0.0000683
Lineweaver-Burk
1/Rate (M/sec)
1/[Substrate] (M)
Vmax = 1.066e-7
Km = 0.0002726
Ki = 0.000127
I = 0
I = 0.0000256
I = 0.0000512
I = 0.0000683

b)

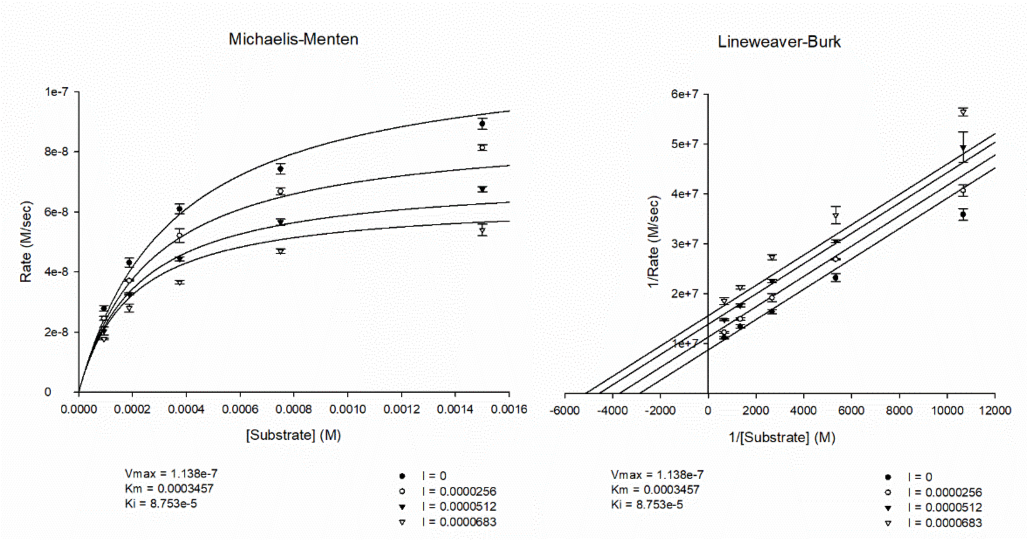

c)

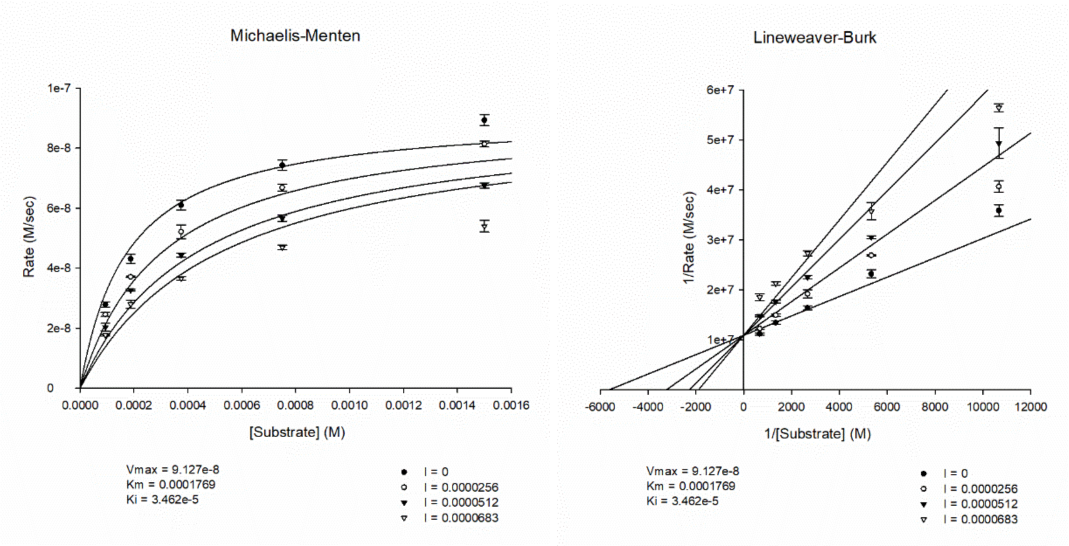

d)

Figure S2. Graphs representing the dependence of the initial velocity (v_0) on the concentration of the substrate [S], according to the model of Michaelis-Menten – left, and Lineweaver-Burk – right, for reversible a) mixed, b) non-competitive, c) uncompetitive and d) competitive inhibition. The concentration of the inhibitor - compound *cis*-8 are 0 µM, 25.6 µM, 51.2 µM and 68.3 µM, concentration of L-carnitine are 94 µM, 180 µM, 370 µM, 750 µM and 1500 µM.

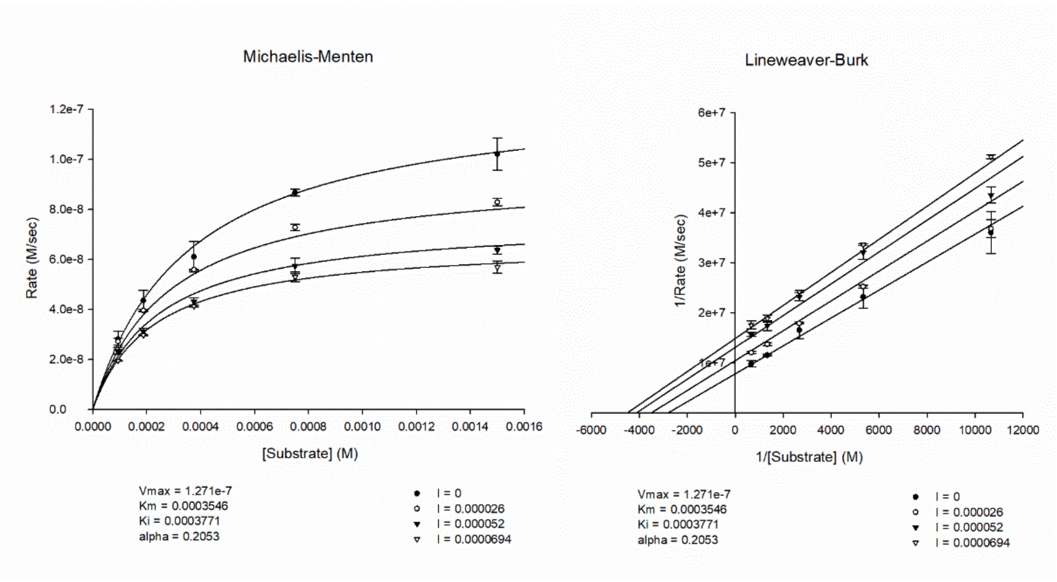
Michaelis-Menten
Rate (M/sec)
[Substrate] (M)
Vmax = 1.271e-7
Km = 0.0003546
Ki = 0.0003771
alpha = 0.2053
I = 0
I = 0.000026
I = 0.000052
I = 0.0000694
Lineweaver-Burk
1/Rate (M/sec)
1/[Substrate] (M)
Vmax = 1.271e-7
Km = 0.0003546
Ki = 0.0003771
alpha = 0.2053
I = 0
I = 0.000026
I = 0.000052
I = 0.0000694

a)

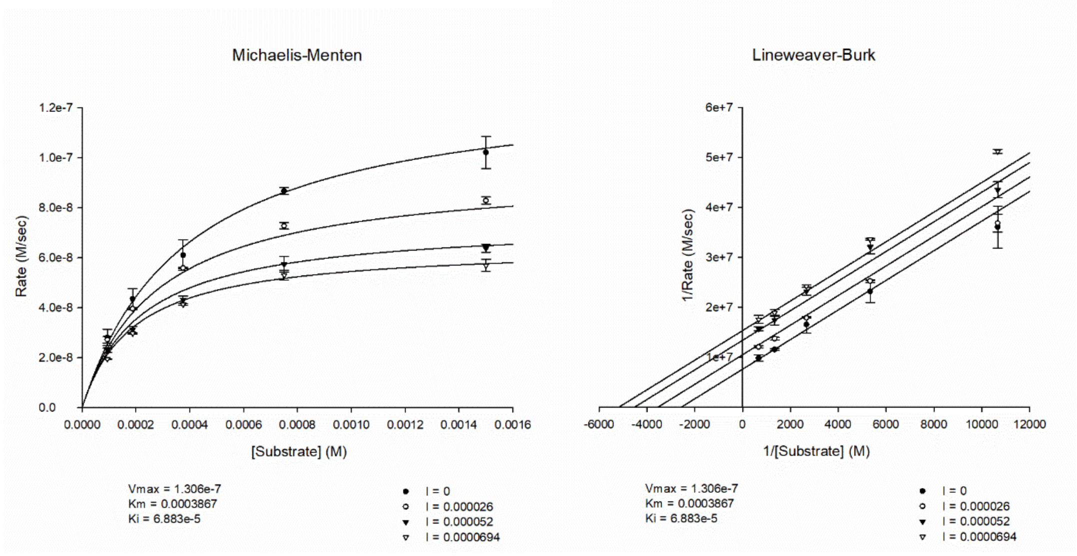
Michaelis-Menten
Rate (M/sec)
[Substrate] (M)
Vmax = 1.306e-7
Km = 0.0003867
Ki = 6.883e-5
I = 0
I = 0.000026
I = 0.000052
I = 0.0000694
Lineweaver-Burk
1/Rate (M/sec)
1/[Substrate] (M)
Vmax = 1.306e-7
Km = 0.0003867
Ki = 6.883e-5
I = 0
I = 0.000026
I = 0.000052
I = 0.0000694

b)

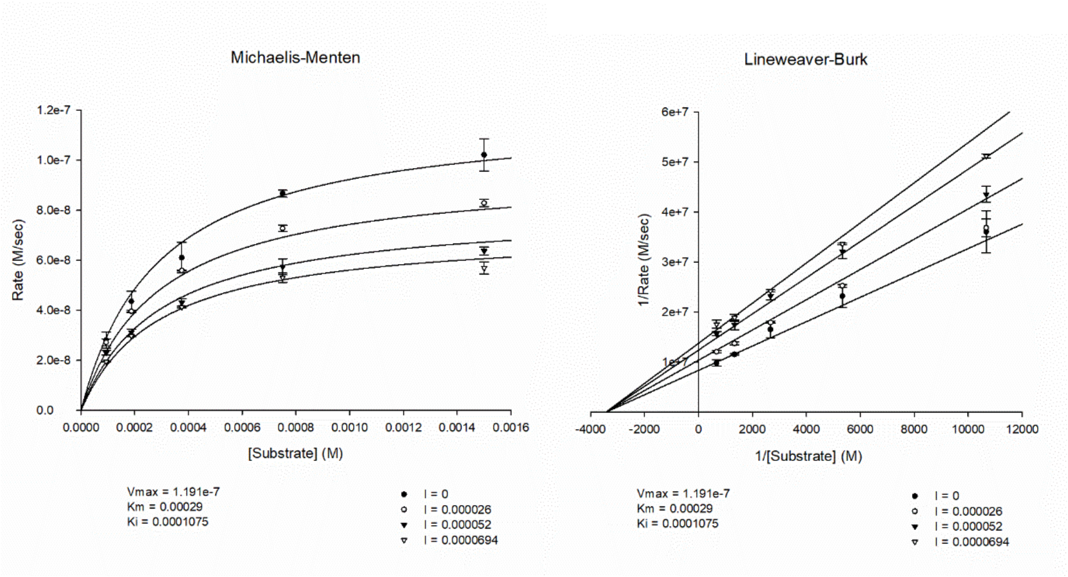

c)

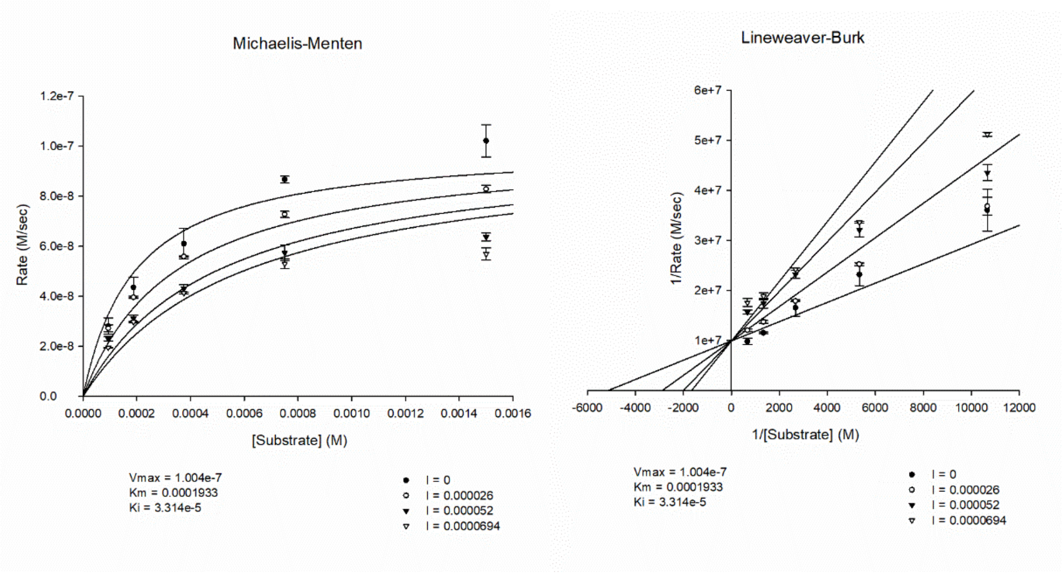

d)

Figure S3. Graphs representing the dependence of the initial velocity (v_0) on the concentration of the substrate [S], according to the model of Michaelis-Menten – left, and Lineweaver-Burk – right, for reversible a) mixed, b) uncompetitive, c) non-competitive and d) competitive inhibition. The concentration of the inhibitor - compound *trans*-8 are 0 µM, 26.0 µM, 52.0 and 69.4 µM, concentration of L-carnitine are 94 µM, 180 µM, 370 µM, 750 µM and 1500 µM.

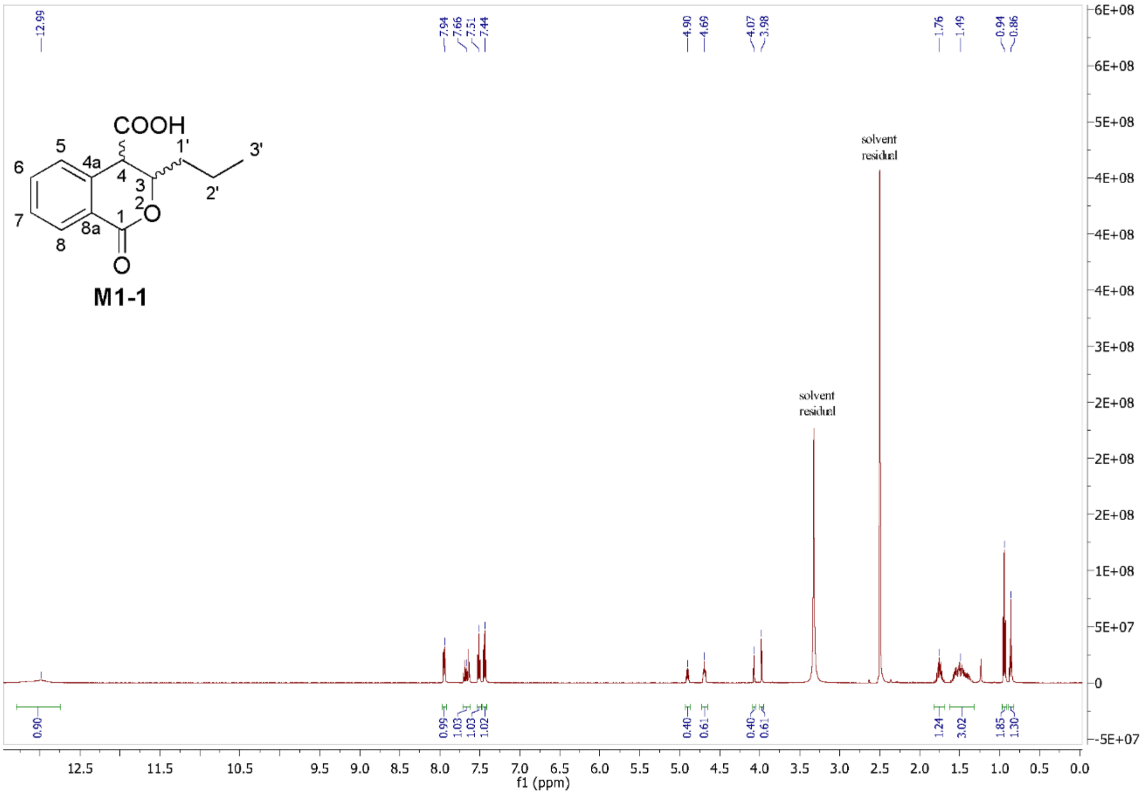

Figure S4. ^{1}H-NMR Spectrum of M1-1 DMSO-d6.

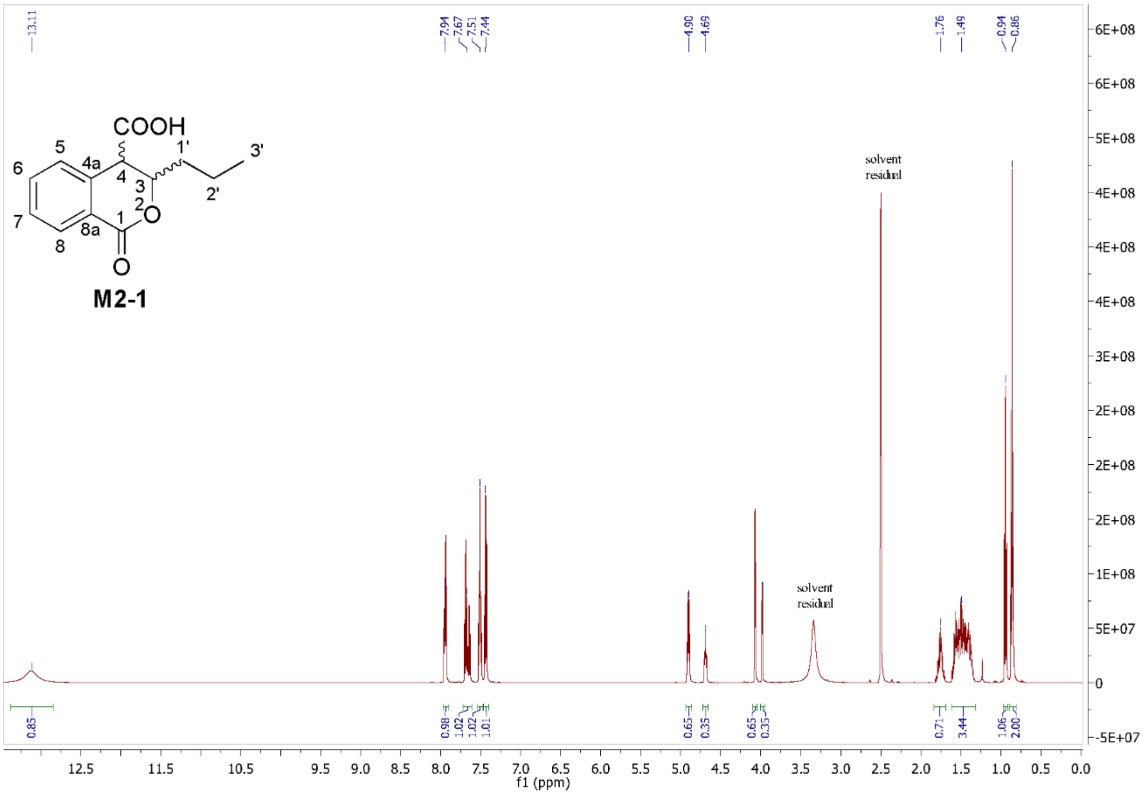

Figure S5. ^{1}H-NMR Spectrum of M2-1 DMSO-d6.

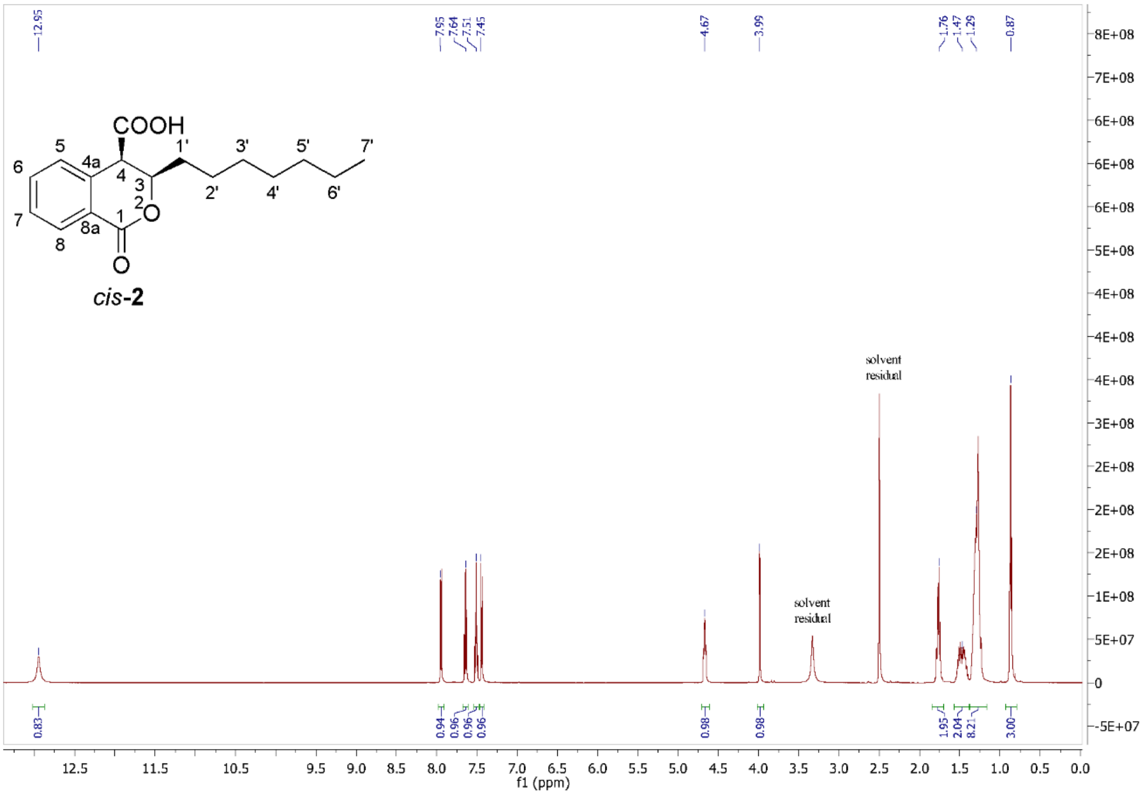

Figure S6. **^{1}H-NMR Spectrum of *cis*-2 DMSO-d6.**

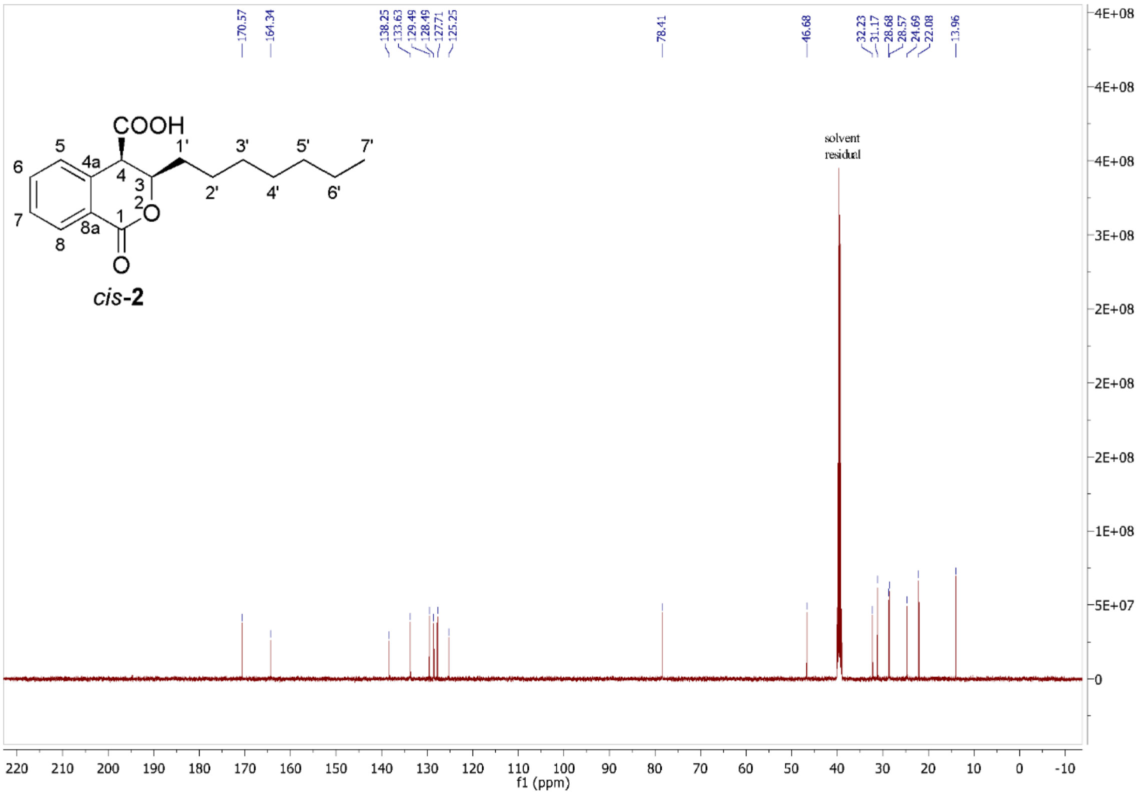

Figure S7. **^{13}C-NMR Spectrum of *cis*-2 in DMSO-d6.**

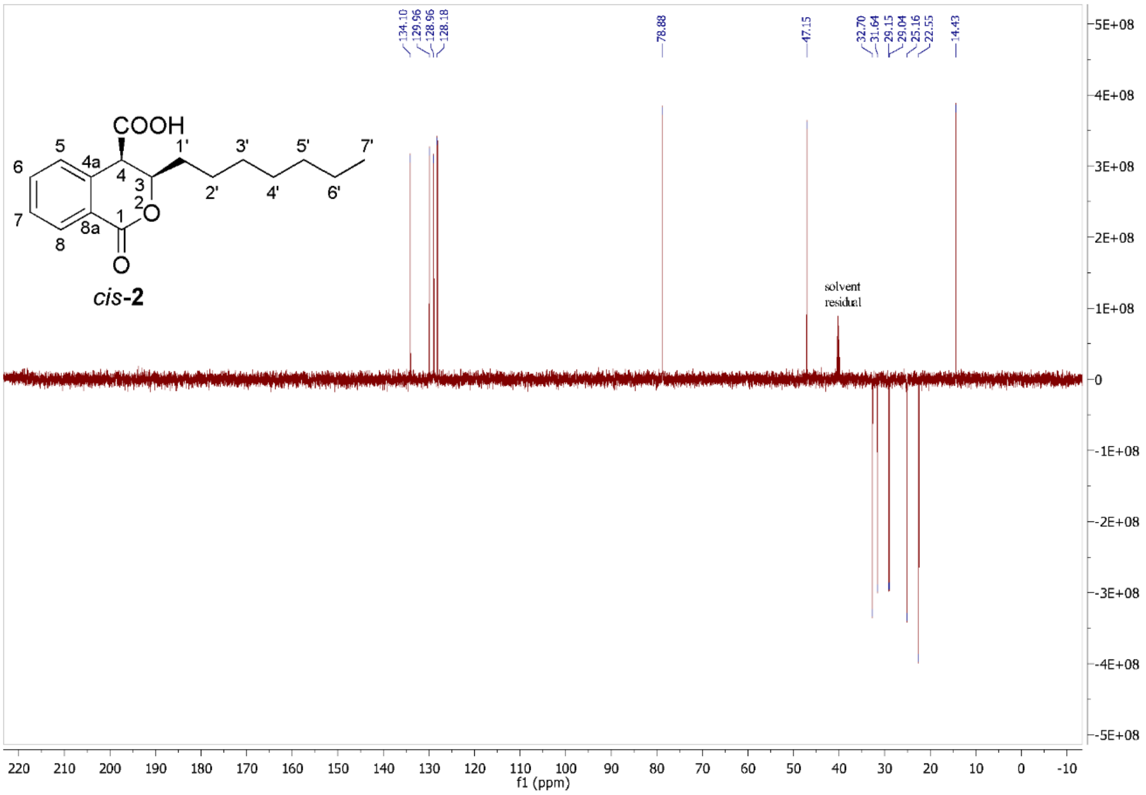

Figure S8. DEPT-135 NMR Spectrum of *cis*-2 in DMSO-d6.

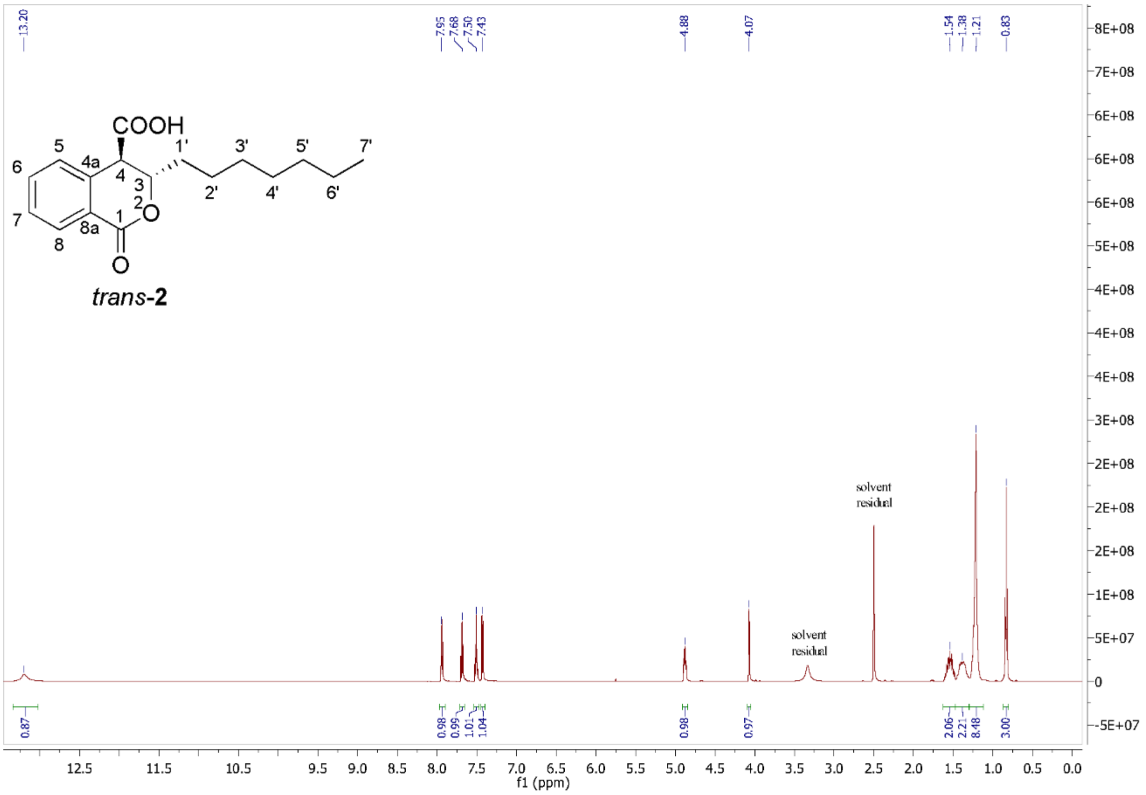

Figure S9. ¹H-NMR Spectrum of *trans*-2 DMSO-d6.

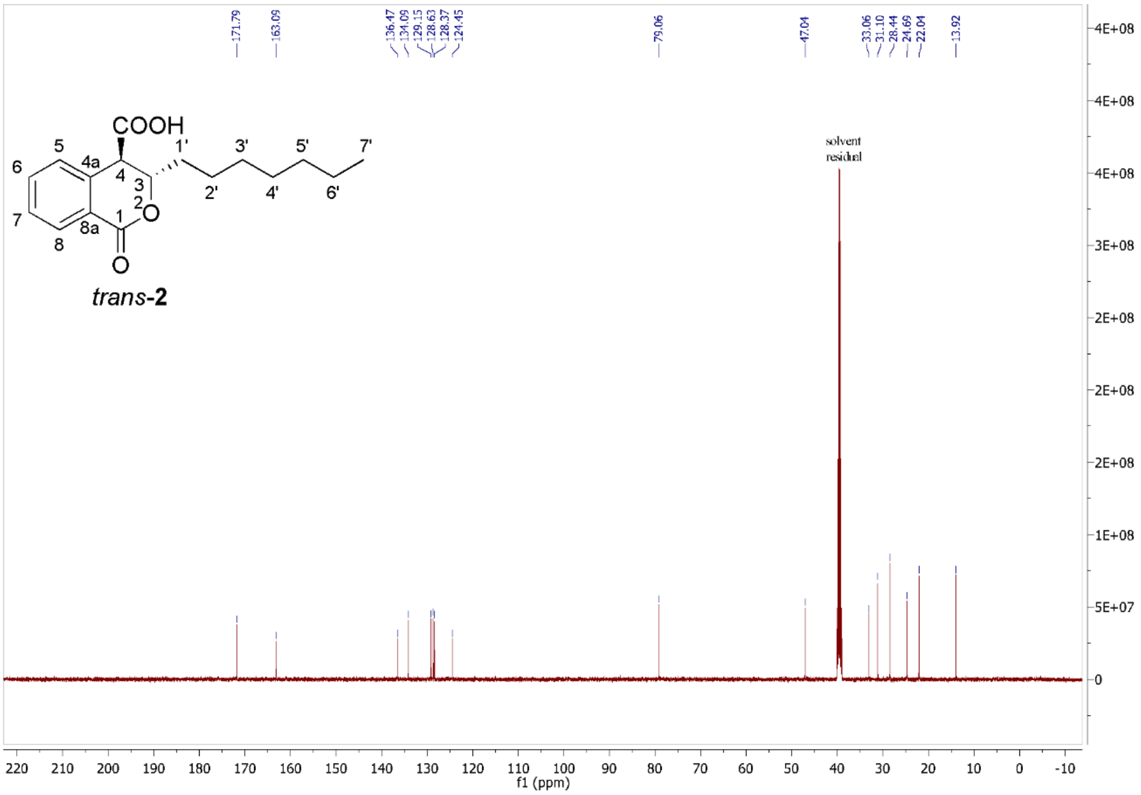

Figure S10. ^{13}C-NMR Spectrum of *trans*-2 in DMSO-d_6.

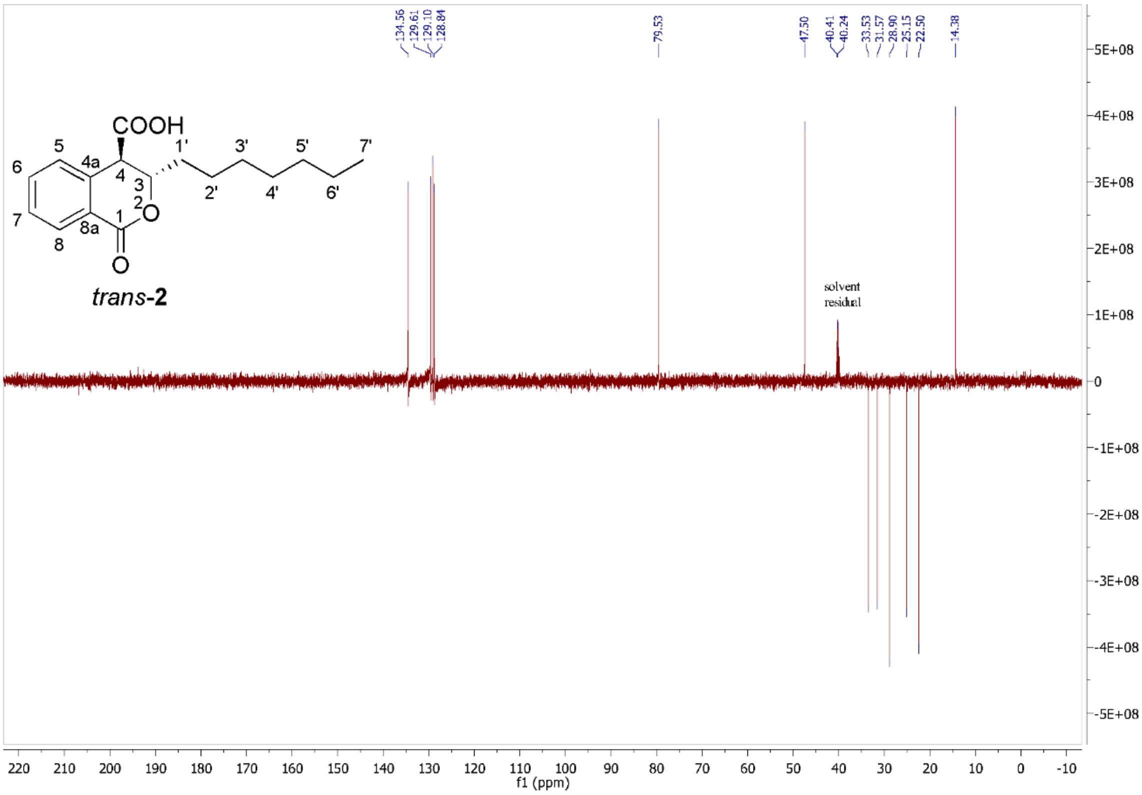

Figure S11. DEPT-135 NMR Spectrum of *trans*-2 in DMSO-d_6.

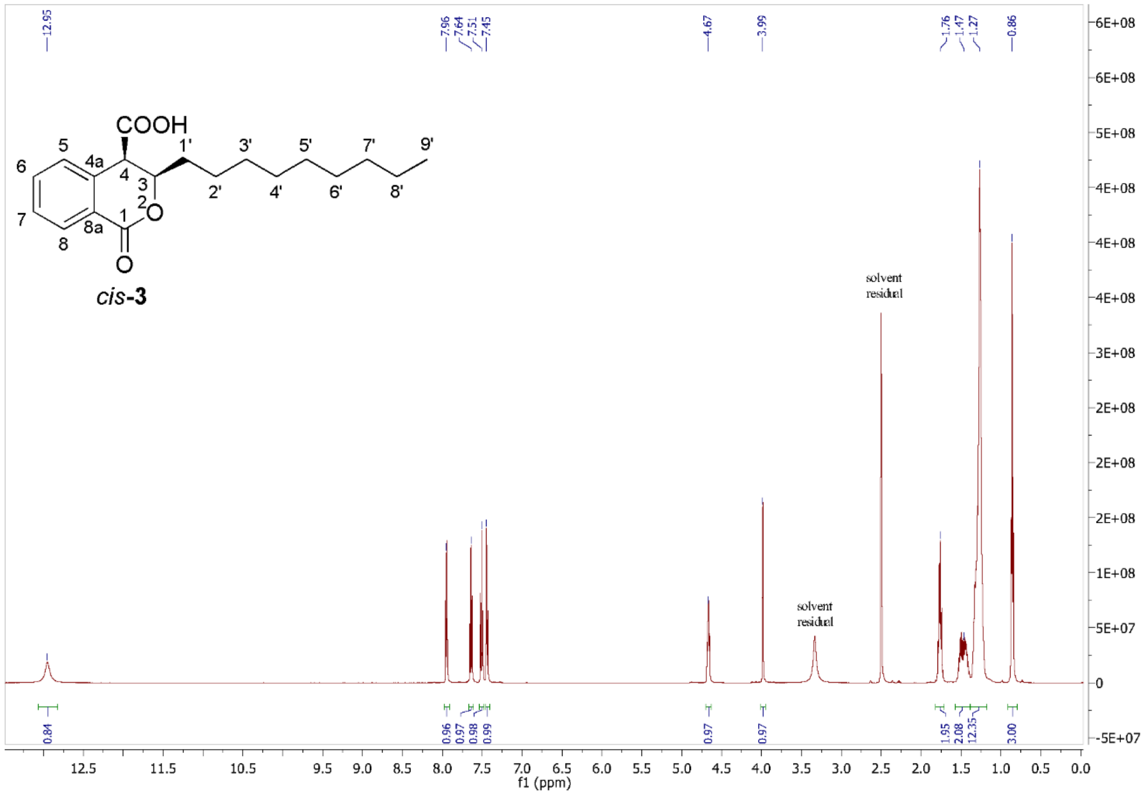

Figure S12. ^{1}**H-NMR Spectrum of** ***cis*****-3 DMSO-d**$_{6}$**.**

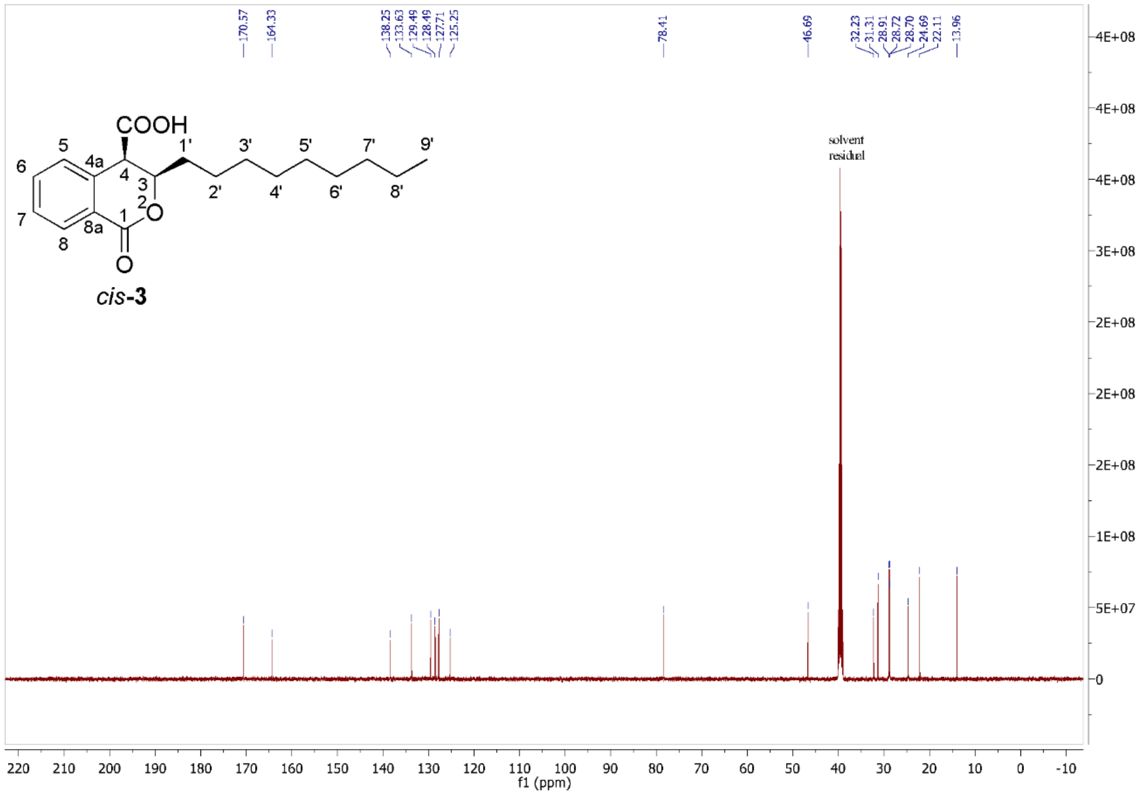

Figure S13. ^{13}**C-NMR Spectrum of** ***cis*****-3 in DMSO-d**$_{6}$**.**

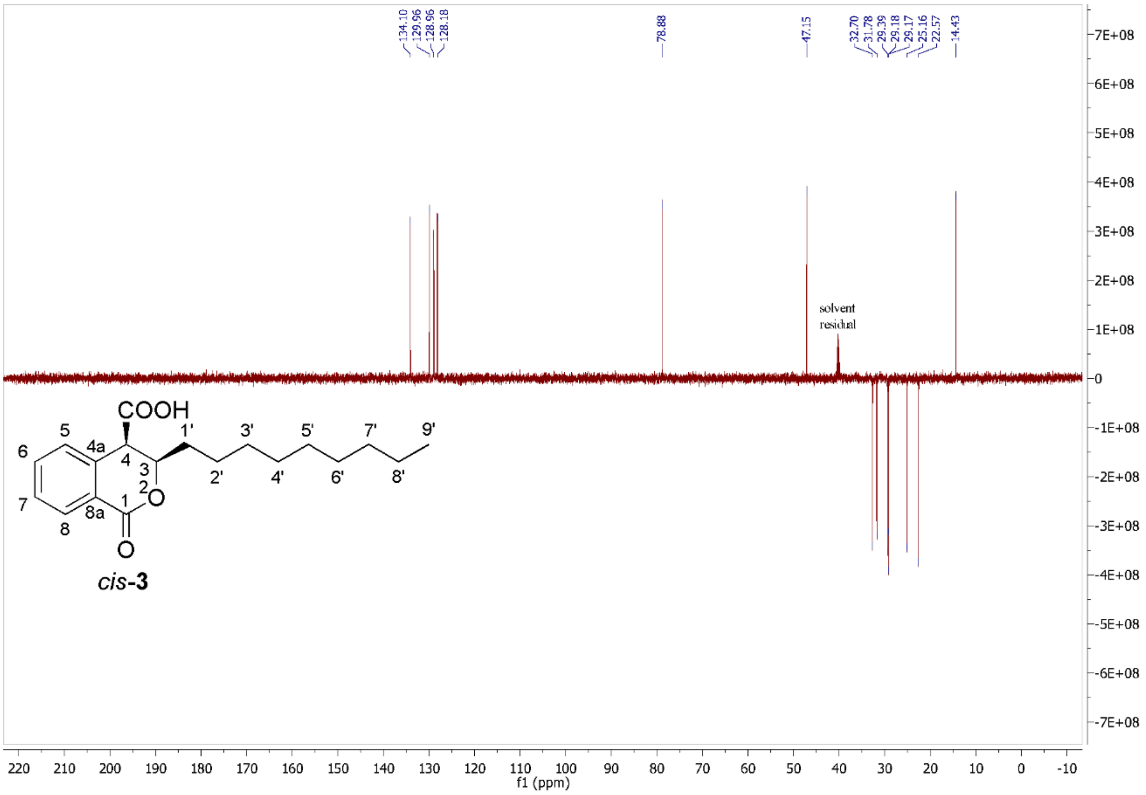

Figure S14. DEPT-135 NMR Spectrum of *cis*-3 in DMSO-d6.

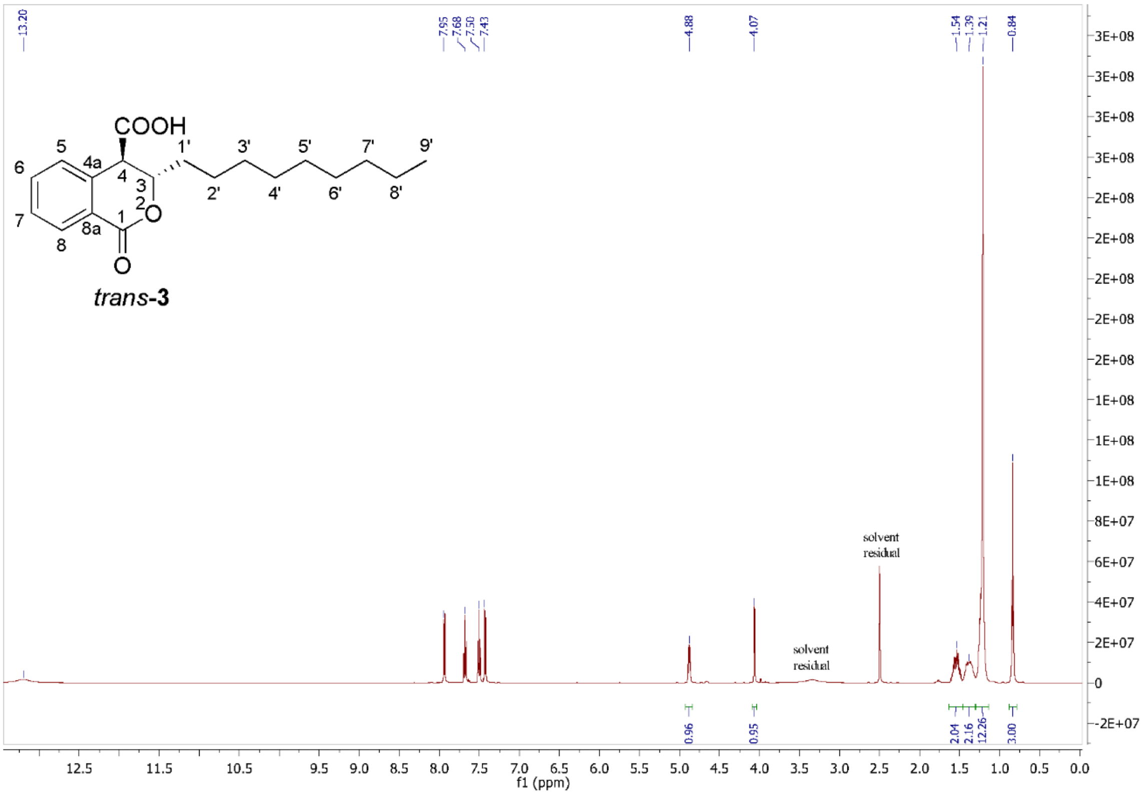

Figure S15. ^{1}H-NMR Spectrum of *trans*-3 DMSO-d6.

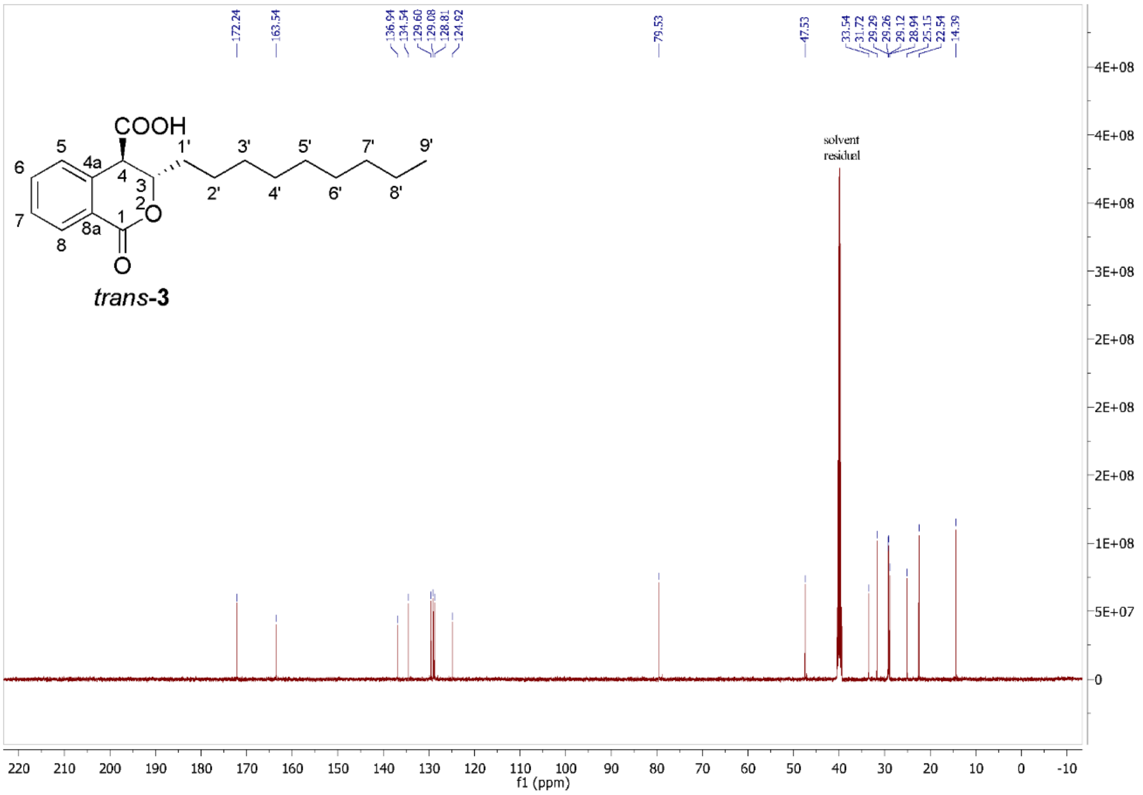

Figure S16. ^{13}**C-NMR Spectrum of** ***trans*****-3 in DMSO-d**$_6$**.**

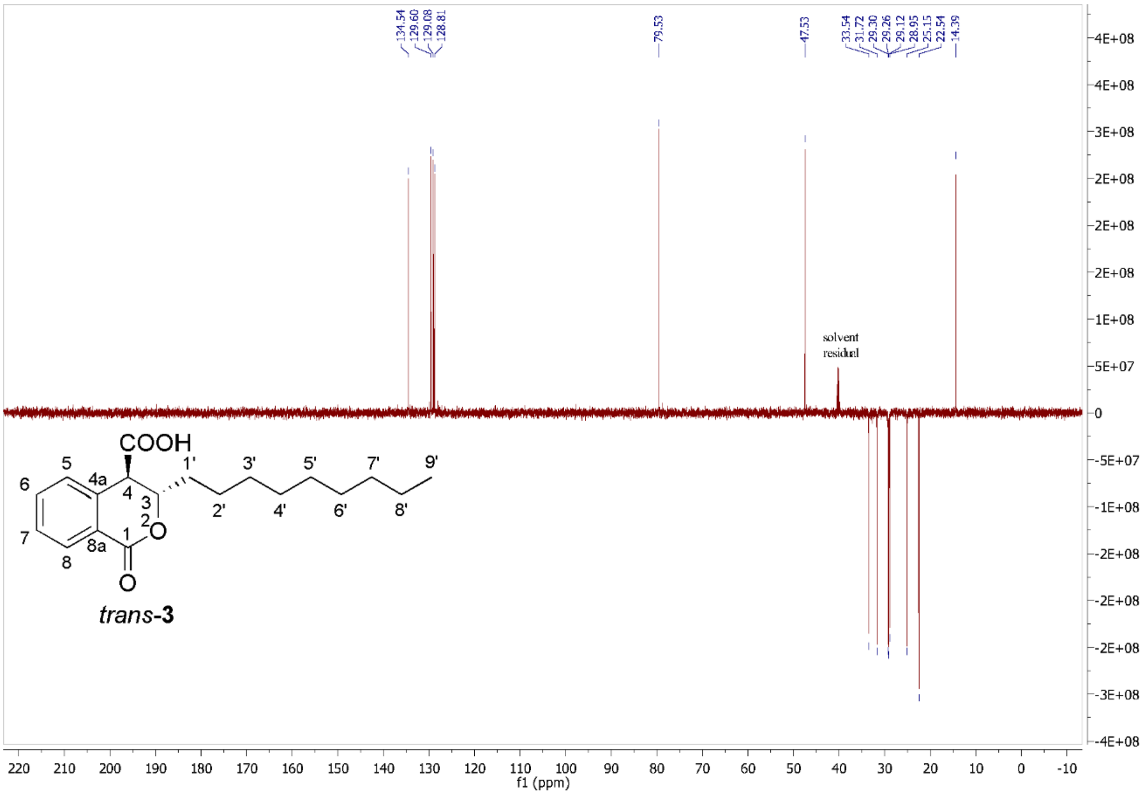

Figure S17. DEPT-135 NMR Spectrum of ***trans*****-3 in DMSO-d**$_6$**.**

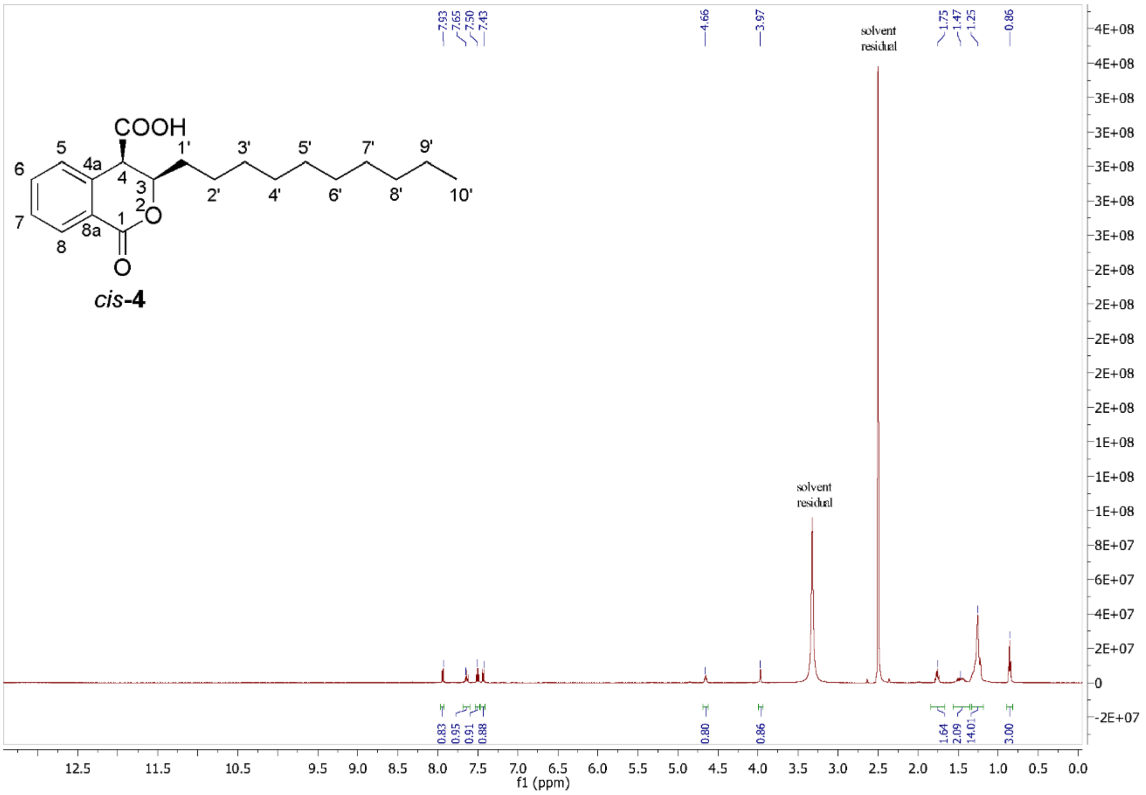

Figure S18. ^{1}H-NMR Spectrum of *cis*-4 DMSO-d6.

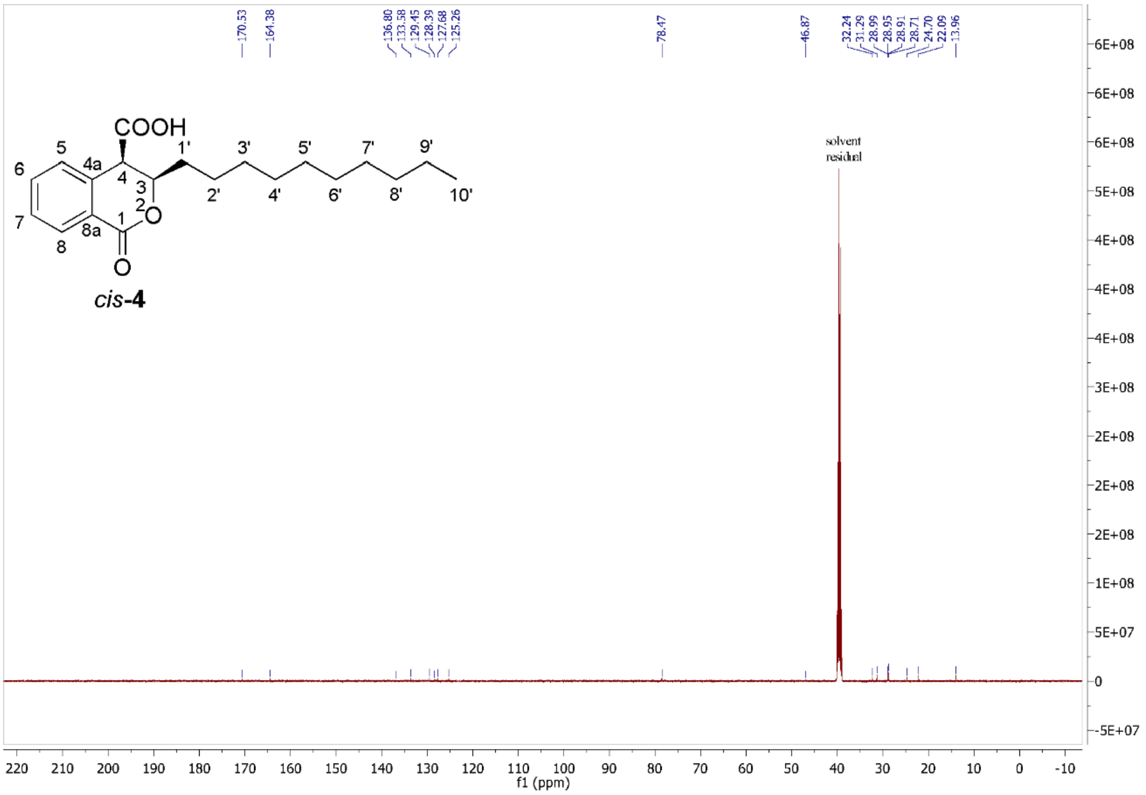

Figure S19. ^{13}C-NMR Spectrum of *cis*-4 in DMSO-d6.

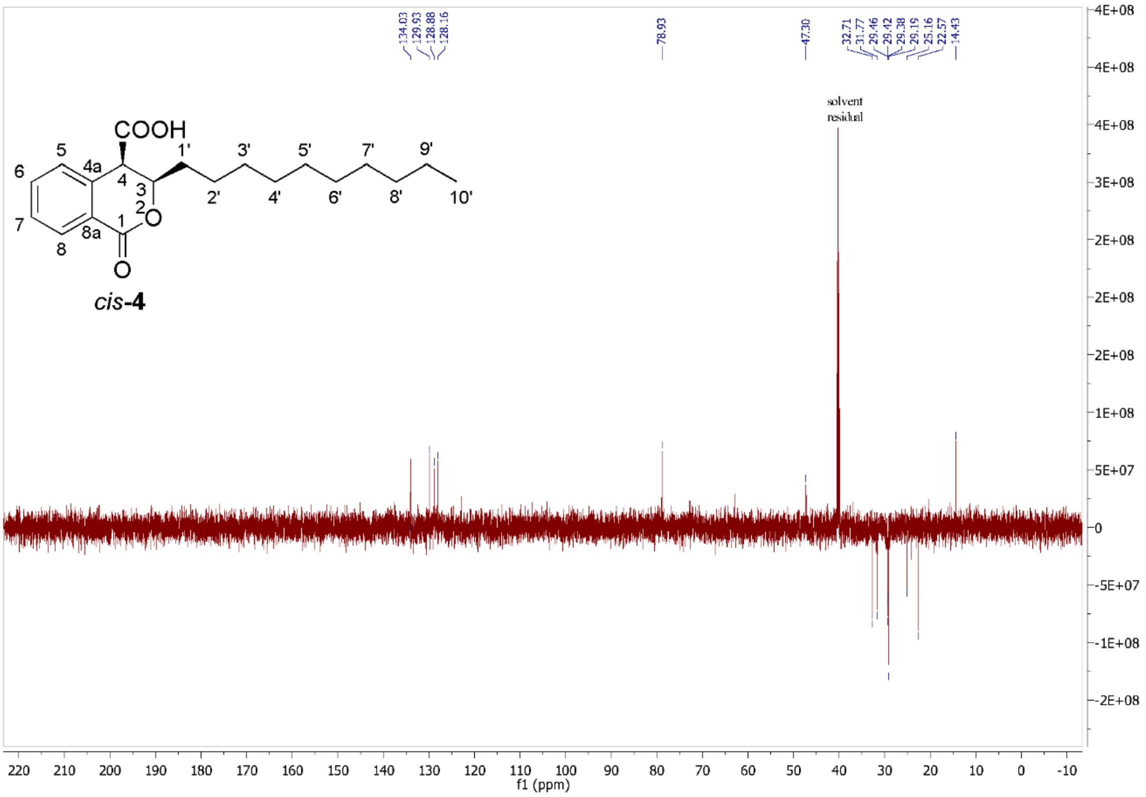

Figure S20. DEPT-135 NMR Spectrum of *cis*-4 in DMSO-d6.

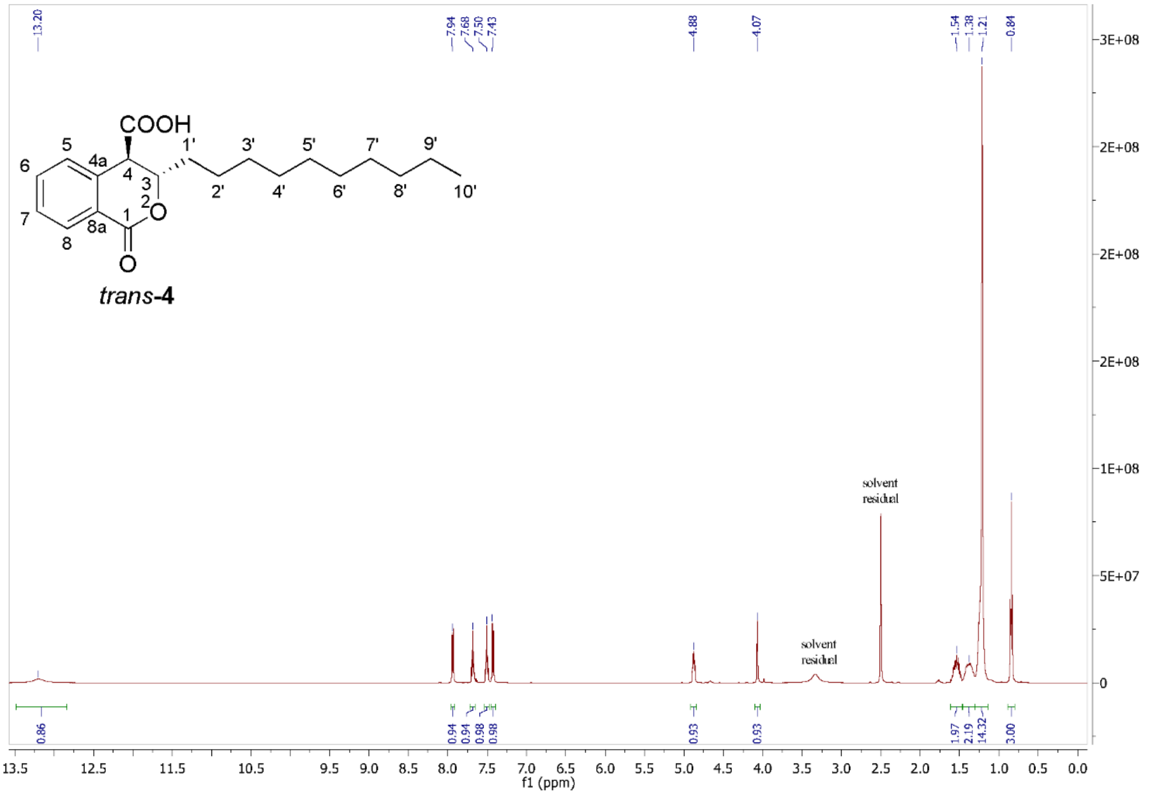

Figure S21. ^{1}H-NMR Spectrum of *trans*-4 DMSO-d6.

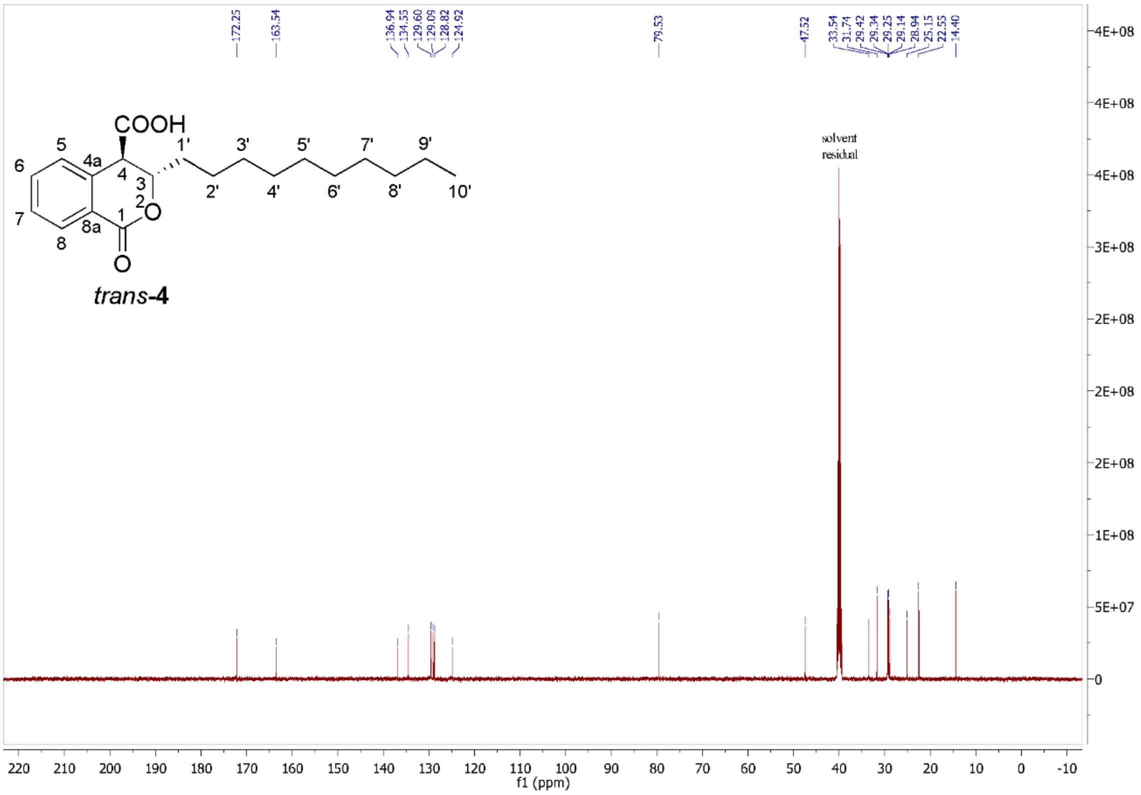

Figure S22. ^{13}**C-NMR Spectrum of** ***trans*-4 in DMSO-d$_6$.**

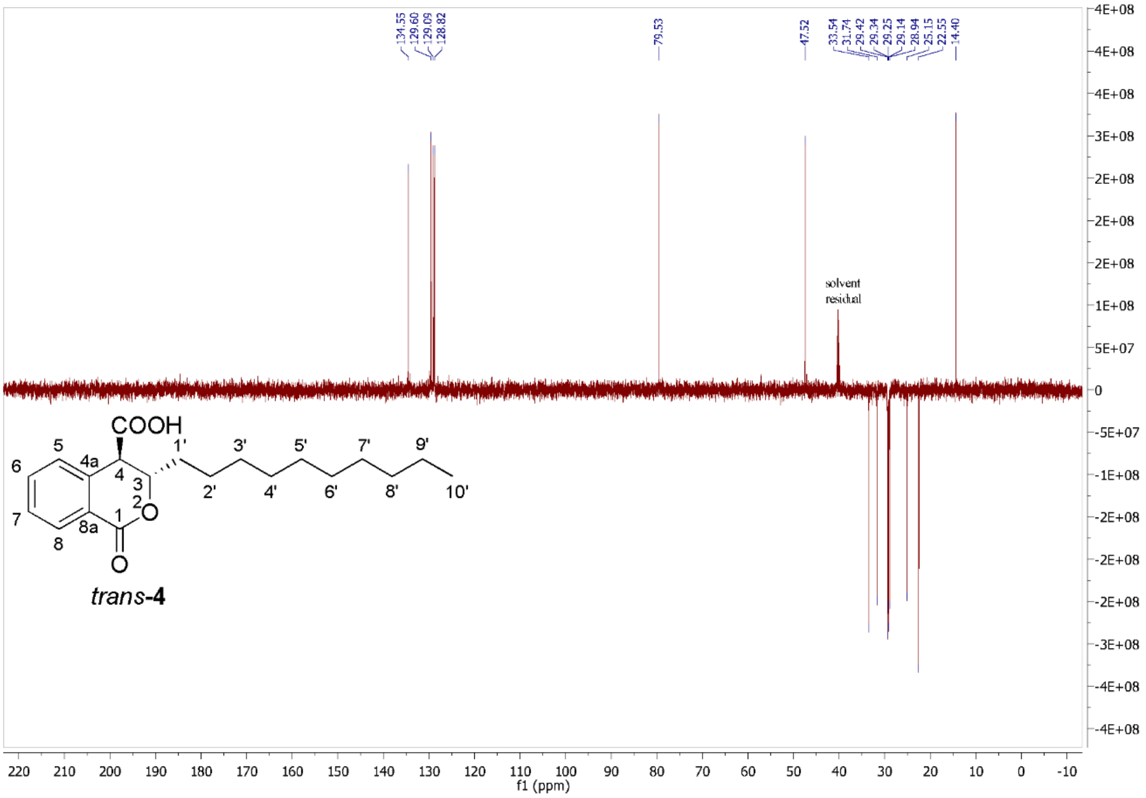

Figure S23. DEPT-135 NMR Spectrum of ***trans*-4 in DMSO-d$_6$.**

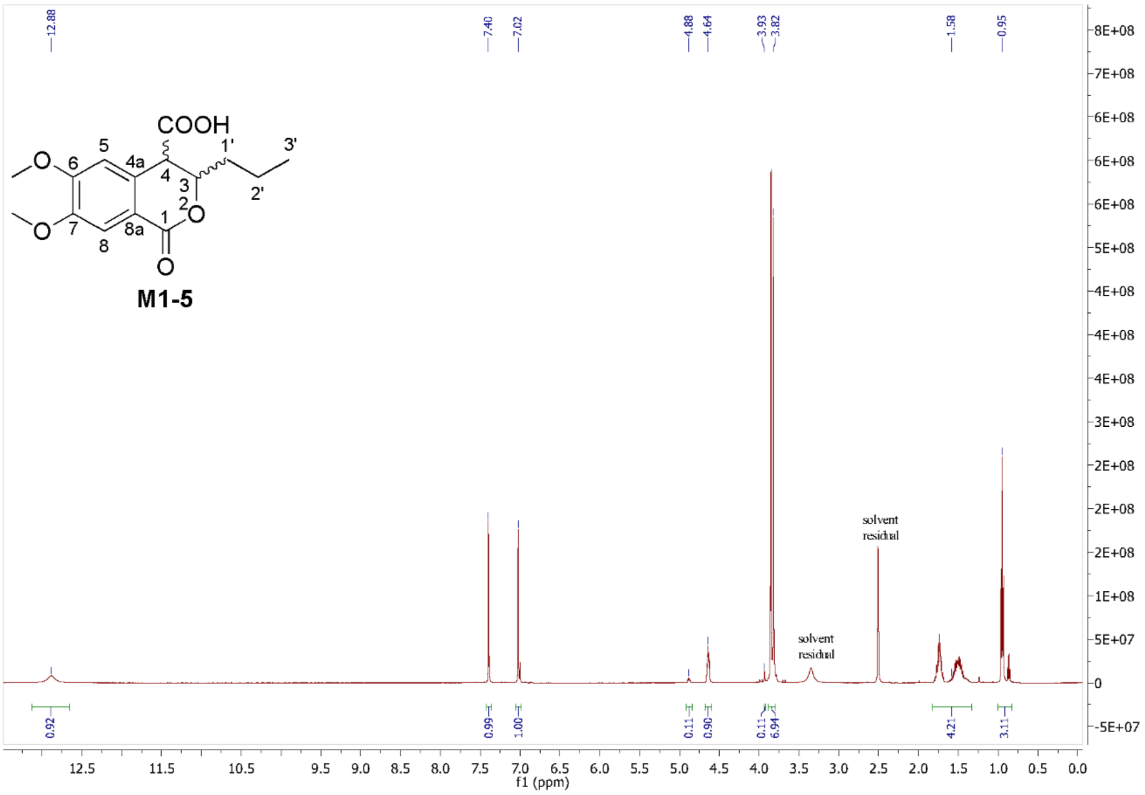

Figure S24. **¹H-NMR Spectrum of M1-5 DMSO-d₆.**

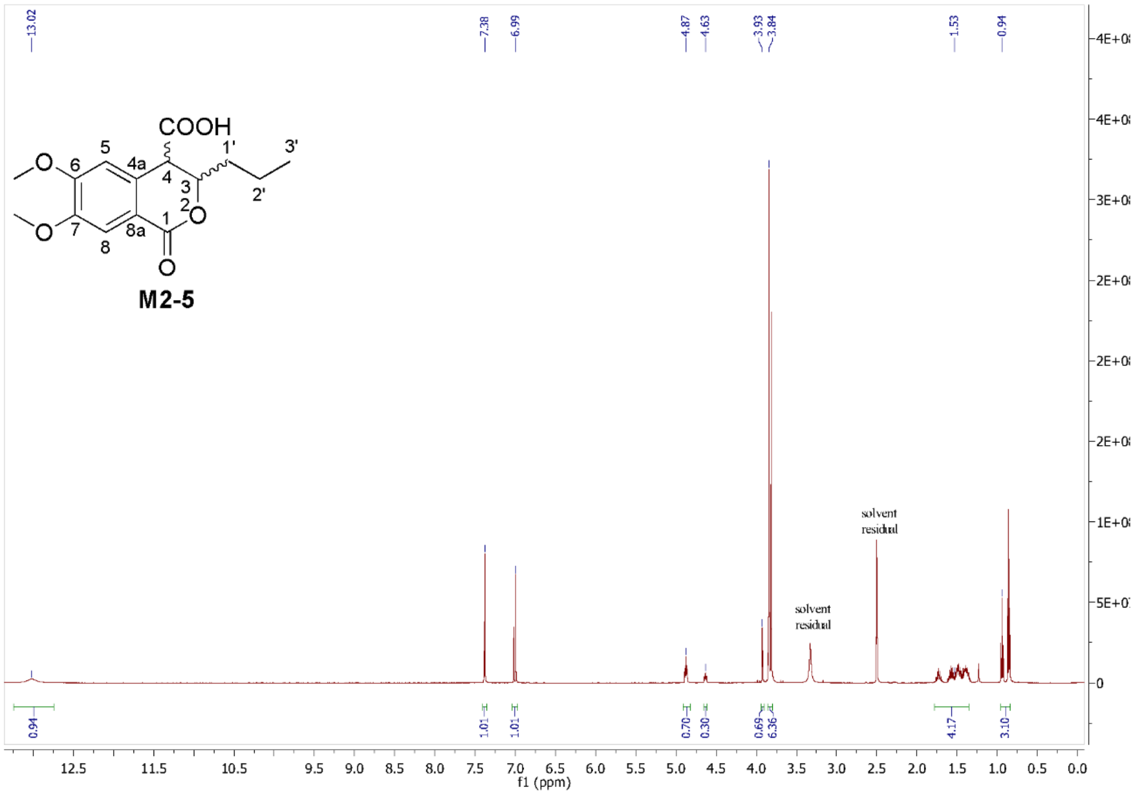

Figure S25. **¹H-NMR Spectrum of M2-5 DMSO-d₆.**

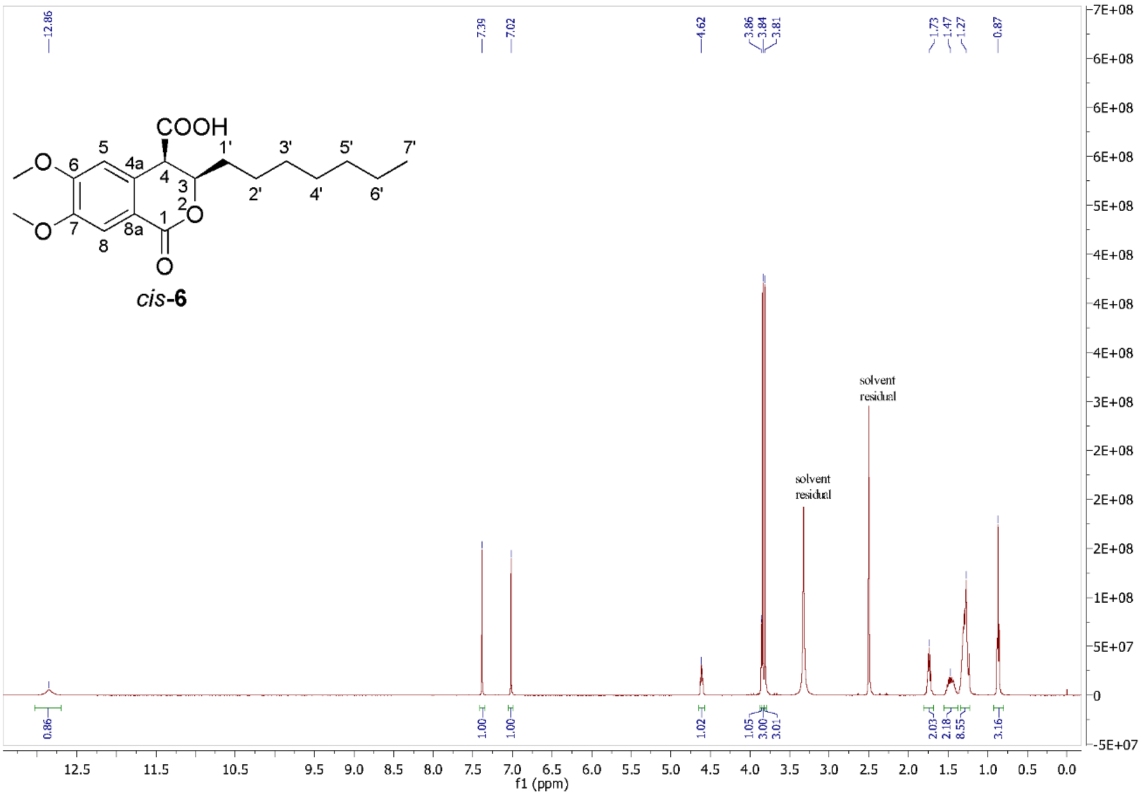

Figure S26. ^{1}H-NMR Spectrum of *cis*-6 DMSO-d6.

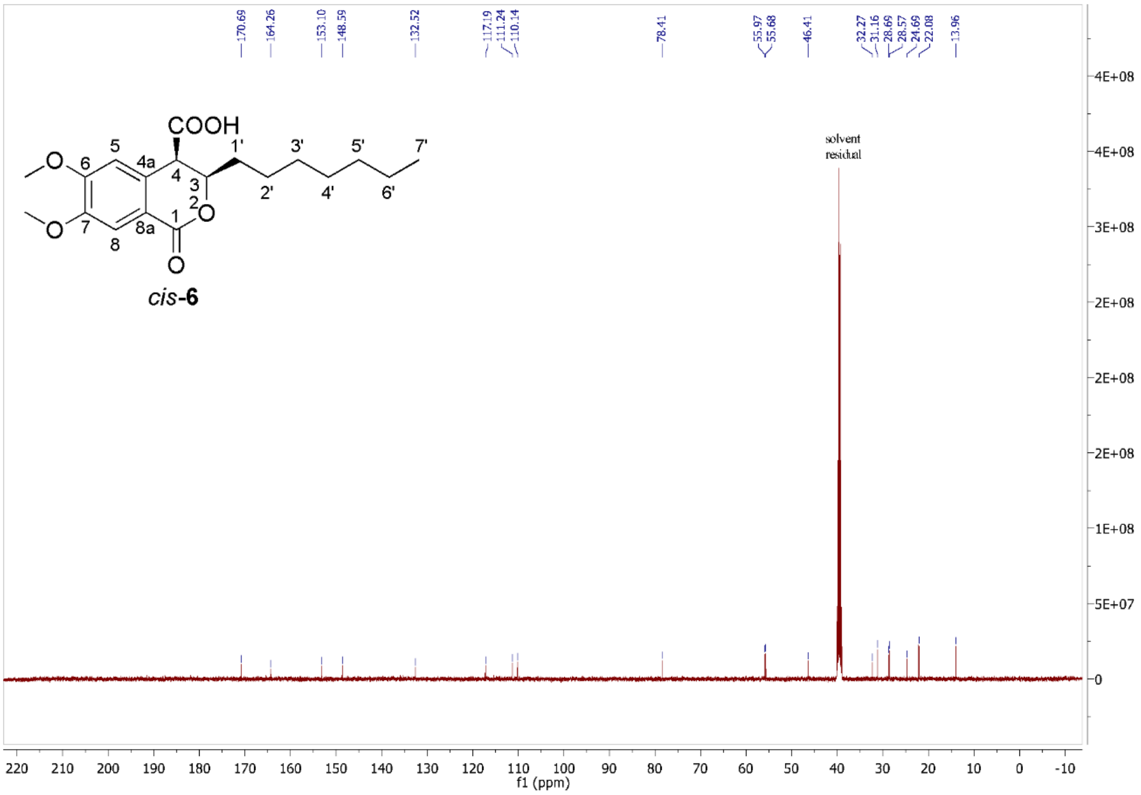

Figure S27. ^{13}C-NMR Spectrum of *cis*-6 in DMSO-d6.

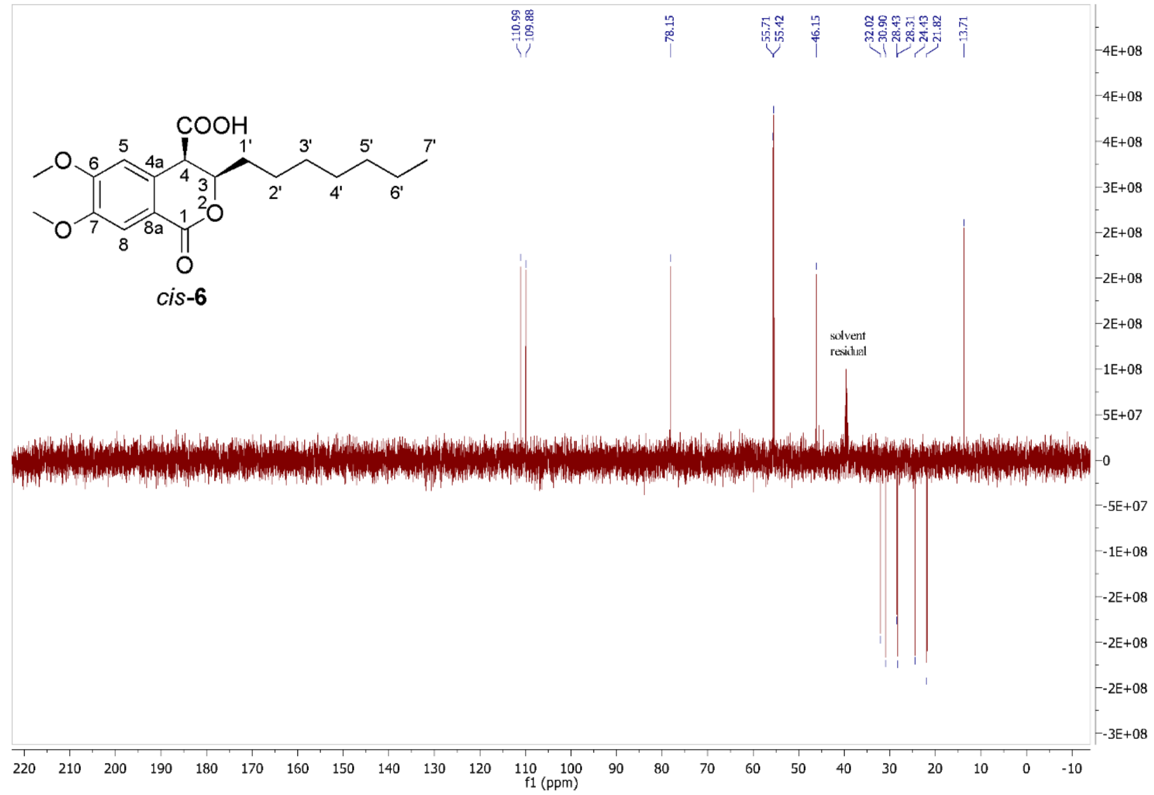

Figure S28. DEPT-135 NMR Spectrum of *cis*-6 in DMSO-d6.

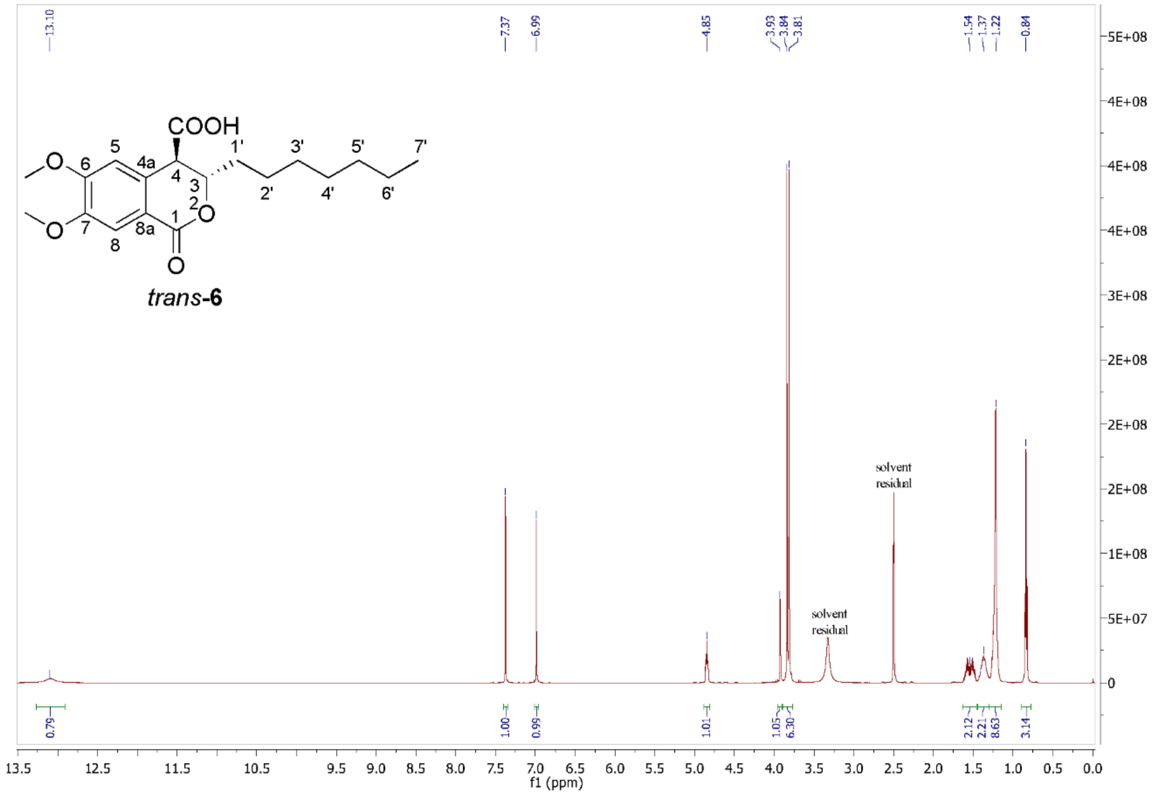

Figure S29. ^{1}H-NMR Spectrum of *trans*-6 DMSO-d6.

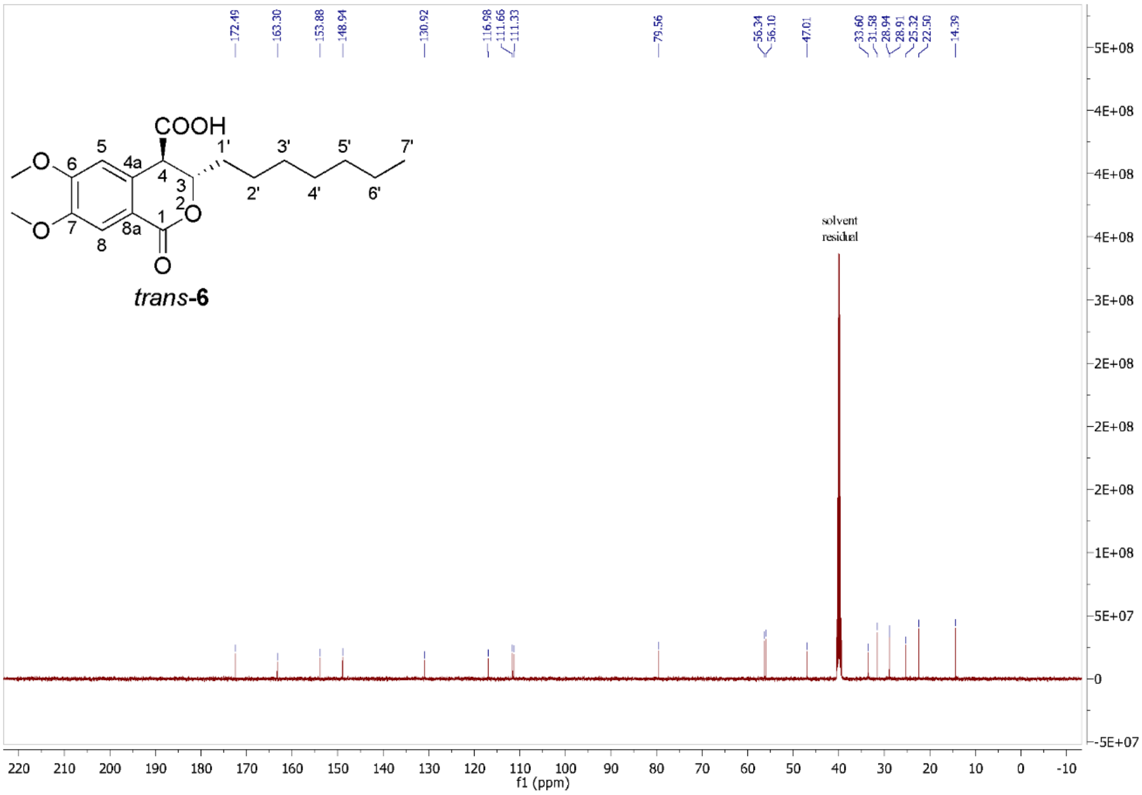

Figure S30. ^{13}**C-NMR Spectrum of** ***trans*****-6 in DMSO-d$_6$.**

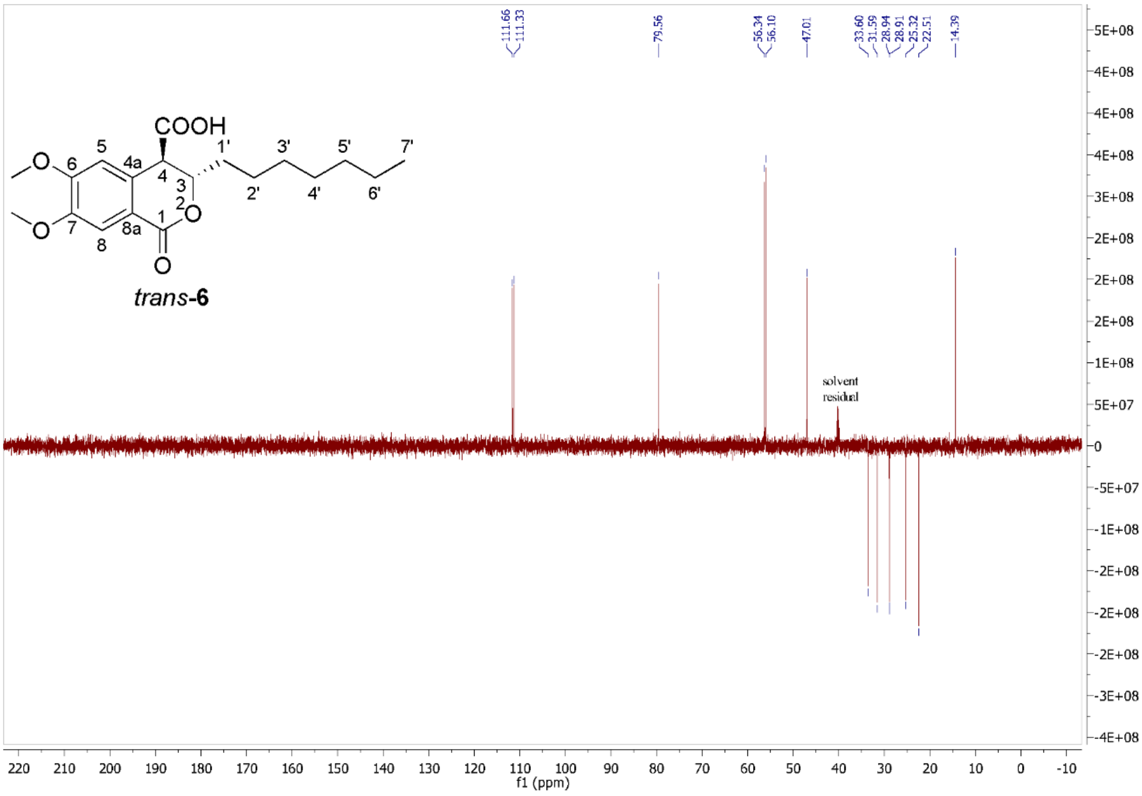

Figure S31. DEPT-135 NMR Spectrum of ***trans*****-6 in DMSO-d$_6$.**

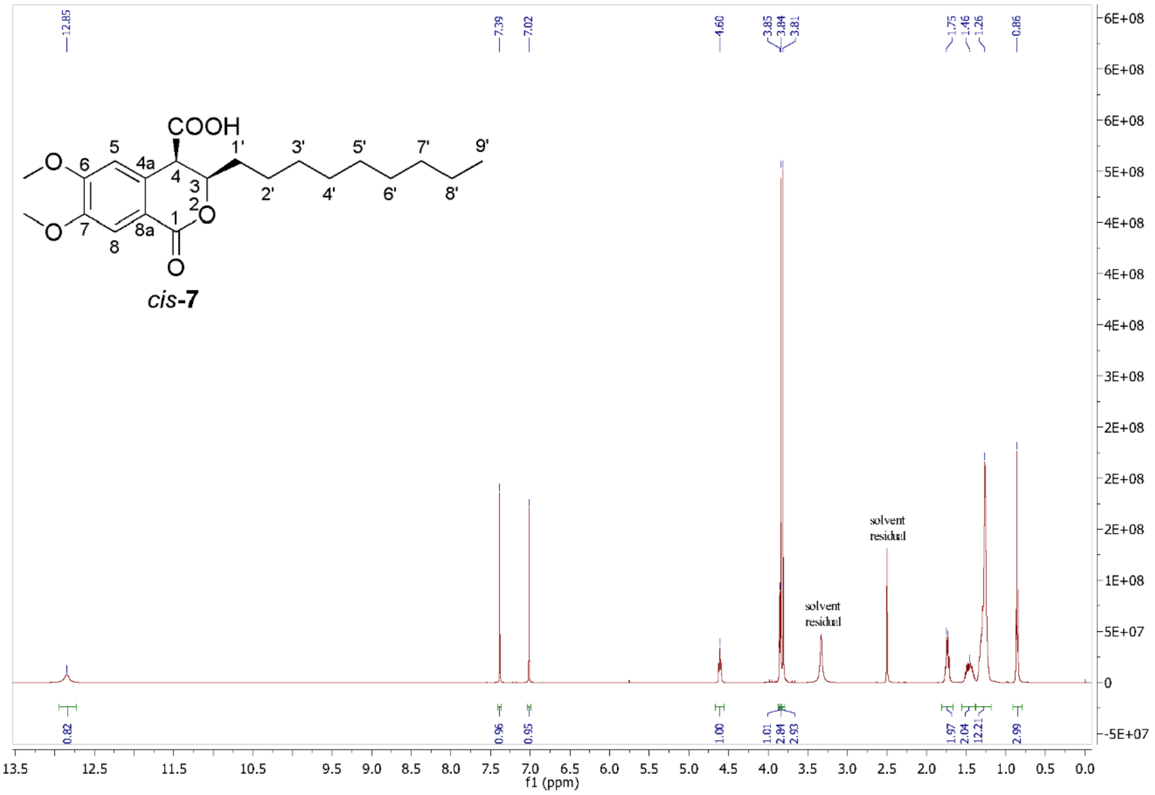

Figure S32. **^{1}H-NMR Spectrum of *cis*-7 DMSO-d6.**

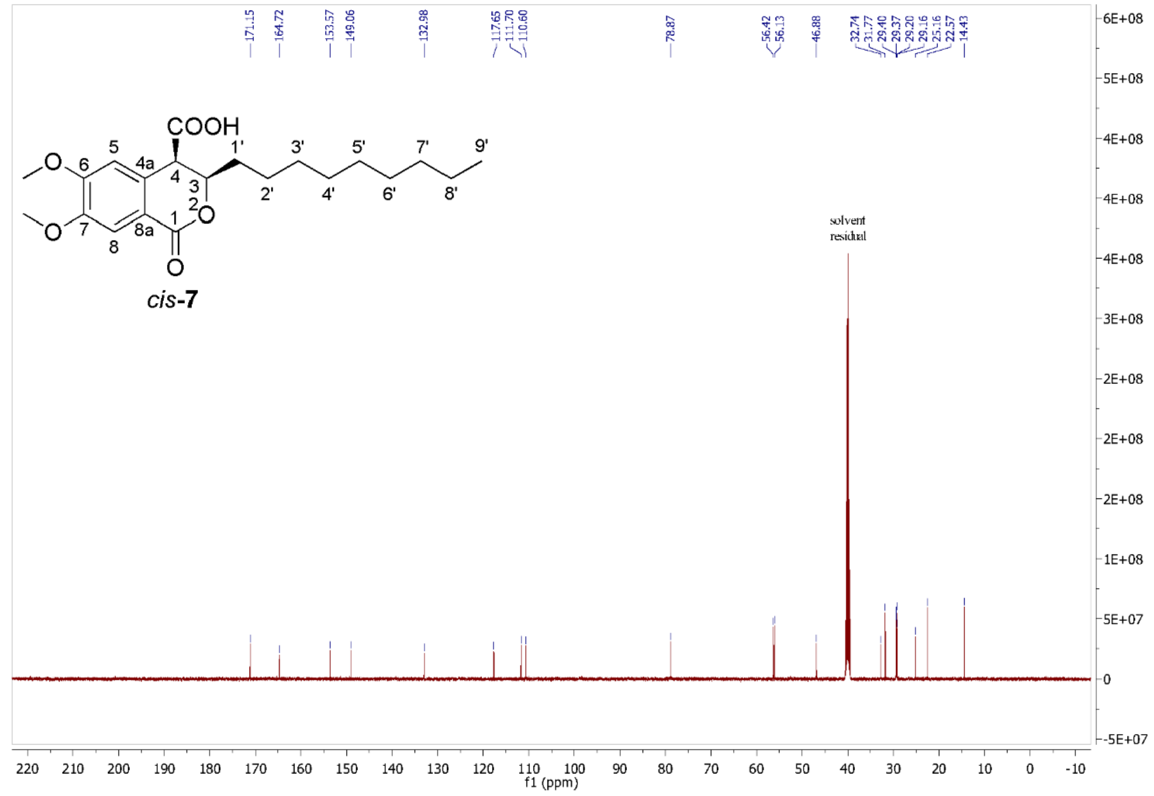

Figure S33. **^{13}C-NMR Spectrum of *cis*-7 in DMSO-d6.**

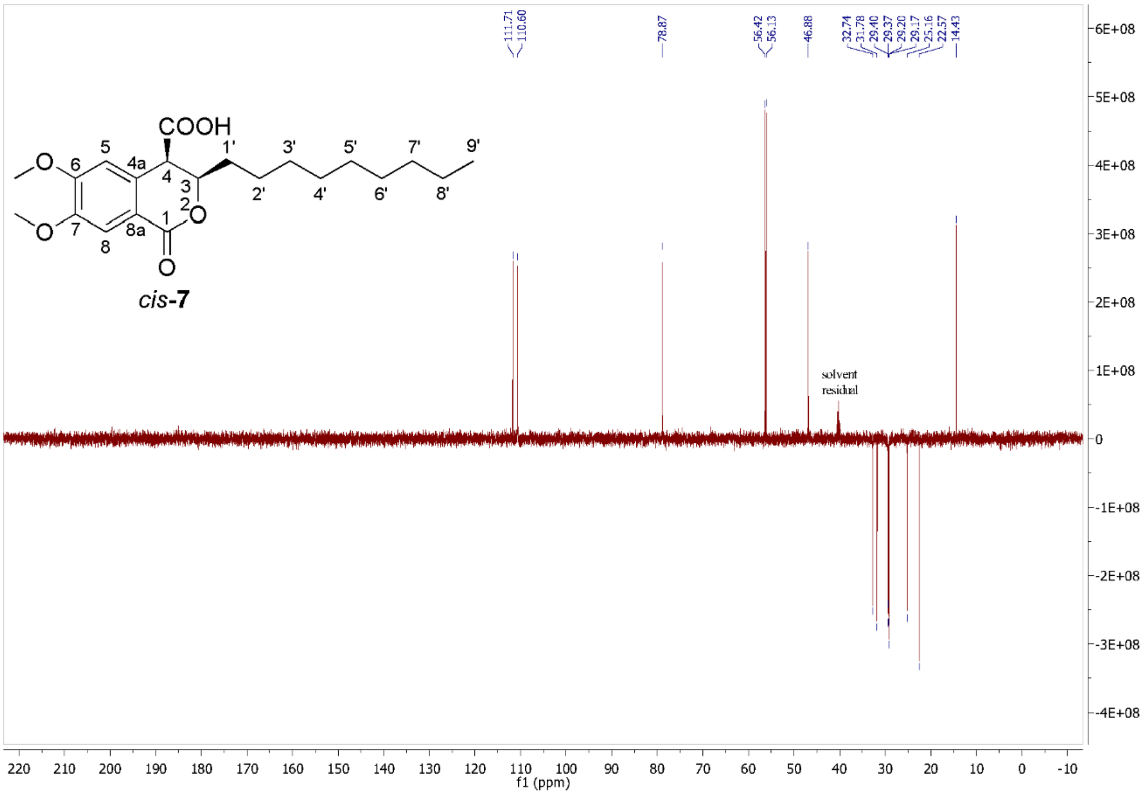

Figure S34. DEPT-135 NMR Spectrum of *cis*-7 in DMSO-d6.

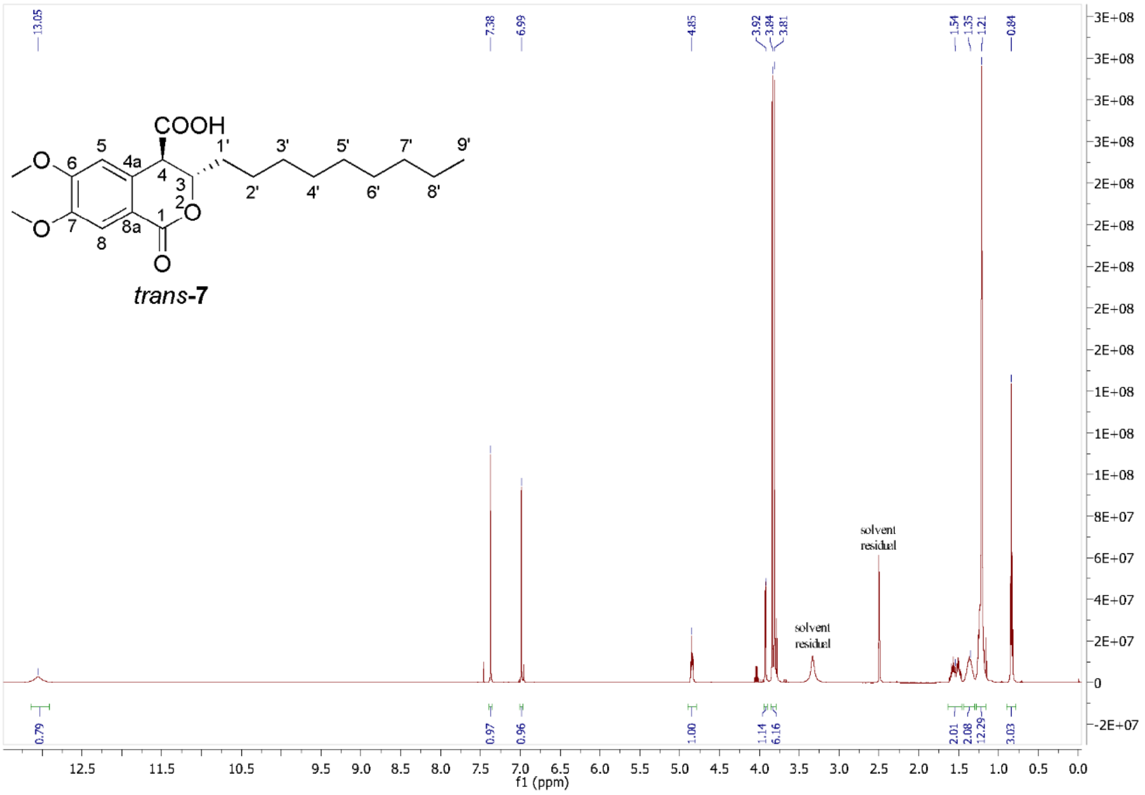

Figure S35. ^{1}H-NMR Spectrum of *trans*-7 DMSO-d6.

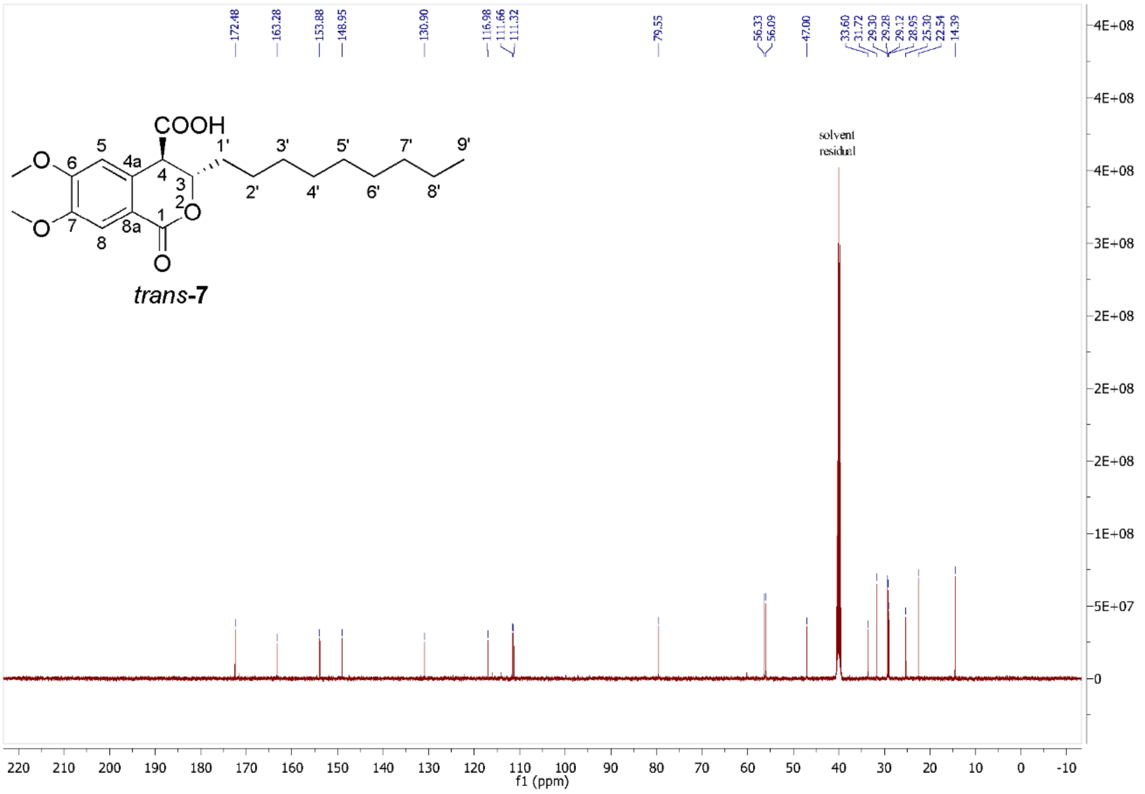

Figure S36. ^{13}C-NMR Spectrum of *trans*-7 in DMSO-d6.

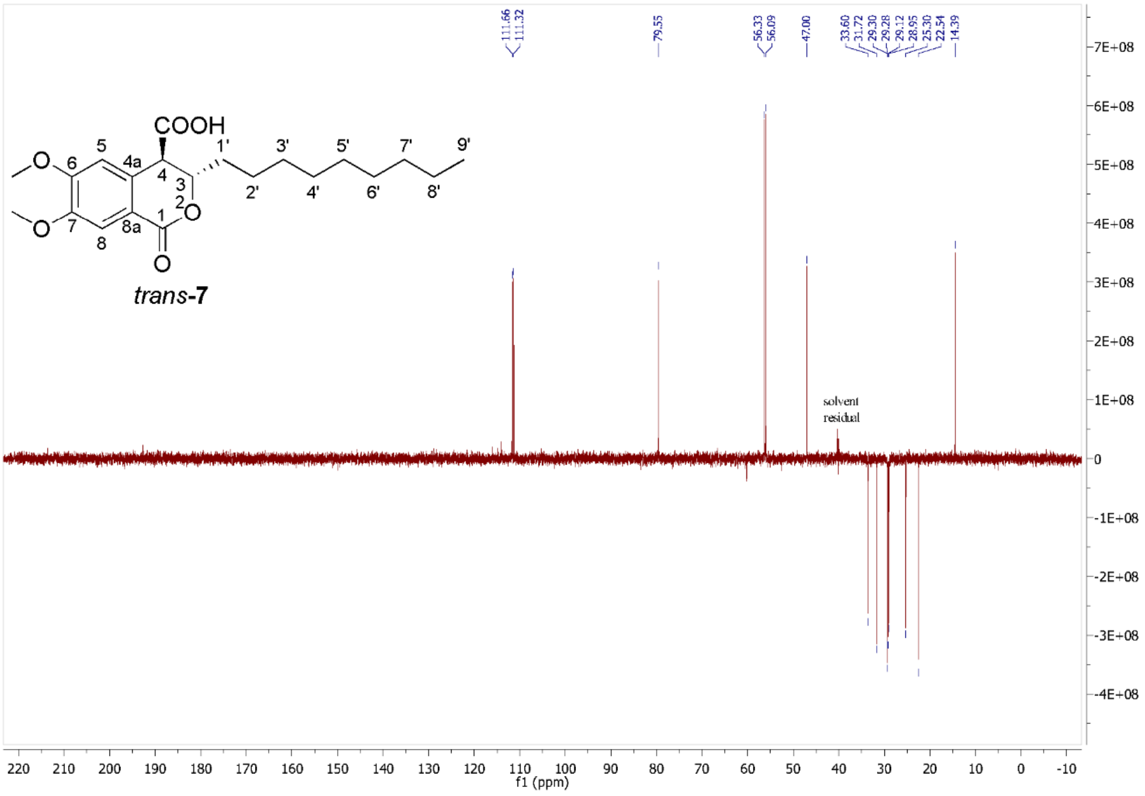

Figure S37. DEPT-135 NMR Spectrum of *trans*-7 in DMSO-d6.

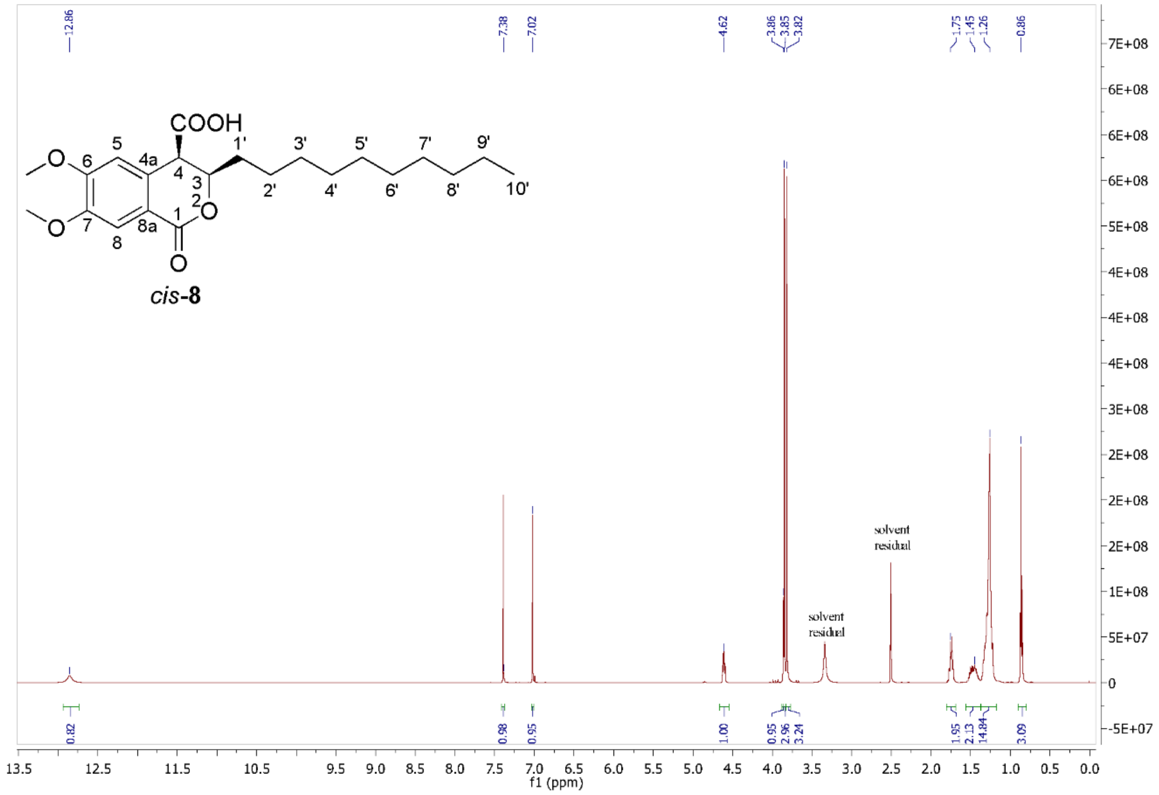

Figure S38. ^{1}**H-NMR Spectrum of** ***cis*****-8 DMSO-d$_6$.**

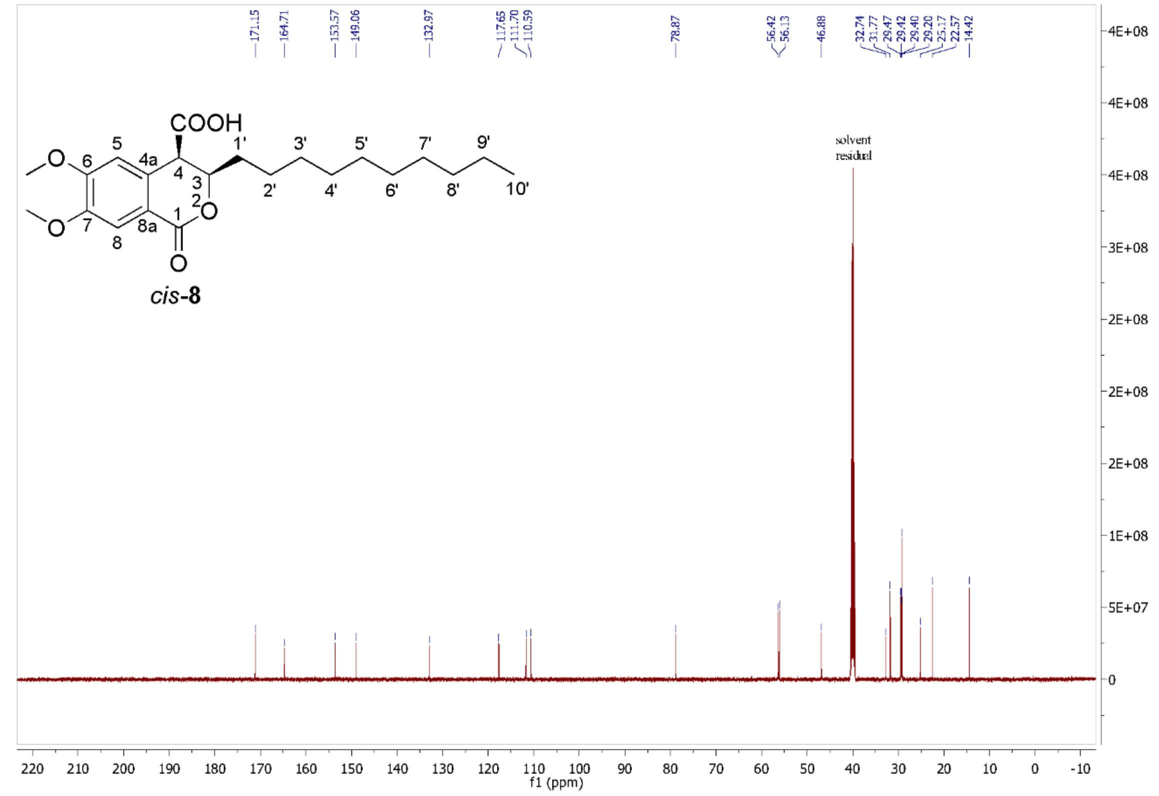

Figure S39. ^{13}**C-NMR Spectrum of** ***cis*****-8 in DMSO-d$_6$.**

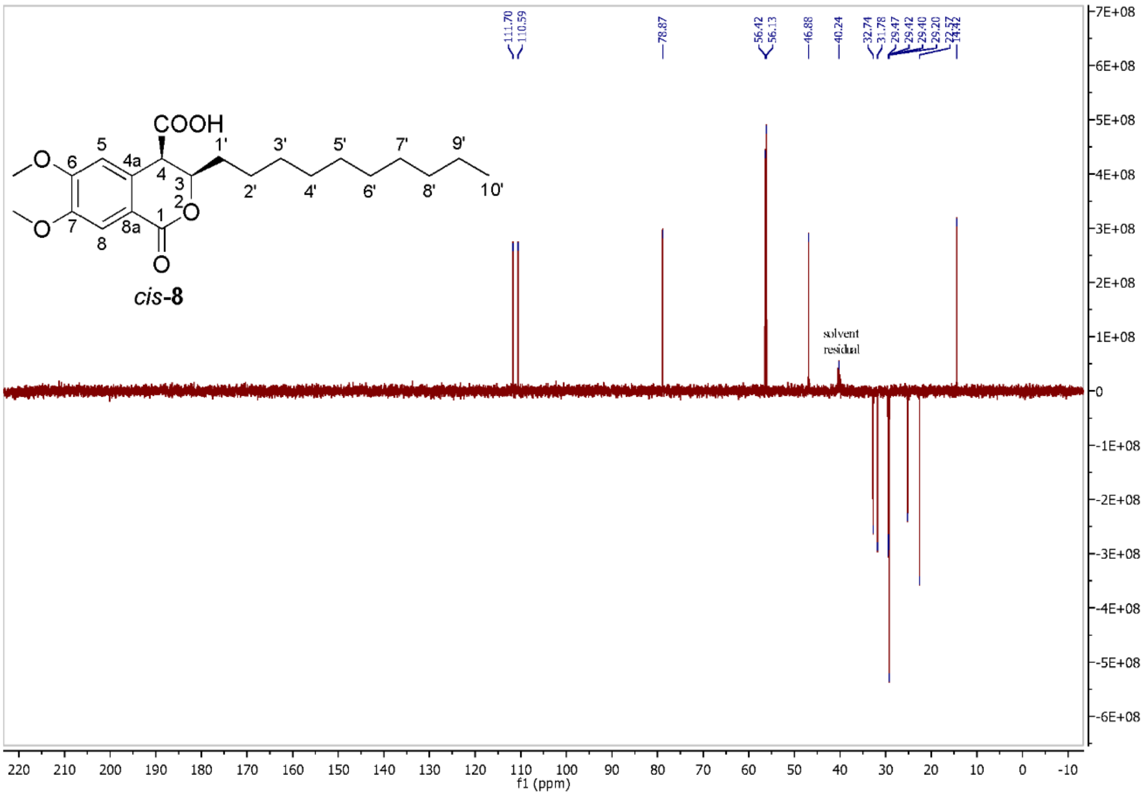

Figure S40. DEPT-135 NMR Spectrum of *cis*-8 in DMSO-d6.

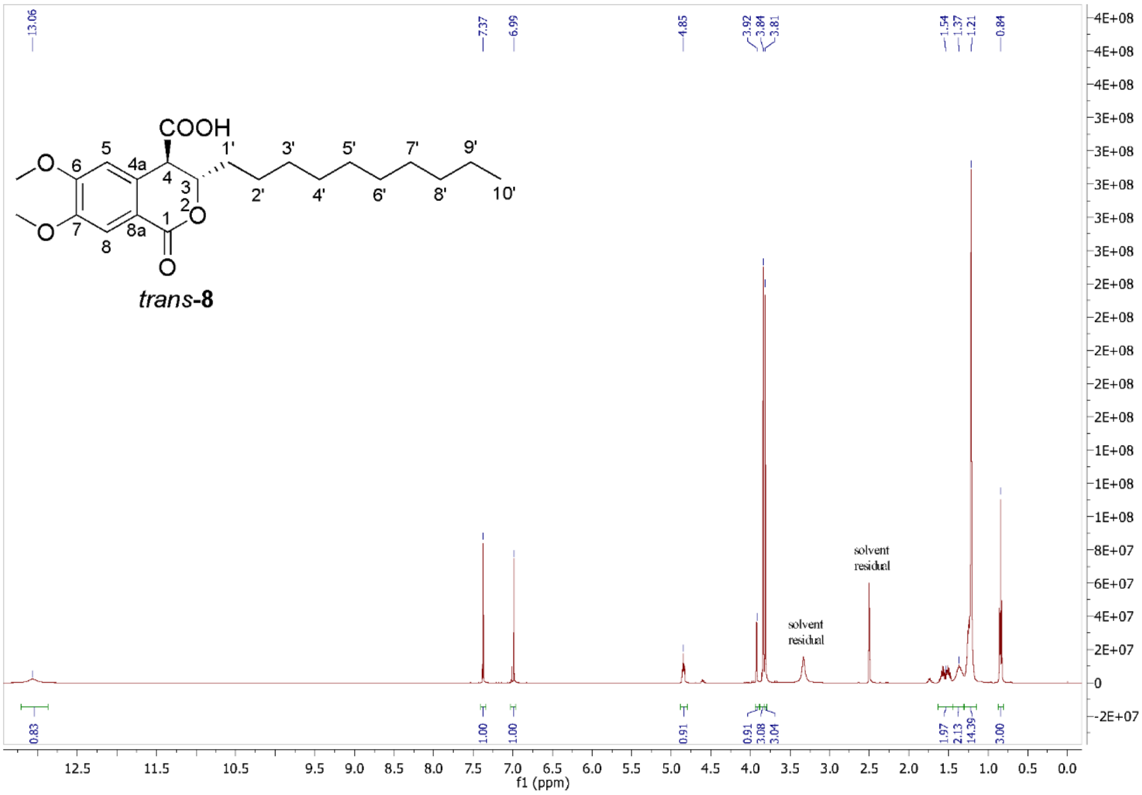

Figure S41. ^{1}H-NMR Spectrum of *trans*-8 DMSO-d6.

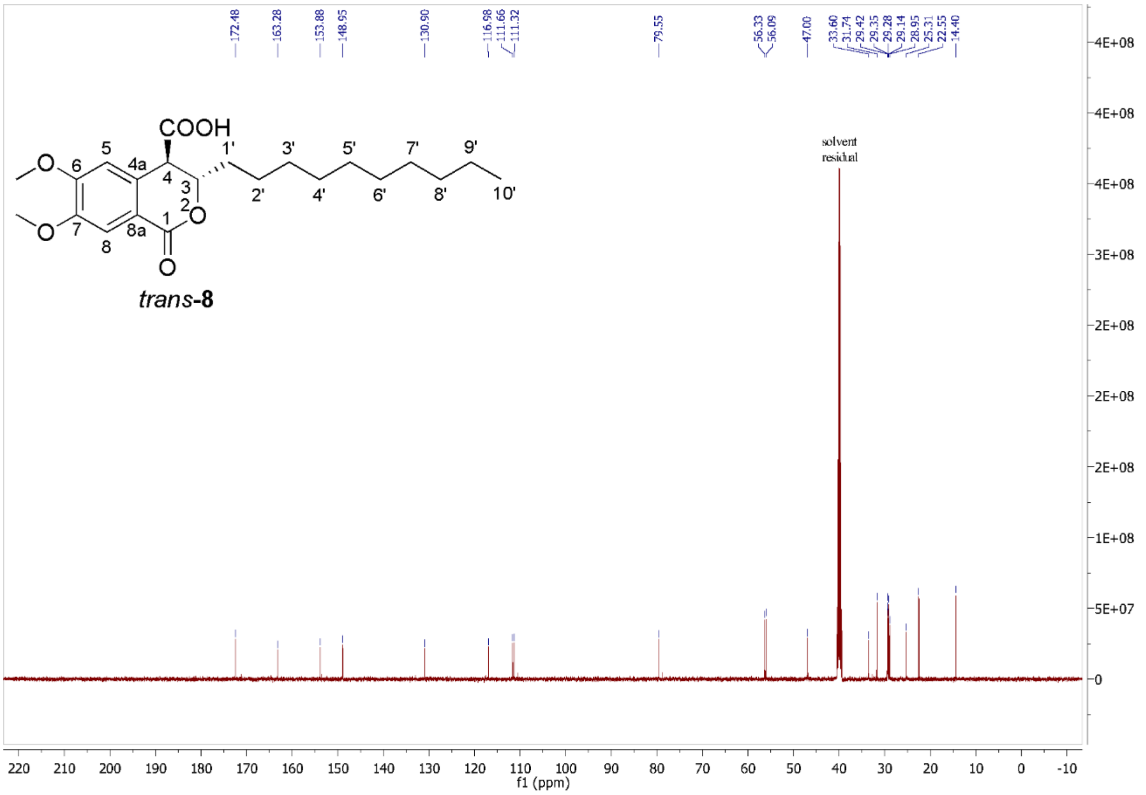

Figure S42. ^{13}**C-NMR Spectrum of** ***trans*****-8 in DMSO-d$_6$.**

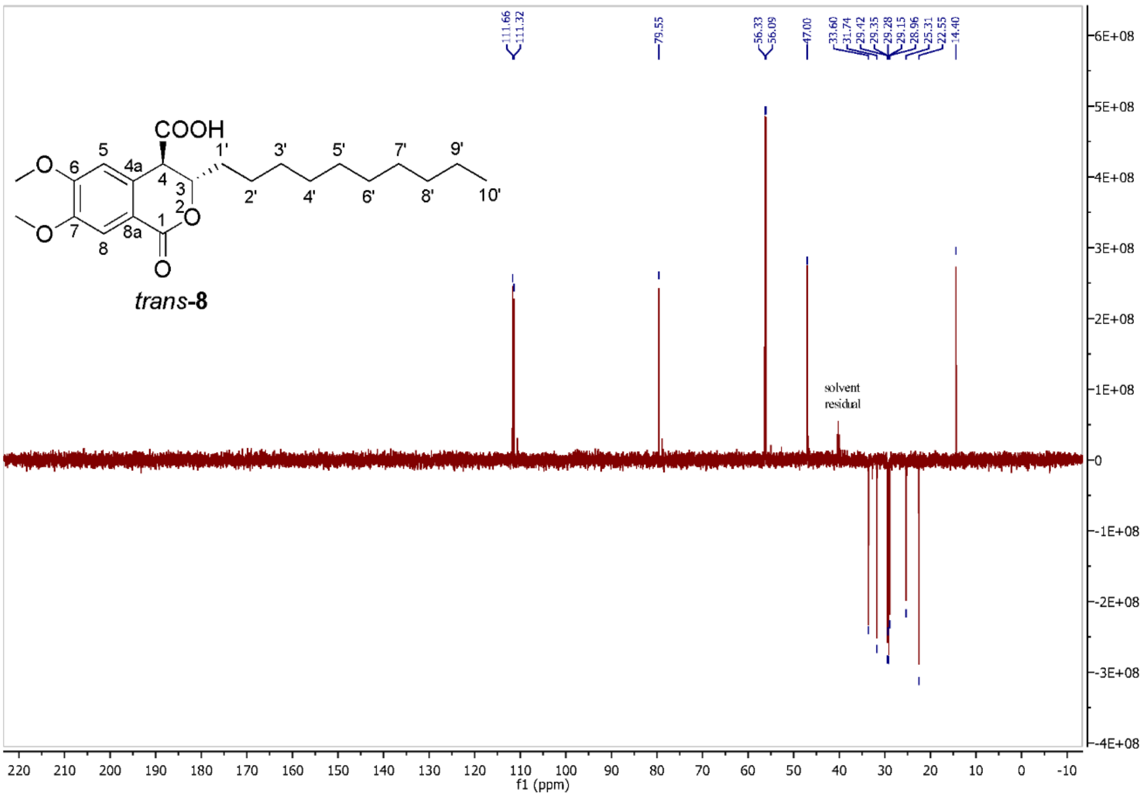

Figure S43. DEPT-135 NMR Spectrum of ***trans*****-8 in DMSO-d$_6$.**

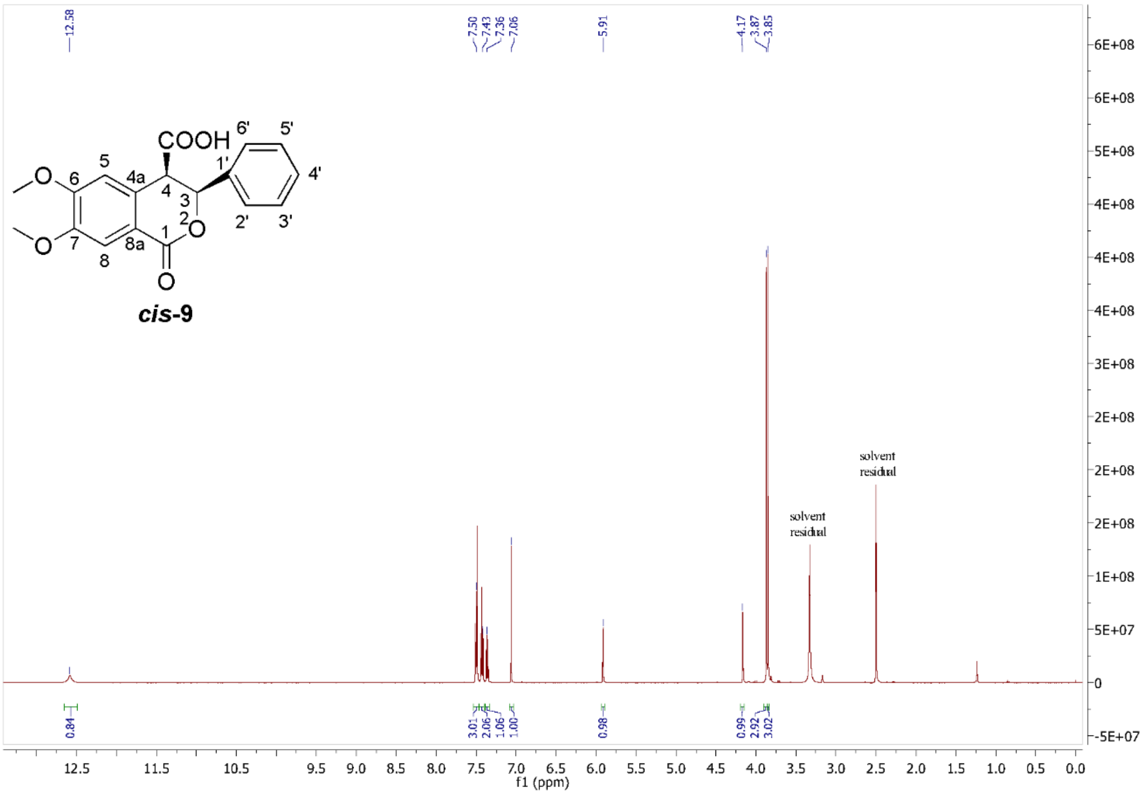

Figure S44. **^{1}H-NMR Spectrum of *cis*-9 DMSO-d6.**

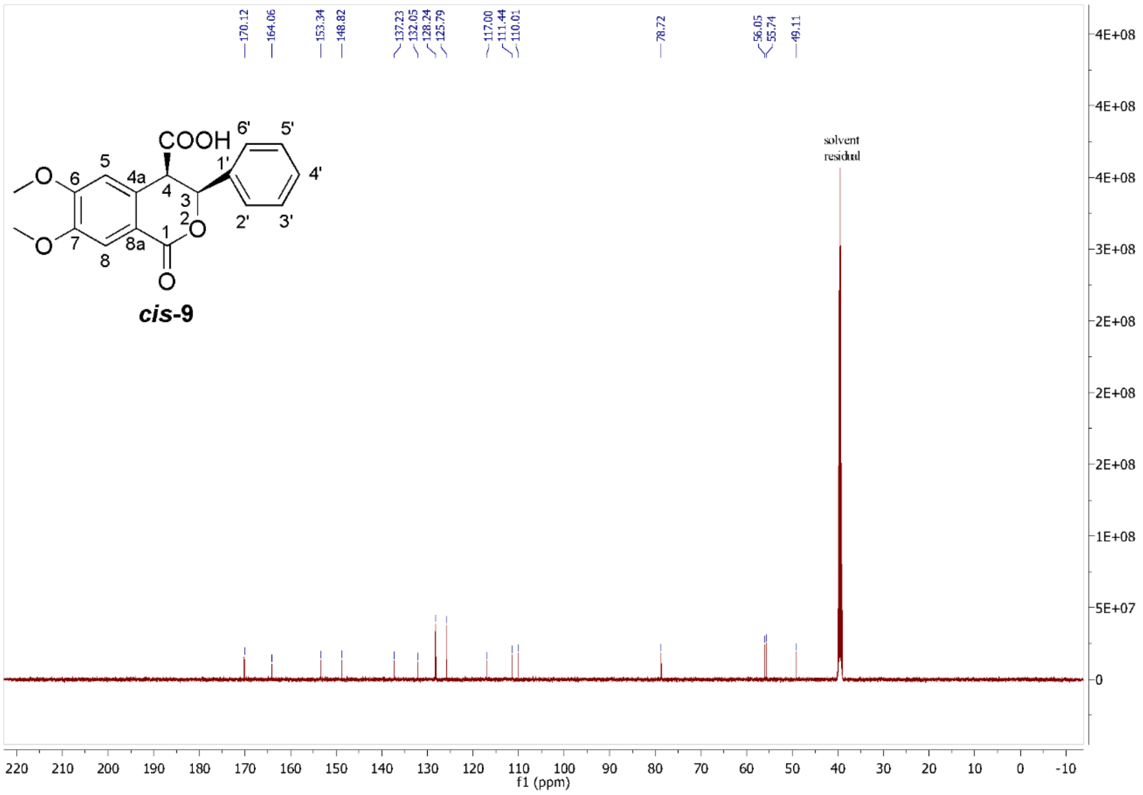

Figure S45. **^{13}C-NMR Spectrum of *cis*-9 in DMSO-d6.**

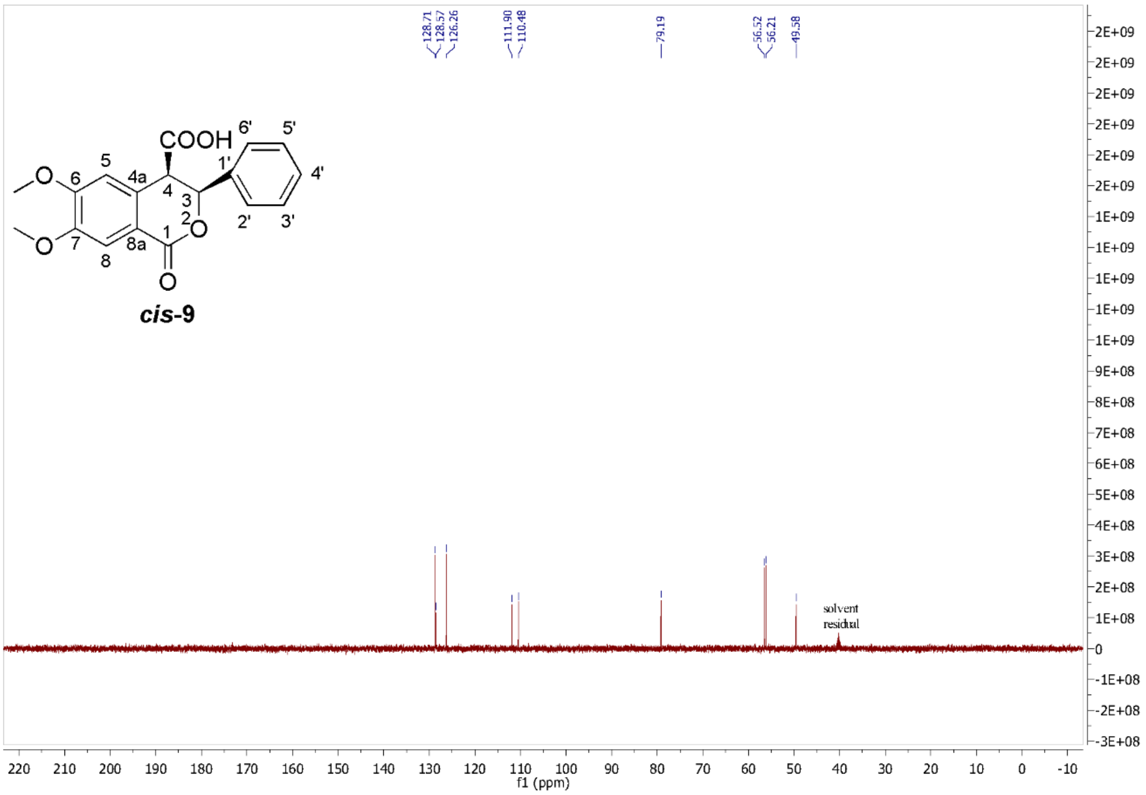

Figure S46. DEPT-135 NMR Spectrum of *cis*-9 in DMSO-d6.

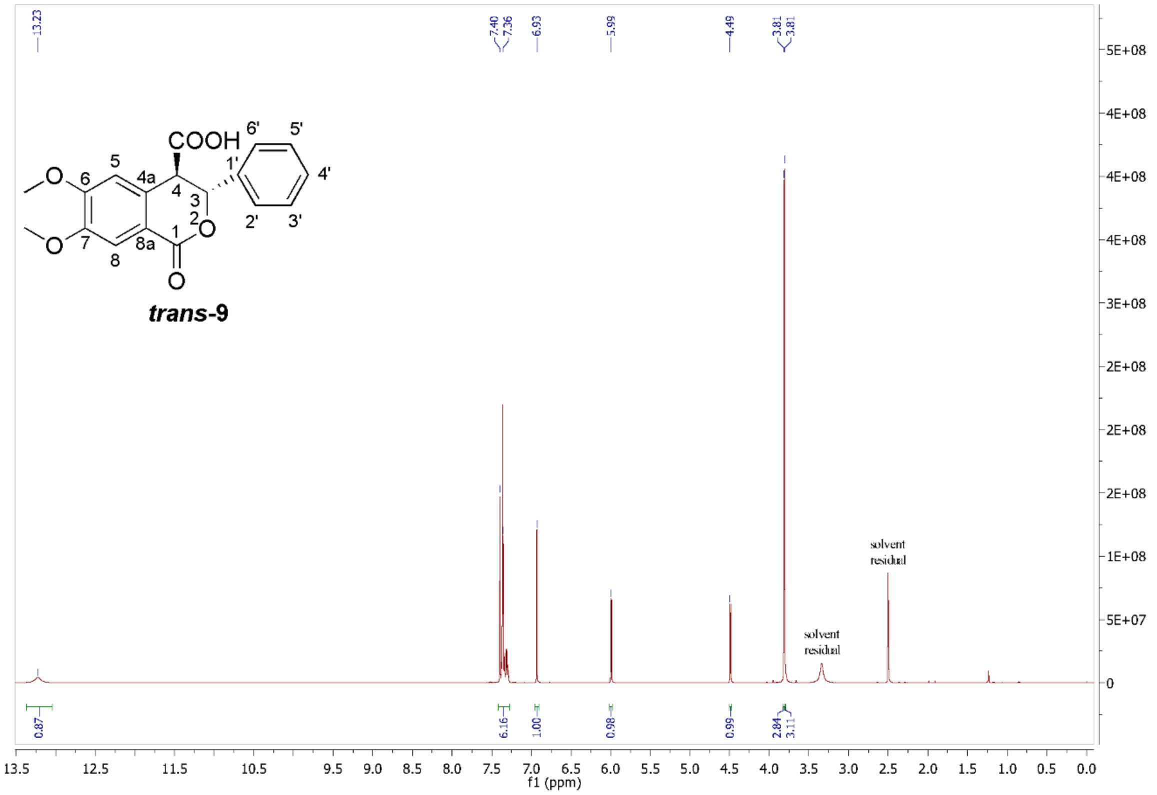

Figure S47. ^{1}H-NMR Spectrum of *trans*-9 DMSO-d6.

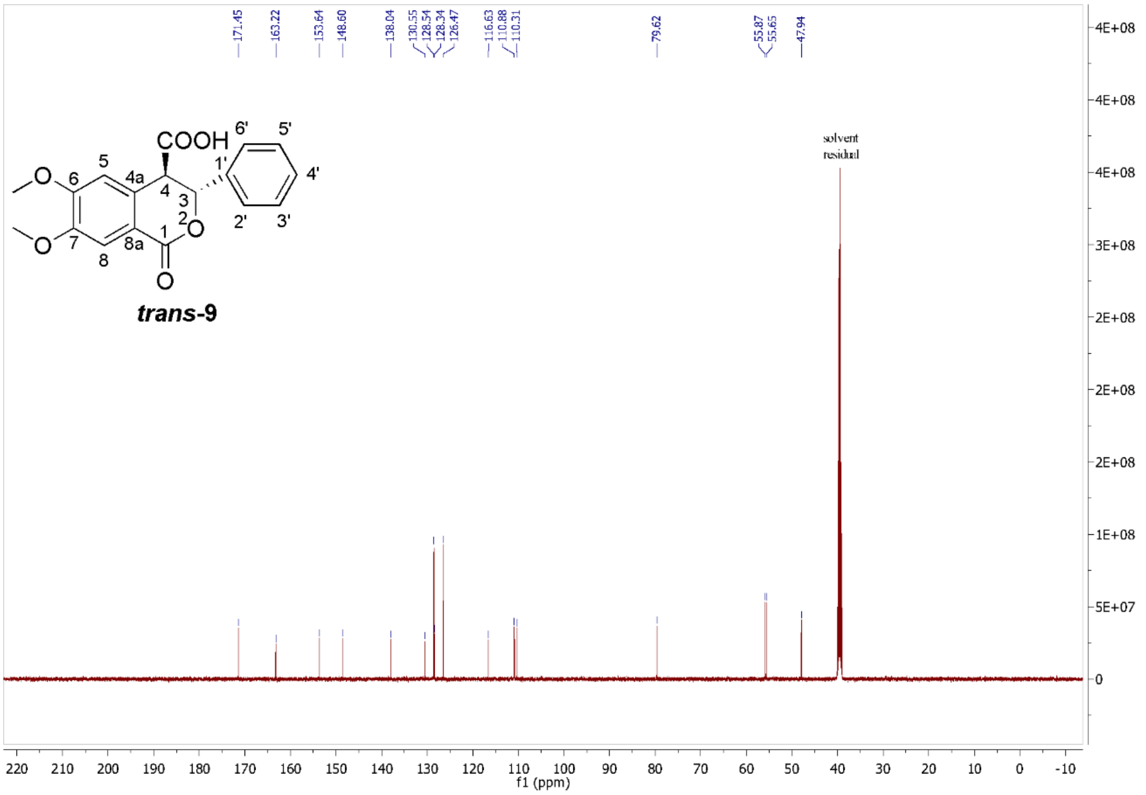

Figure S48. ^{13}**C-NMR Spectrum of** ***trans*****-9 in DMSO-d**$_6$**.**

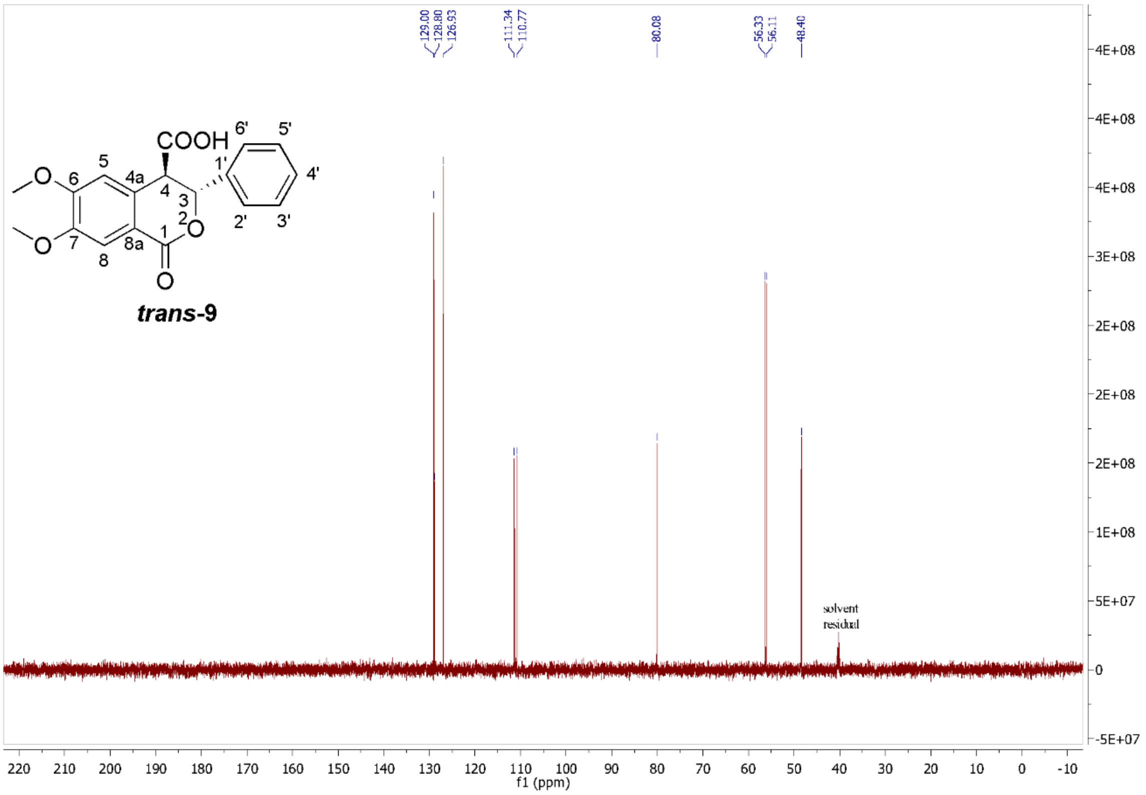

Figure S49. DEPT-135 NMR Spectrum of ***trans*****-9 in DMSO-d**$_6$**.**

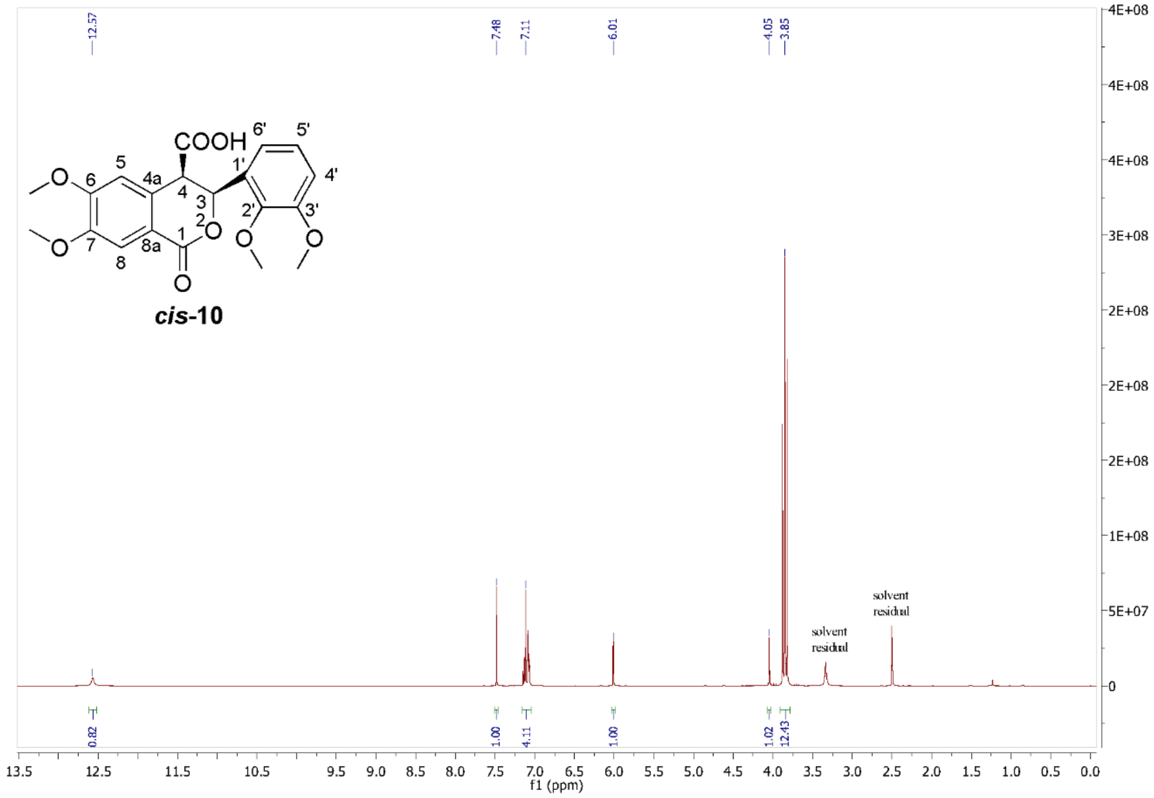

Figure S50. [1]**H-NMR Spectrum of** ***cis*****-10 DMSO-d6.**

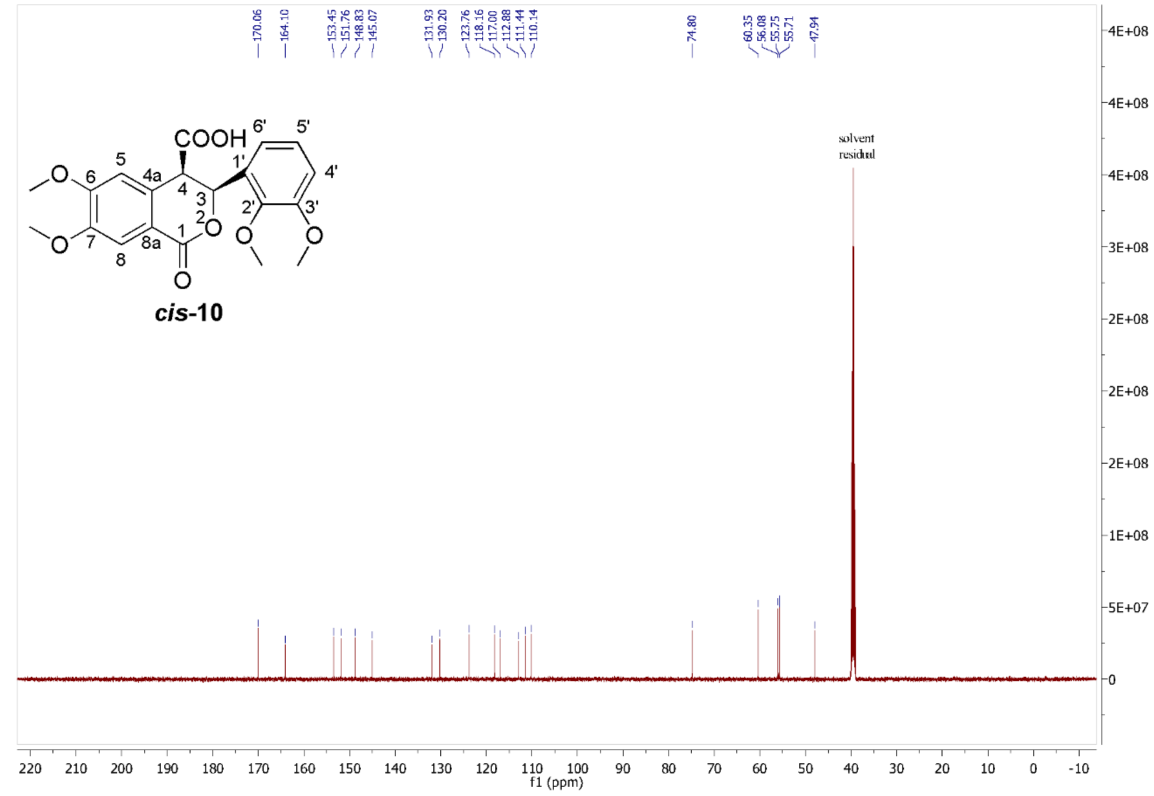

Figure S51. [13]**C-NMR Spectrum of** ***cis*****-10 in DMSO-d6.**

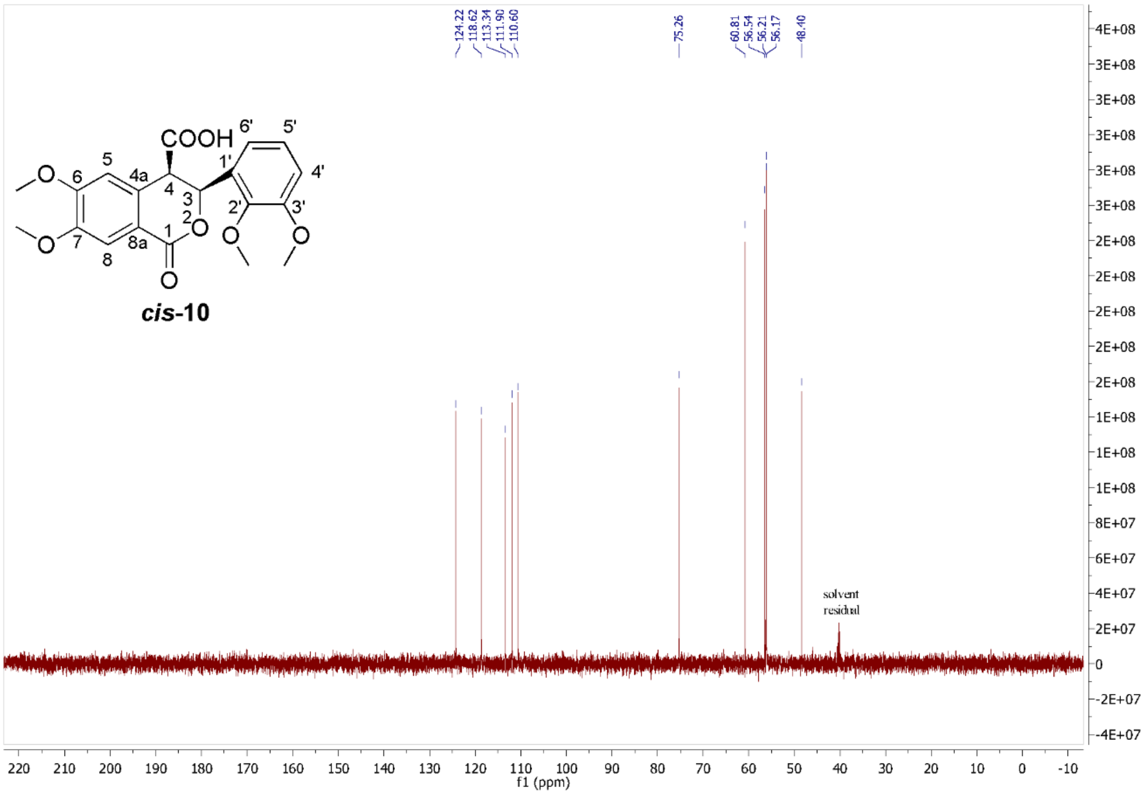

Figure S52. DEPT-135 NMR Spectrum of *cis*-10 in DMSO-d6.

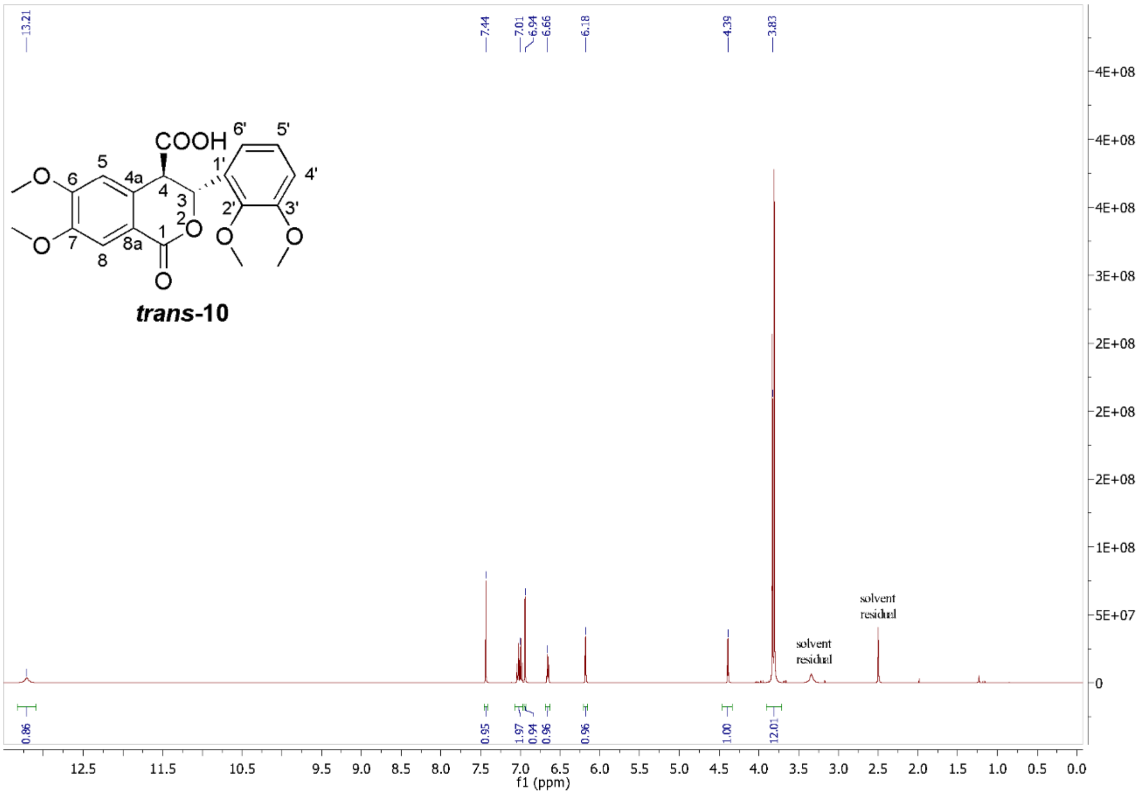

Figure S53. ^{1}H-NMR Spectrum of *trans*-10 DMSO-d6.

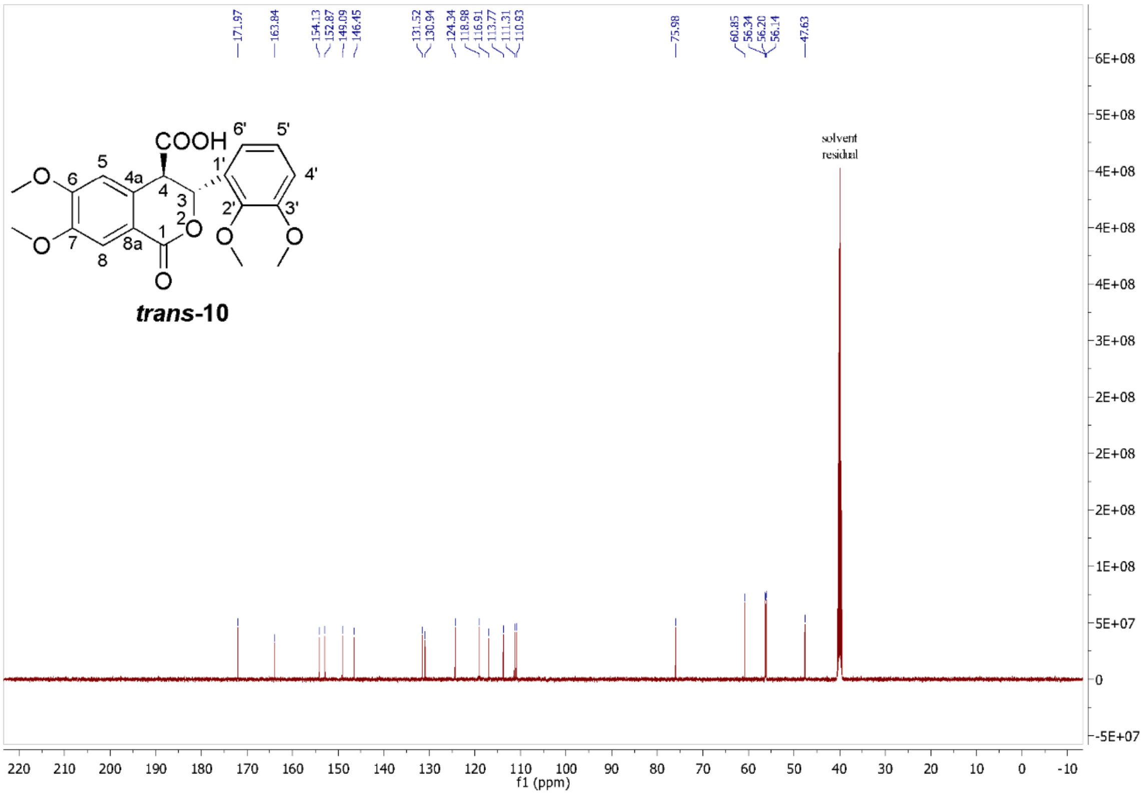

Figure S54. ^{13}C-NMR Spectrum of *trans*-10 in DMSO-d$_6$.

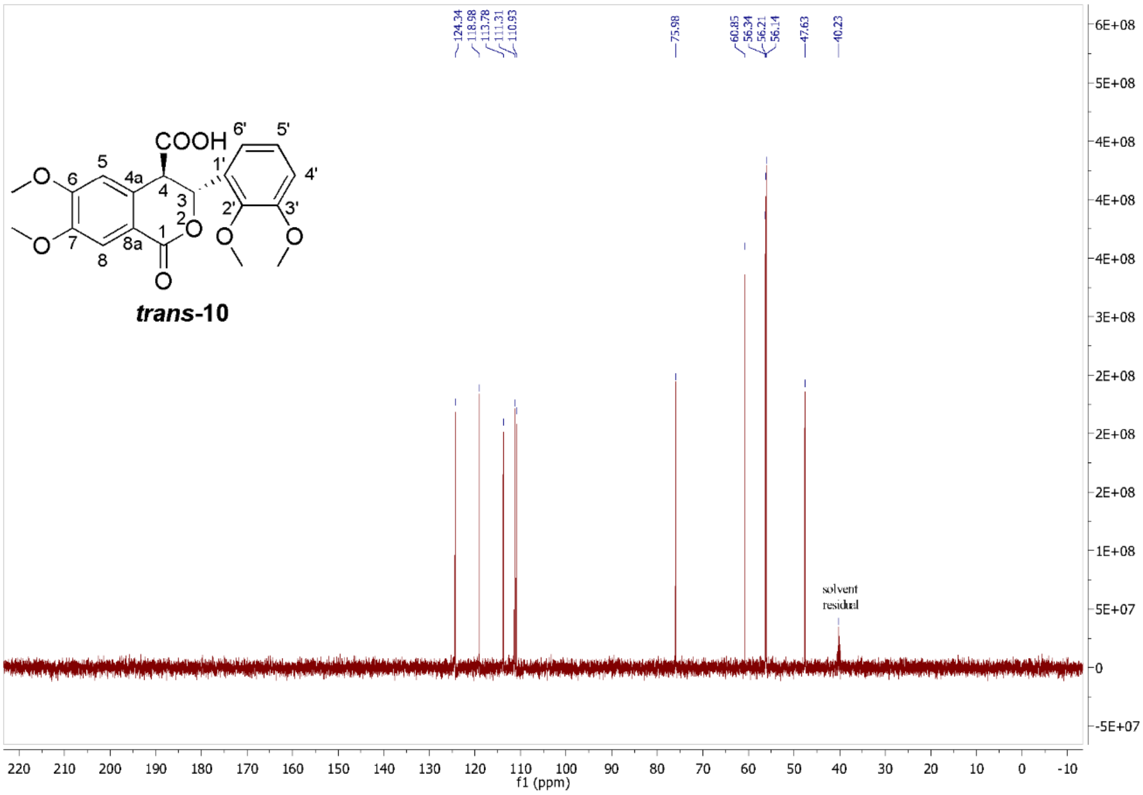

Figure S55. DEPT-135 NMR Spectrum of *trans*-10 in DMSO-d$_6$.

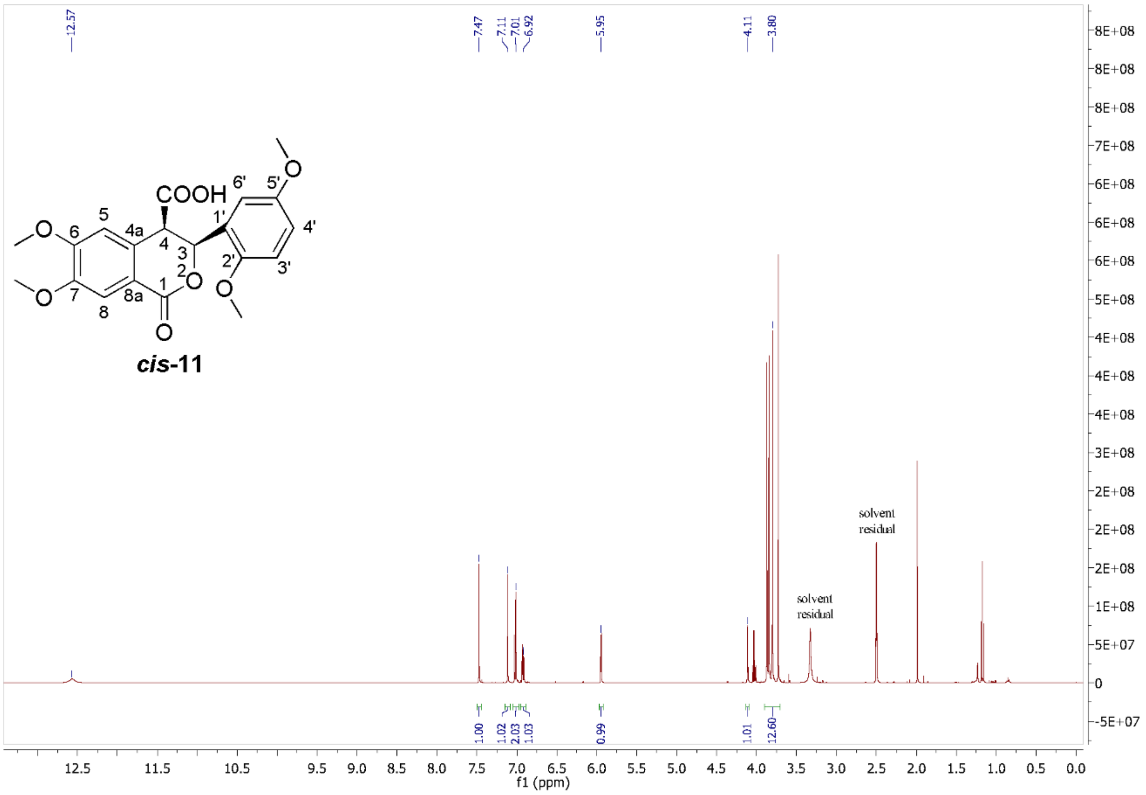

Figure S56. **^{1}H-NMR Spectrum of *cis*-11 DMSO-d6.**

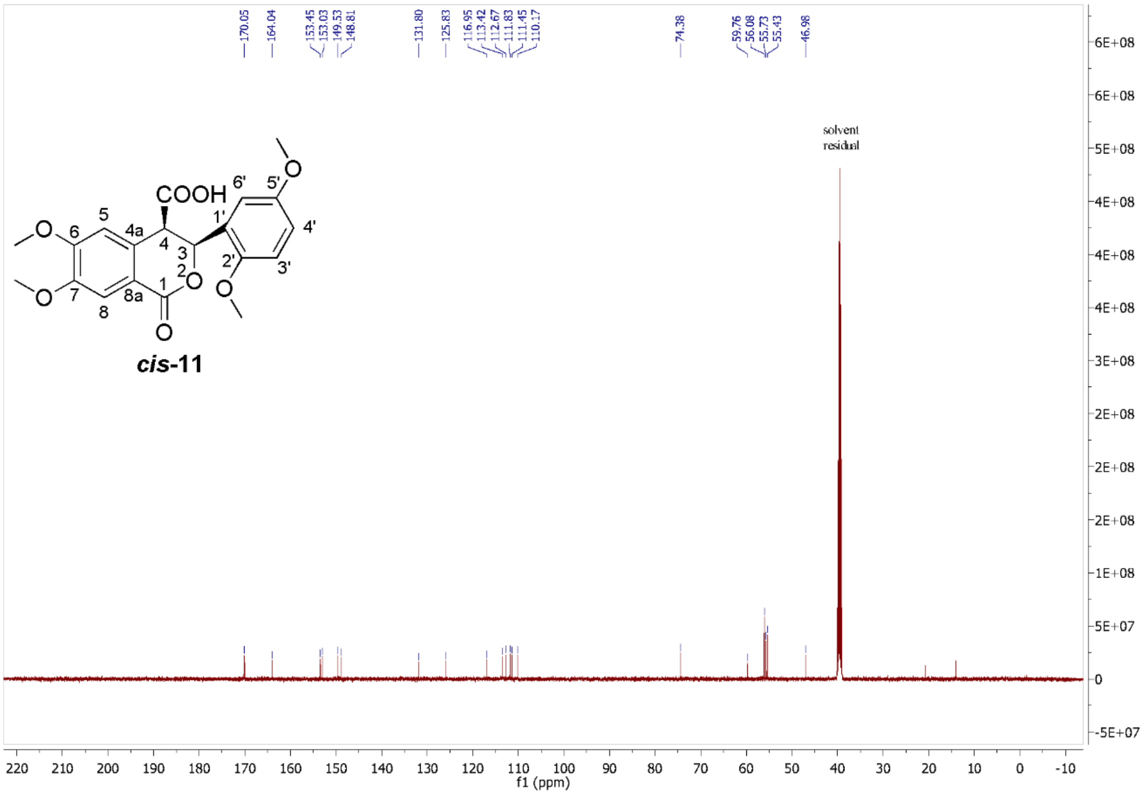

Figure S57. **^{13}C-NMR Spectrum of *cis*-11 in DMSO-d6.**

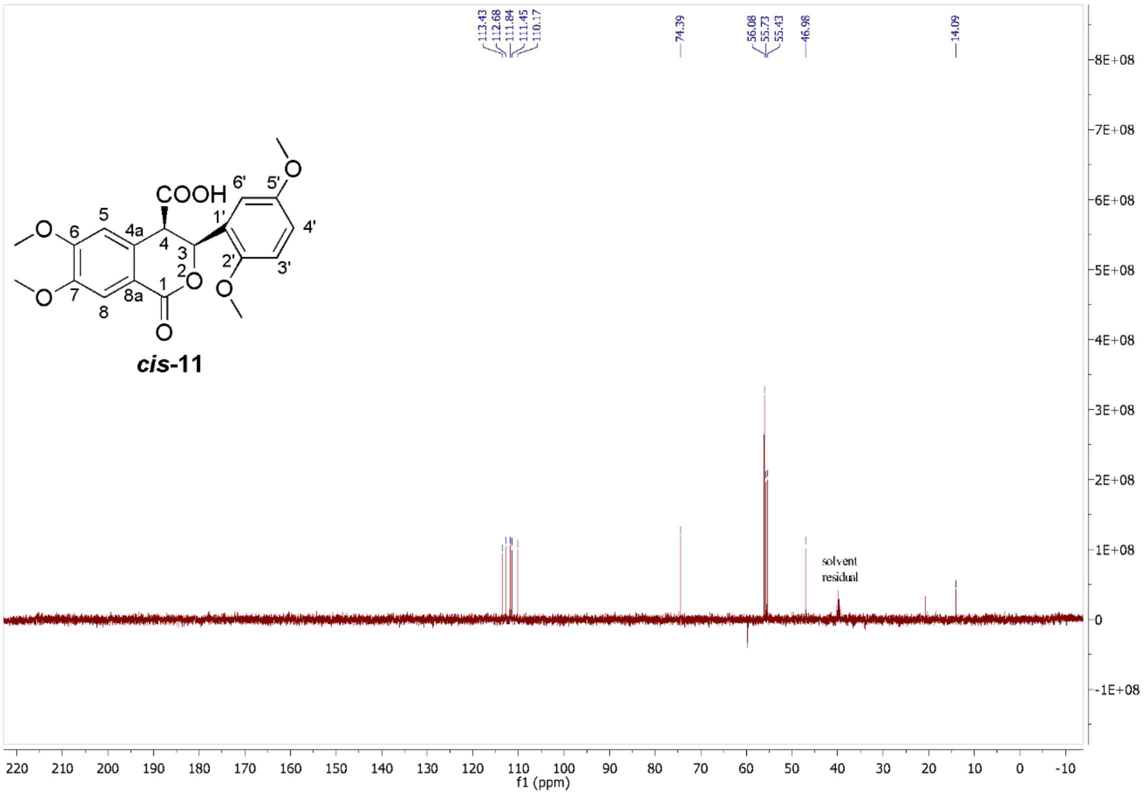

Figure S58. DEPT-135 NMR Spectrum of *cis*-11 in DMSO-d$_6$.

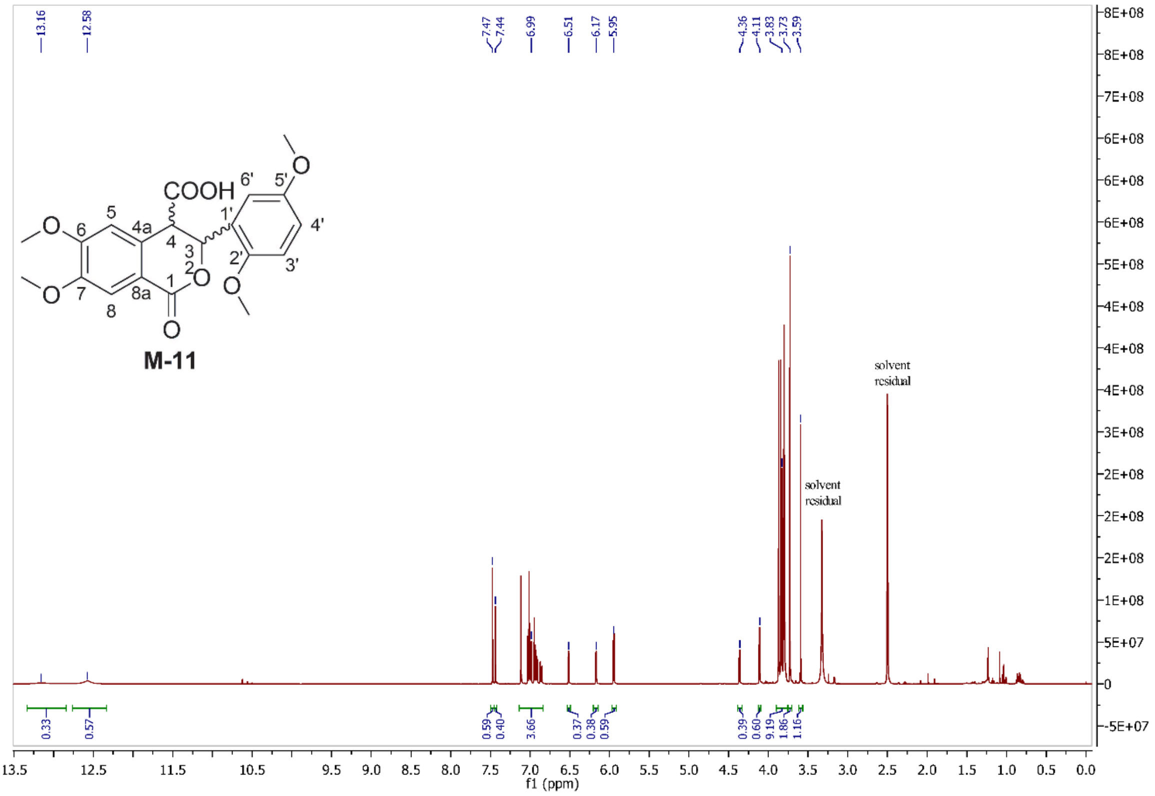

Figure S59. ^{1}H-NMR Spectrum of M-11 DMSO-d$_6$.
